# Molecular mechanism of calcium inhibition in viral channelrhodopsins

**DOI:** 10.1038/s41467-026-77716-5

**Published:** 2026-09-16

**Authors:** Dmitrii Zabelskii, Sergey Bukhdruker, Gerrit H. U. Lamm, Siarhei Bukhalovich, Mako Aoyama, Vsevolod Sudarev, Alexander Kuzmin, Mikihiro Shibata, Kota Kotayama, Hideki Kandori, Josef Wachtveitl, Ernst Bamberg, Valentin Gordeliy

**Affiliations:** 1 European X-ray Free Electron Laser GmbH, Schenefeld, Germany; 2 Research Center for Molecular Mechanisms of Aging and Age-related Diseases, Moscow Institute of Physics and Technology, Dolgoprudny, Russia; 3 Institute of Physical and Theoretical Chemistry, Goethe University Frankfurt, Frankfurt am Main, Germany; 4 Department of Life Science and Applied Chemistry, Nagoya Institute of Technology, Showa-ku, Nagoya, Japan; 5 WPI Nano Life Science Institute (WPI-NanoLSI), Kanazawa University, Kakuma-machi, Kanazawa, Ishikawa Japan; 6 Institute for Frontier Science Initiative, Kanazawa University, Kanazawa, Ishikawa Japan; 7 OptoBioTechnology Research Center, Nagoya Institute of Technology, Showa-ku, Nagoya, Japan; 8 Max Planck Institute of Biophysics, Frankfurt am Main, Germany; 9 Univ. Grenoble Alpes, CEA, CNRS, Institut de Biologie Structurale (IBS), Grenoble, France; 10 Present Address: European Molecular Biology Laboratory, Hamburg, Germany

**Keywords:** Ion transport, Bioenergetics, X-ray crystallography, Atomic force microscopy

## Abstract

Viral channelrhodopsins (VCR1s) are giant-virus-encoded light-gated channels permeable to monovalent and divalent cations, including Na^+^ and Ca^2+^ ions, and inhibited by millimolar Ca^2+^ concentrations. Here, we combine X-ray crystallography, time-resolved UV-vis spectroscopy, and ATR-FTIR spectroscopy to investigate molecular mechanisms of ion permeation and Ca^2+^-dependent inhibition in OLPVR1. An atomic resolution structure of OLPVR1 obtained in the presence of 10 mM CaCl_2_ and 900 mM NaCl reveals a transient intracellular Ca^2+^ binding site near T87 and T88, close to the retinal cofactor. Upon photoactivation, this Ca^2+^ ion prevents a key rearrangement of the intracellular gate required for ion translocation, namely the flip of E44, thereby disrupting ion conduction. Instead, illumination leads to the accumulation of Na^+^ ions between E44, S208 and the carbonyl oxygen of retinal-binding residue K204. Our findings reveal the molecular basis of Ca^2+^-dependent inhibition in VCR1s and provide a foundation for engineering enhanced tools for calcium optogenetics.

## Abstract (web summary)

Giant viruses encode light-gated channels that control calcium signals in infected cells. Here the authors reveal how calcium binds inside OLPVR1 and blocks the structural changes needed for ion flow.

## Introduction

Viral channelrhodopsins (VCR1s) are membrane photoreceptors that belong to the retinal-binding superfamily of microbial rhodopsins (MRs)^1–4^. Encoded by giant viruses, they form a distinct class of light-gated ion channels^5–7^ with broad ion conductivity, including monovalent and divalent cations, and an unusual inhibitory response to elevated (millimolar concentrations) extracellular calcium^7^. Functionally, VCR1s can release calcium from the endoplasmic reticulum (ER) without altering plasma membrane potential, making them attractive candidates for optogenetic applications. Light-induced ER calcium release by the prototypical VCR1 member OLPVR1 has been demonstrated in cultured cells and in vivo, where it triggers muscle contraction in tadpoles expressing OLPVR1^8^. Ecologically, VCR1s are widely distributed in giant viruses infecting phytoplankton and may influence host behavior and marine ecosystem dynamics by modulating light-dependent calcium signaling in infected cells^5,8^.

OLPVR1 has emerged as a particularly tractable model for understanding channelrhodopsin function. It expresses robustly in *E. coli* and forms highly ordered crystals that diffract to atomic resolution^7^. Recent structures of OLPVR1 in closed and open states in the presence of Na⁺ ions, determined at 1.1 and 1.3 Å resolution, respectively, provided an unusually detailed view of the retinal-binding pocket, ion-conduction pathway and hydration network in a light-gated ion channel^9^. These data positioned OLPVR1 as a powerful structural system for analyzing ion permeation in channelrhodopsins, akin to bacteriorhodopsin (*Hs*BR) for proton pumps^10–12^. However, the molecular basis of Ca²⁺-dependent inhibition, and its relationship to ion occupancy and channel-gate rearrangements, has remained unresolved.

The photoreaction of MRs is driven by structural rearrangements of the retinal cofactor, neighboring residues and highly coordinated water molecules. Atomic-resolution structures, typically better than 1.5 Å, are therefore critical for resolving alternative residue conformations, hydrogen-bonding geometries and ordered solvent networks^13,14^. The importance of high resolution data has been demonstrated for multiple rhodopsins, including *Hs*BR, MAR, KR2, *Sy*HR, *Bc*XeR, and *Cr*ChR2^12,15–19^. In both animal rhodopsins and MRs, the lysine-bound retinal chromophore carries a positive charge under physiological conditions, which is stabilized by negatively charged counterions^1^. This charge is central to light activation and ion transport in MRs^11,12,20,21^. Negatively charged ions such as chloride can bind near the retinal Schiff base (RSB^17,22^), whereas binding of positively charged ions such as Na⁺ has been observed mainly in intermediate-state structures of ion-pumping rhodopsins, such as KR2, where the RSB is deprotonated^16,21^. For ion-channeling MRs, atomic-resolution information on cation binding in ground and intermediate states has remained limited, with OLPVR1 providing a rare exception^9^.

Understanding ion translocation in membrane channels requires not only static structures but also structural information on key intermediate states along the conduction pathway. In potassium channels, the concept of single-file conductance^23^ established that transported ions occupy a limited number of discrete sites and move through the channel in a coordinated manner^24–26^. For light-gated ion channels, however, capturing transient ion-binding events remains challenging, limiting mechanistic understanding of conductance, selectivity and inhibition^27,28^. This is particularly important for OLPVR1, where Ca²⁺ inhibits channel activity despite the channel’s ability to conduct divalent cations. Therefore, resolving the intermediate states of the OLPVR1 photoreaction and inhibition would not only deepen our fundamental understanding of ion conductance and selectivity but also enable the rational engineering of OLPVR1 variants for optogenetic applications. Additionally, such insights may also be necessary to elucidate the physiological roles of VCR1s in virus–host interactions within marine ecosystems.

In this work, we combine atomic-resolution crystallography, time-resolved UV-vis spectroscopy, ATR-FTIR spectroscopy, high-speed AFM and molecular dynamics simulations to examine Ca²⁺-dependent inhibition of OLPVR1. The data identify Ca²⁺ binding within the channel pathway and show that Ca²⁺ binding is associated with altered intracellular-gate rearrangements and transient Na⁺ occupancy in the Ca²⁺-blocked state.

## Results

### Atomic-resolution structures of OLPVR1 in complex with Ca^2+^ ions

**Fig. 1:**
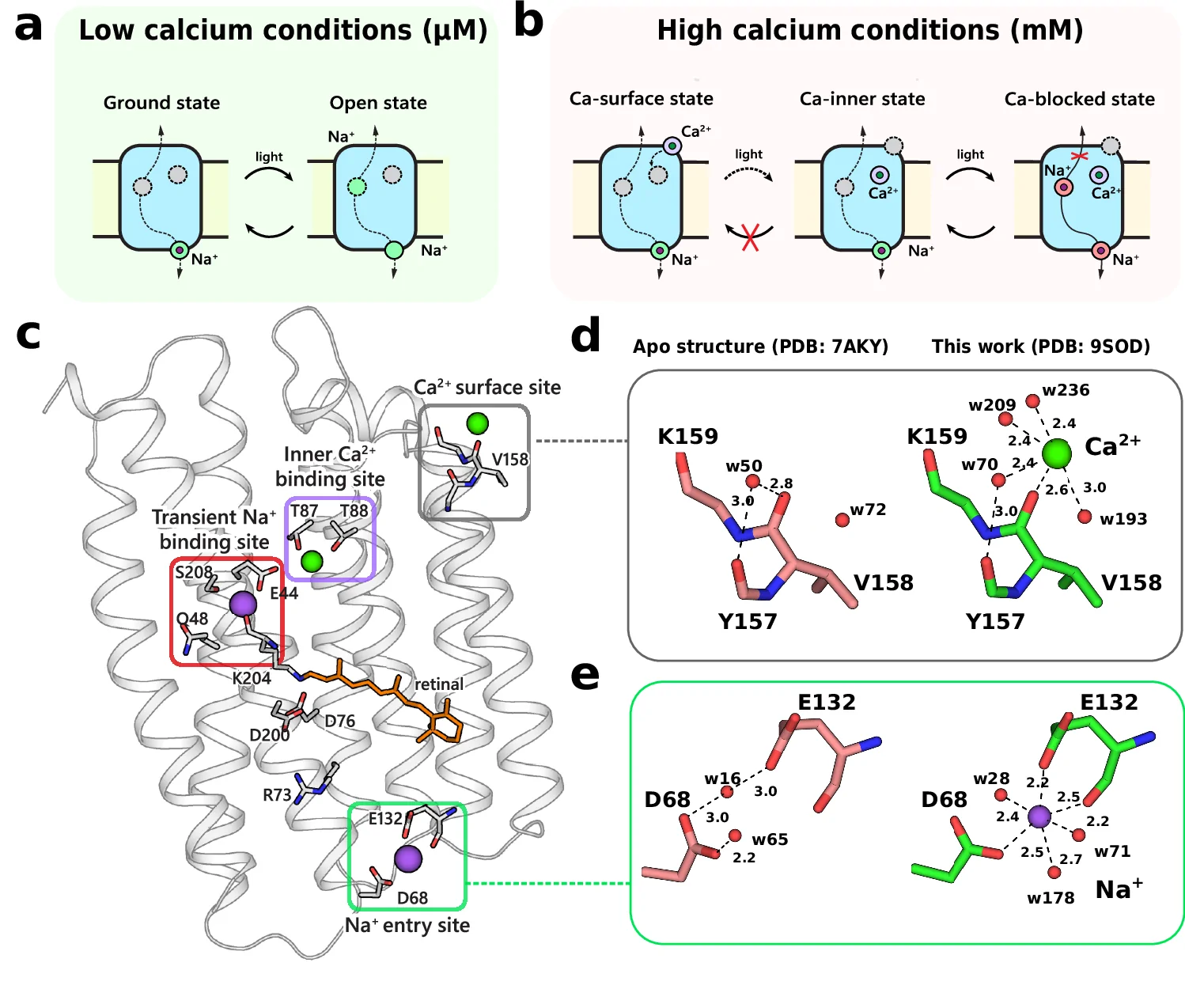
Influence of calcium on OLPVR1 function. **a** Schematic model of OLPVR1 channel activity under low calcium levels (left, μM Ca^2+^) and **b** under elevated calcium levels (right, mM Ca^2+^). **c** Key ion-binding regions of the OLPVR1 protein, identified in different states during the photocycle. **d** Magnified view of the Ca^2+^ binding site at the cytoplasmic side of the Ca-surface state structure. **e** Magnified view of the OLPVR1 sodium-binding site on the extracellular side of the protein. The corresponding region in the apo-structure of OLPVR1 (PDB ID: 7AKY^7^) is shown on the left for both binding sites. H-bonds are shown with dashed lines, and water molecules are represented by red spheres.

To obtain OLPVR1 structures in complex with calcium, we soaked Ca-free crystals in the crystallization buffer supplemented with calcium chloride. We employed two soaking protocols, which yielded calcium-bound structures of OLPVR1 referred to as the Ca-surface and Ca-inner states. For the Ca-surface state, crystals were soaked in 100 mM CaCl_2_ for 24 h in the dark. For the Ca-inner state, the crystals were soaked in 10 mM CaCl_2_ for 24 h under dim light illumination from a microscope lamp. Afterward, the crystals were left in the dark for an additional 30 minutes, then frozen in liquid nitrogen (LN_2_). Full details are available in Methods. The best crystals diffracted to resolutions of 1.45 and 1.41 Å, for the Ca-surface and Ca-inner states, respectively. Both structures were resolved in a P2_1_2_1_2 space group and contained one OLPVR1 molecule in an asymmetric unit. The structures of an OLPVR1 in a Ca-free (PDB ID: 9IN7^9^) and both calcium-bound states were found to be practically similar, with an RMSD of 0.2 Å. Identical to the Ca-free state, the calcium-bound structures also bind a sodium ion on the extracellular side of the protein. The sodium ion is coordinated by the side chains of D68 and E132, as well as multiple water molecules, w28, w71, and w178 (Fig. 1).

For the Ca-surface state, calcium was found to accumulate in the calcium-binding site on the exterior of the protein, between helices TM4 and TM5 (Ca^2+^ site 0). The calcium ion is coordinated by the main chain of the V158 residue and multiple water molecules (Fig. 1c). According to the CheckMyMetal server^29^, the calcium ion is observed in a free conformation with five atomic contacts. We also confirmed cation binding to this site by exchanging soaking salt with barium chloride. Barium is a divalent ion that yields a much stronger anomalous scattering signal. The 2.10 Å-resolution crystal structure of Ba-bound OLPVR1 (Ba-surface) provided a strong (5.9 σ-level) anomalous difference map peak at the same site (Supplementary Fig. 1). This site likely serves as an entry point for divalent ions from the intracellular side.

**Fig. 2:**
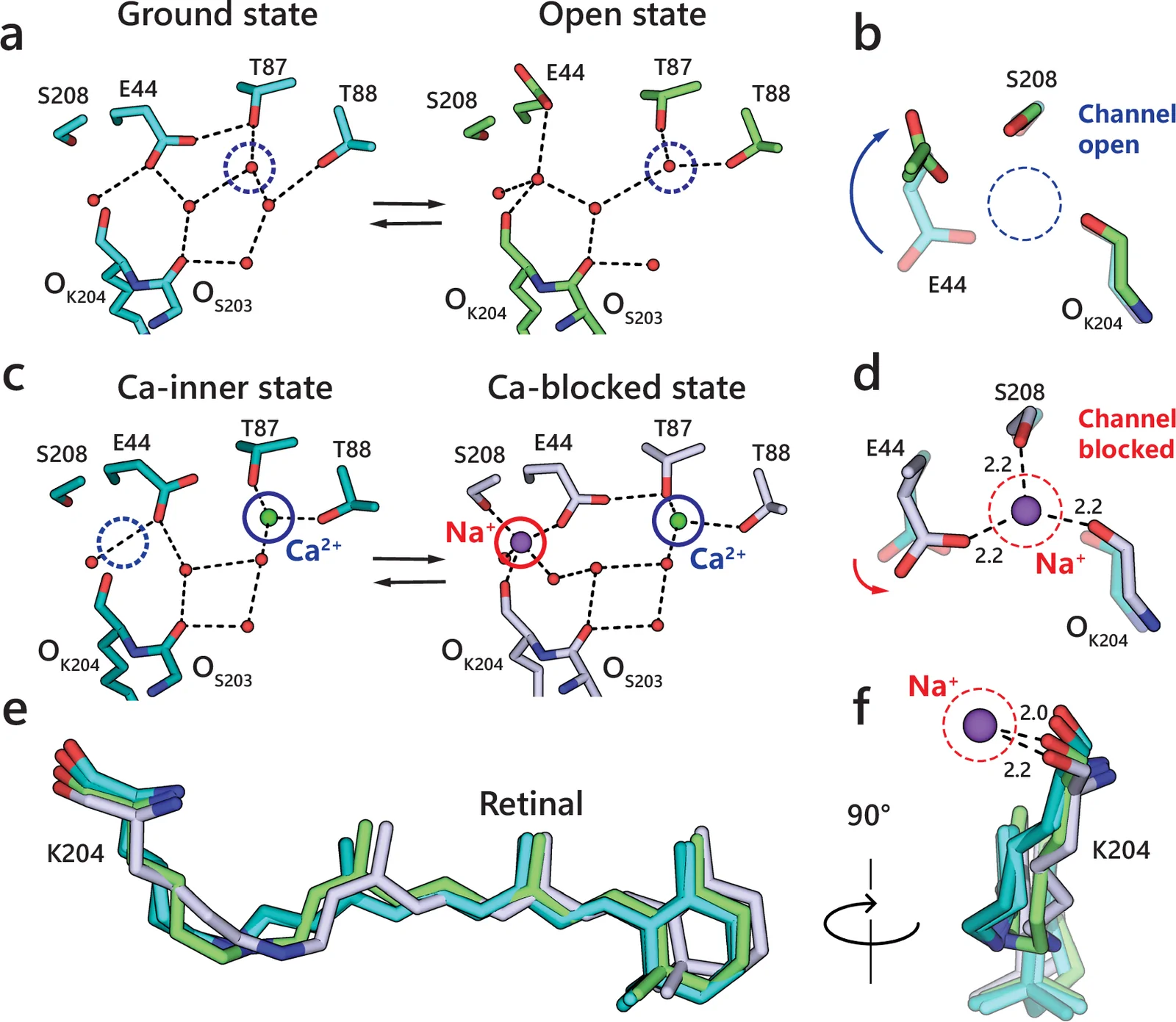
Binding of calcium ion inside OLPVR1 blocks the channeling pathway. **a** Magnified view of the structural rearrangements in the intracellular gate during channel opening in the absence of calcium. The ground (PDB ID: 9IN7^9^) and open-state (PDB ID: 9IN8^9^) structures are colored cyan and pale green, respectively. **b** Rearrangement of E44-K204-S208 triad during channel opening in the absence of calcium. **c** Magnified view of the Ca-inner (PDB ID: 9SOF) and Ca-blocked (PDB ID: 9SOG) states. The Ca-inner and Ca-blocked states are colored teal and light blue, respectively. **d** Close-up view of the sodium ion coordinated by the E44-K204-S208 triad. **e** Conformation of retinal cofactor and K204 residue in different structures. **f** Magnified view of the K204 residue and the coordination of the calcium ion by its carboxyl oxygen. H-bonds are shown with dashed lines, and red spheres represent water molecules.

The Ca-inner state, in contrast, lacks an exterior calcium density and instead features a Ca^2+^ binding site inside the protein within the channel pathway (Ca^2+^ site A). The calcium at the interior site is coordinated by the side chains of T87 and T88, the carboxyl oxygen of M84, and a water molecule w681 (Fig. 2). The Ca^2+^ assignment is supported by an 18.1 σ-level Fo^Ca-inner^-Fo^Ca-free^ peak near T87 and T88 and by Ca^2+^-coordinating conformations of these residues. In contrast, no anomalous signal from Ba^2+^ was detected at this site under the tested conditions, suggesting that Ba^2+^ does not occupy this internal site detectably in the present experiments and also indicating the possible limited Ba^2+^ conductance.

### Ion dependence of the OLPVR1 photocycle

**Fig. 3:**
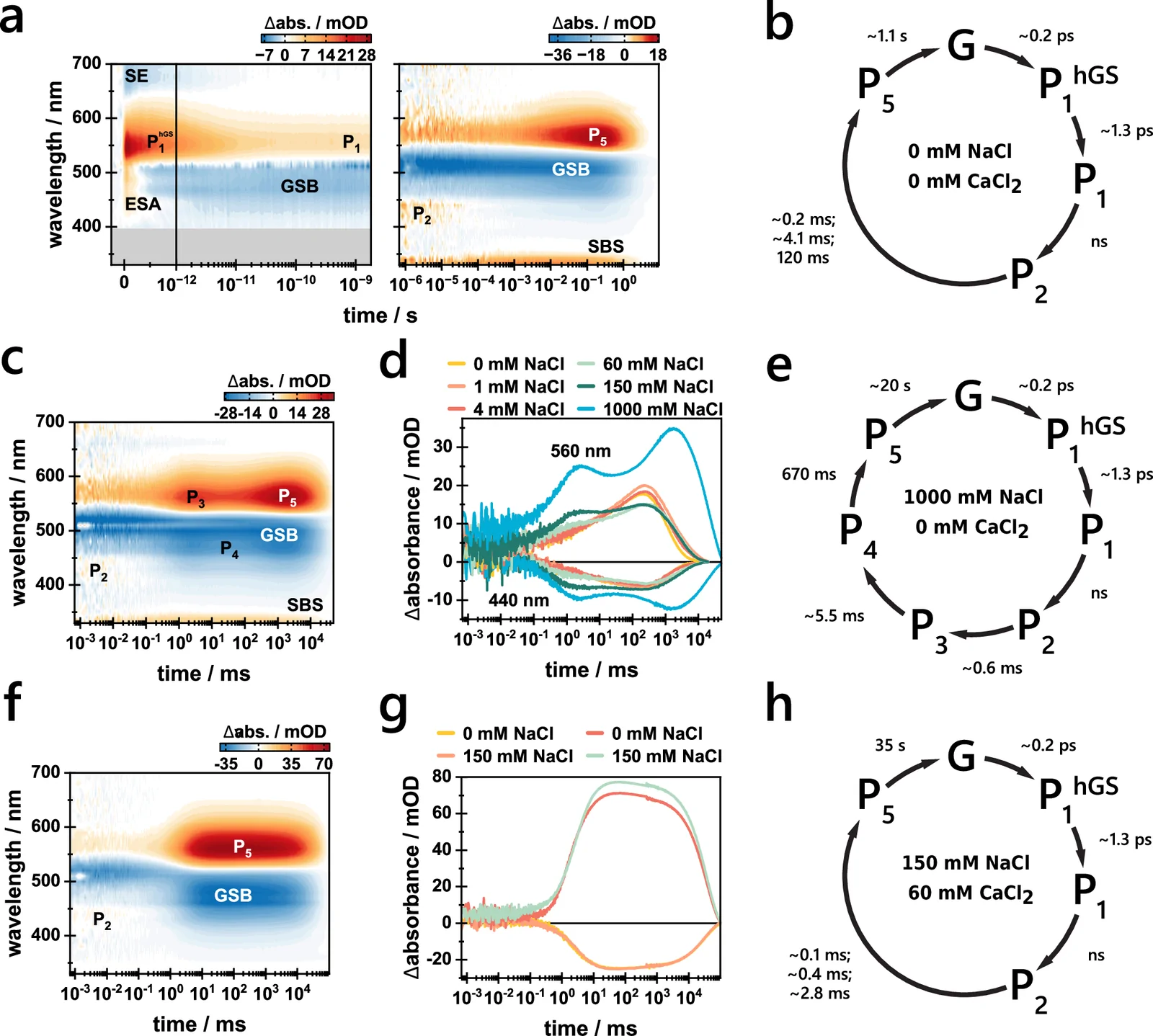
Ion-dependent spectroscopic characterization of OLPVR1. **a** Ultrafast transient absorption (TA) data, as well as flash-photolysis data for OLPVR1 at pH 8.0, 0 mM NaCl, 0 mM CaCl_2_, shown as 2D-contour plots with the signal amplitude color coded as follows: positive (red), no (white) and negative (blue) Δabs. For the ultrafast measurement, the time axis is linear until 1 ps and logarithmic afterward. The mentioned abbreviations stand for excited-state absorption (ESA), ground-state bleach (GSB), stimulated emission (SE), and second bright state (SBS)^97^. Photocycle intermediates are labeled as P_1_ to P_6_ in the order of their appearance. Gray areas indicate that no experimental data were obtained for these probing wavelengths. **b** The photocycle scheme of OLPVR1 at pH 8.0, 0 mM NaCl, and 0 mM CaCl_2_ was derived via kinetic analysis of the measurements shown in (**a**). **c** Flash-photolysis data of OLPVR1 at pH 8.0, 1000 mM NaCl, 0 mM CaCl_2_, are shown as a 2D-contour plot. **d** Sodium-dependent transients, obtained at 440 and 560 nm probing wavelengths in the absence of calcium. **e** The photocycle scheme was derived via kinetic analysis for OLPVR1 at pH 8.0, 1000 mM NaCl, and 0 mM CaCl_2_. **f** Flash-photolysis data of OLPVR1 at pH 8.0, 150 mM NaCl, and 60 mM CaCl_2_ are shown as a 2D-contour plot. **g** Comparison of flash-photolysis transients at probing wavelengths of 440 and 560 nm in the presence and absence of 150 mM NaCl. For both measurements, the buffer contained 60 mM CaCl_2_. **h** The photocycle scheme was derived via kinetic analysis for OLPVR1 at pH 8.0, 150 mM NaCl, and 60 mM CaCl_2_.

To better understand the differences in ion interactions with the protein for sodium and calcium ions, we investigated the dependence of the OLPVR1 photocycle on these ions at both ultrafast and flash-photolysis timescales. The ultrafast dynamics of OLPVR1 (Fig. 3) resemble those of VirChR1^30^. Neither spectral nor pronounced kinetic differences in the presence of either sodium or calcium ions were observed. Under all investigated conditions, the excited-state signatures decay on a sub-ps timescale, accompanied by the formation of a first photocycle intermediate P_1_^hGS^, which subsequently relaxes at ~1 ps to form the first stable photocycle intermediate P_1_ located at ~555 nm (Supplementary Fig. 2). The second observed blue-shifted intermediate state, P_2_, appears on the nanosecond timescale. To bridge the temporal gap between ultrafast and flash-photolysis measurements, an additional dataset covering the 200 ps–10 µs range was recorded using synchronized fs-laser systems (Supplementary Fig. 2). The following steps of the photocycle have been identified as dependent on the presence of sodium or calcium ions, respectively.

Initially, the sodium-dependent effects are discussed, and the numbering of the observed intermediate states is adjusted to accommodate the presence of both ions. For low sodium concentrations (0–4 mM NaCl), a multiphasic transition (lifetimes: ~0.2, ~4.1, and ~120 ms) of the blue-shifted P_2_ intermediate takes place to form the final red-shifted photocycle intermediate P_5_ (Fig. 3b). The photocycle is completed within ~1.1 s. Once the sodium concentration reaches 60 mM NaCl, two additional intermediates are observed: a red-shifted P_3_ state and a blue-shifted P_4_ state, which become more temporally separate from the P_5_ state at a 1000 mM NaCl concentration with a time constant of ~670 ms. Subsequently, the parent state is repopulated after ~20 s. Accordingly, the OLPVR1 photocycle is strongly sodium-dependent, with the sodium-saturated photocycle consisting of six different intermediate states, two of which are directly formed in the presence of a sufficient amount of sodium ions (Fig. 3c–e and Supplementary Figs. 3, 4). Similar to other ion-binding rhodopsins, the sodium binding of OLPVR1 is highly sensitive to sodium concentration, and its affinity constant likely exceeds 100 mM^31,32^. Notably, the crystallization conditions used in the study contain 900 mM NaCl, indicating that the conditions are saturated^9^.

The calcium-dependent effects on the photocycle of OLPVR1 were investigated at concentrations of 0 mM and 60 mM CaCl_2_ (Fig. 3a, f). The CaCl_2_ concentrations were chosen based on the calcium-dependent activity data for VirChR1, where 0 mM CaCl_2_ represents the open state and 60 mM CaCl_2_ represents the blocked state^30,33^. Similar to the sodium-dependent effects, the presence of calcium affects the later photocycle steps; namely, the red-shifted intermediate P_5_ was observed in the absence of both sodium and calcium. The addition of calcium causes an increase in the intensity of the late red-shifted intermediate states, which also exceeds all sodium-related effects discussed in the section above. This is evidenced by comparing measurements done in the presence of 60 mM CaCl_2_ and 0 mM or 150 mM NaCl (Fig. 3g). The ground-state bleach (GSB) amplitude is almost identical at both investigated sodium concentrations. Therefore, it can be concluded that the increased intensity of the P_5_ intermediate at 150 mM NaCl, compared to 0 mM NaCl, is likely due to the binding of both sodium and calcium, with calcium effects being more dominant because calcium binding is already saturated. Besides, the addition of 60 mM CaCl_2_ does not affect the OLPVR1 photocycle at pH 3, indicating the light dependence of the OLPVR1-calcium interaction (Supplementary Note, Supplementary Fig. 5). To conclude, the main observed calcium-dependent effects are an increased P_5_ intermediate amplitude and a longer photocycle, resulting in the last photocycle intermediate decaying with a lifetime of ~35 s (Fig. 3h). Furthermore, the observed calcium and the previously discussed sodium-related effects need to be reversible, as no accumulation of calcium- and/or sodium-bound protein species was observed during the experiments.

### Infrared spectroscopy reveals structural changes of OLPVR1 upon calcium binding

**Fig. 4:**
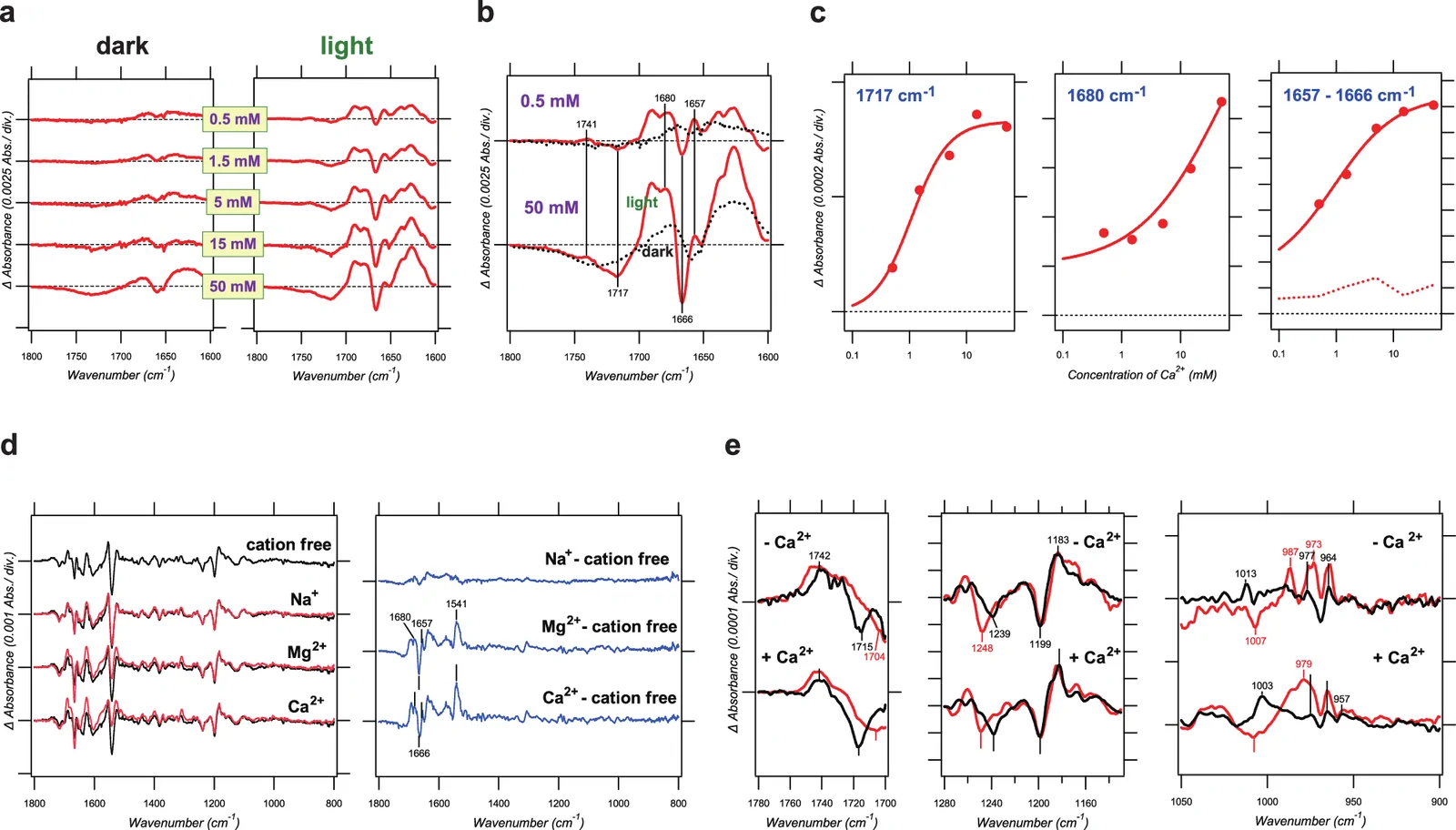
Calcium binding to OLPVR1 monitored by FTIR spectroscopy. **a** The presence-minus-absence difference FTIR spectra of various Ca^2+^ concentrations for OLPVR1 under dark (Left) and light (Right) conditions. Difference spectra were obtained using a buffer-exchange system at pH 8 and 283 K, with the sample illuminated at 501 nm. **b** The presence-minus-absence difference FTIR spectra of 0.5 mM (Top) and 50 mM (Bottom) Ca^2+^ under dark (black dotted line) and light (red line). **c** Typical IR band intensities at 1717 cm^−1^ (C=O stretch of protonated carboxylic acids), 1680 cm^−1^, and 1657–1666 cm^−1^ (amide I of α-helix) against Ca^2+^ concentration. The difference between light and dark was plotted at 1712–1722 cm^−1^ and 1680 cm^−1^. On the other hand, the difference of the 1657 (+) cm^−1^ minus 1666 (−) cm^−1^ bands was plotted for both dark (dotted line) and light (red circles). Solid lines were obtained from curve fitting using the Hill equation. While Kd values were not estimated precisely for the 1680-cm^−1^ band (*K*_d_ > 50 mM), *K*_d_ values were determined to be 1.1 mM (1717 cm^−1^) and 0.93 mM (1657–1666 cm^−1^). **d** Light-minus-dark difference FTIR spectra of OLPVR1 in the absence (black line) and presence (red line) of 100 mM NaCl, 5 mM MgCl_2_, and 5 mM CaCl_2_ (Left). Spectra were measured in H_2_O at pH 8 and 283 K. Double-difference spectra between the presence and absence of ions (Right). **e** Light-minus-dark difference FTIR spectra of OLPVR1 in the absence (top) and presence (bottom) of 5 mM CaCl_2_. Spectra were measured in H_2_O (black) and D_2_O (red) at pH 8 and 283 K.

In the previous chapters, we demonstrated the existence of a calcium-binding site within the OLPVR1 channel (site A). To gain a more detailed understanding, we applied difference IR spectroscopy to OLPVR1, in which purified protein was reconstituted into lipids and attached onto the ATR-FTIR crystal^34,35^. Here, we performed two experimental modes: (i) the presence-minus-absence difference of Ca^2+^ by the flow system, or (ii) the light-minus-dark difference (Supplementary Fig. 6) at pH 8 and 283 K. The left panel in Fig. 4a shows the presence-minus-absence difference FTIR spectra of various Ca^2+^ concentrations under dark conditions. Although spectral changes were observed under dark conditions, much more prominent peaks were monitored under light conditions (at 501 nm; right panel of Fig. 4a). In particular, differences are evident in the amide I region (1700–1600 cm^−1^), which monitors secondary structure. Specific binding of Ca^2+^ likely occurs only under light, presumably by late intermediates. Figure 4b compares the difference spectra upon Ca^2+^ binding between dark and light, where a negative peak at 1717 cm^−1^, together with a small positive peak at 1741 cm^−1^, indicates perturbation of a carboxylate. From the titration curve of the light-minus-dark value at 1717 cm^−1^ (average over 1712–1722 cm^−1^), the Kd was determined to be 1.1 mM (Fig. 4c). On the other hand, positive peaks at 1700–1670 cm^−1^ are more pronounced at high Ca^2+^ concentrations, with a *K*_d_ value greater than 50 mM (1680 cm^−1^, Fig. 4c). Amide-I vibration at 1660–1650 cm^−1^ is the characteristic frequency of α-helix, and the peak pair at 1666 (−)/1657 (+) cm^−1^ represents deformed and normal α-helix in the Ca^2+^-free and -bound states, respectively (Fig. 4b). By the titration curve of this region, the *K*_d_ value was determined to be 0.93 mM (Fig. 4c). From ATR-FTIR by flow system (Supplementary Fig. 6a), we found no specific binding of Ca^2+^ to OLPVR1 under dark conditions, but Ca^2+^ binding with two *K*_d_, 1 mM and >50 mM, under light conditions. High-affinity binding (*K*_d_ ~1 mM) occurs in conjunction with the deprotonation of the carboxylate and an α-helical rearrangement.

We then measured light-induced difference FTIR spectra in buffers containing various cations (Supplementary Fig. 6b). The left panel in Fig. 4d shows the light-minus-dark difference FTIR spectra in the absence (black line) and presence (red line) of 100 mM NaCl, 5 mM MgCl_2_, and 5 mM CaCl_2_. Under all conditions, a peak pair was observed at 1199 (−)/1183 (+) cm^−1^, which indicates that OLPVR1 and the photointermediate contain protonated all-*trans* and 13-*cis* retinal, respectively. Light-minus-dark difference FTIR spectrum with 100 mM NaCl coincides with that of the cation-free form, which is clearer from the flat double-difference spectrum (Right panel in Fig. 4d). This is consistent with the fact that transported ions are not bound specifically within rhodopsin, as recently reported for a potassium channelrhodopsin^35^. In contrast, a clear spectral difference was observed between Mg^2+^ and Ca^2+^, and the double-difference spectra exhibit peaks at 1680, 1666, and 1657 cm^−1^ (right panel in Fig. 4d), which were also observed in Fig. 4b. Supplementary Fig. 7 compares light-minus-dark difference spectra between the Ca^2+^-free and -bound forms, with some of them enlarged in Fig. 4e. Spectral features of C-C stretch at 1250–1150 cm^−1^ are identical with and without Ca^2+^, suggesting a distant binding site from the retinal chromophore. Nevertheless, we observed a clear spectral difference in the HOOP region that monitors chromophore distortion. Broad positive peaks at 1003 cm^−1^ in H_2_O and at 979 cm^−1^ in D_2_O are characteristic of Ca^2+^-bound OLPVR1, which were not observed for the Ca^2+^-free form (Fig. 4e). Ca^2+^-dependent chromophore distortion in late intermediate(s) suggests the presence of a network between the binding site and the retinal.

At the 1780–1700 cm^−1^ region, we observed a peak pair at 1742 (+)/1715 (−) cm^−1^ in the absence of Ca^2+^. Interestingly, while the negative peak exhibits a downshift to 1704 cm^−1^ in D_2_O, the positive peak at 1742 cm^−1^ seems to appear in H_2_O and D_2_O. This suggests the protonated carboxylic acid is in an unusual environment in late photointermediate(s). Similar spectral features were observed in the presence of Ca^2+^, but the negative peak at 1715 cm^−1^ in H_2_O (at 1704 cm^−1^ in D_2_O) is more pronounced. This observation suggests that two carboxylic acids are protonated, one of which exhibits a change in H-bond frequency (from 1715 cm^−1^ to 1742 cm^−1^). In addition, another carboxylic acid deprotonates upon formation of the intermediate in the presence of Ca^2+^. The frequency at 1715 cm^−1^ in OLPVR1 is consistent with the measurement in Fig. 4b (1717 cm^−1^). A recent difference FTIR study of OLPVR1 mutants at 77 K identified the 1722 cm^−1^ band as the C=O stretch of protonated E51^36^. Although the vibrational frequency of E44 was not assigned in the paper, a difference FTIR study of OLPVR2^37^ at 77 K reported a carboxylic C=O stretch at 1714 cm^−1^. OLPVR2 lacks E51 but possesses E44 (E42 in OLPVR2), and because of similar microenvironments between E44 in OLPVR1 and E42 in OLPVR2, E44 is probably protonated in OLPVR1, whose C=O stretch is located near 1714 cm^−1^. We then applied flow-based FTIR spectroscopy of the presence-minus-absence difference to E44Q and E51Q at various Ca2+ concentrations. Unlike the wild-type, both E44Q and E51Q exhibited no Ca^2+^ binding even under light conditions (Supplementary Fig. 8), indicating important roles of E44 and E51 for Ca^2+^ binding.

### Atomic-resolution structure of the calcium-blocked photoactivated state of OLPVR1 reveals its inhibition mechanism

To understand the mechanism of calcium-dependent inhibition of OLPVR1, we used a cryotrapping method to recreate channel opening in the presence of calcium. For the procedure, we prepared the Ca-inner state crystals as described above and followed a protocol similar to that used to obtain the open state of OLPVR1 (PDB ID: 9IN8^9^; see Methods for full details). Hereafter, for simplicity, we refer to this cryotrapped photoactivated state as Ca-blocked.

**Fig. 5:**
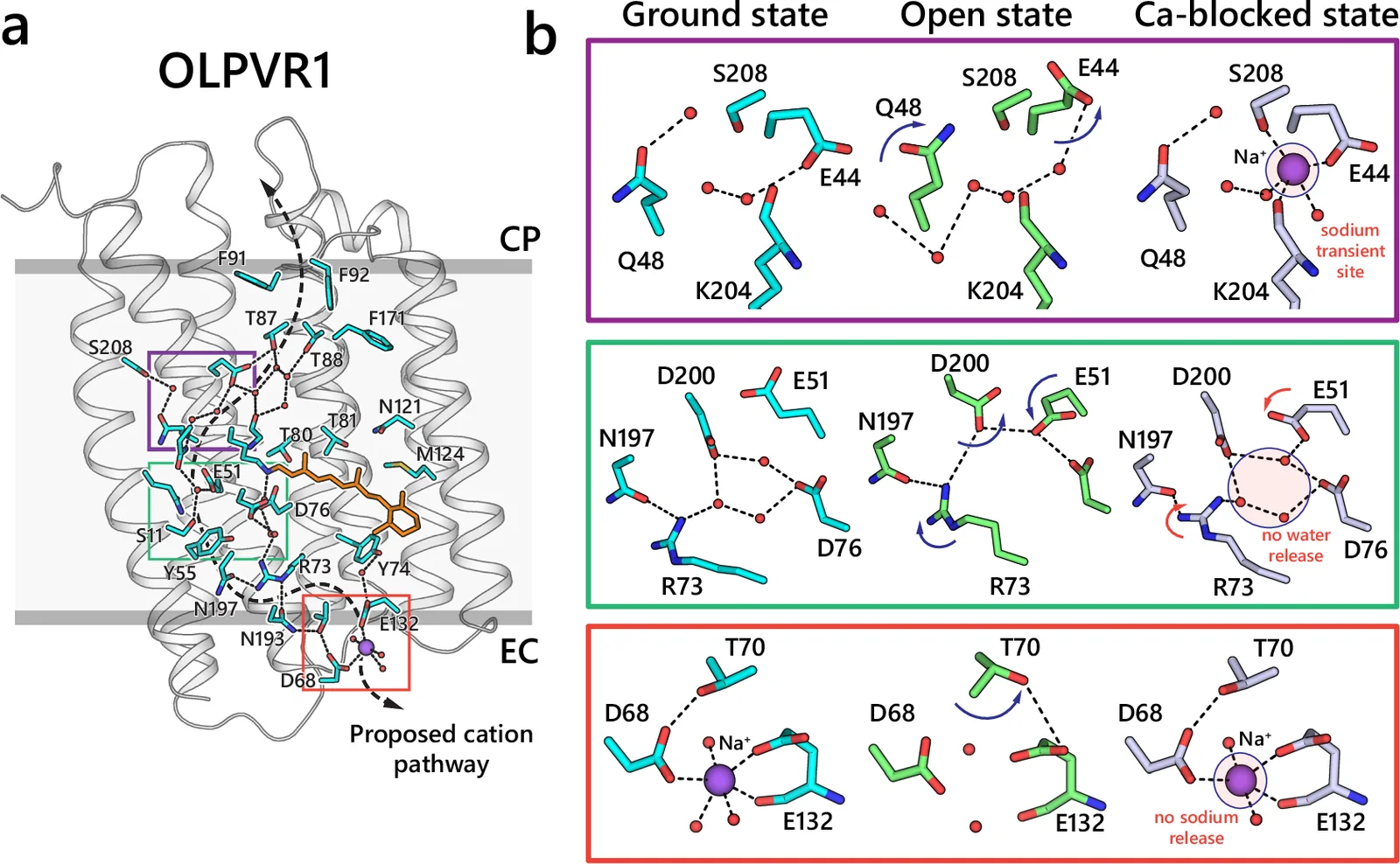
Key molecular switches of OLPVR1 channelrhodopsin upon photoactivation. **a** Overall view of the OLPVR1 structure in the calcium-free ground state (PDB ID: 9IN7^9^). EC/CP refer to the extracellular and cytoplasmic sides of the membrane, respectively. **b** The intracellular sodium-binding site in the ground (PDB ID: 9IN7), open (PDB ID: 9IN8), and Ca-blocked (PDB ID: 9SOG) states are shown on the left, middle, and right panels of the top row, respectively. Structural conformations of the key residues in the RSB region of OLPVR1 are shown for the three respective structures in the middle row. The extracellular binding site of OLPVR1 is shown for the three structures in the bottom row. The OLPVR1 in the ground, open, and Ca-blocked states are colored cyan, pale green, and blue-white, respectively. H-bonds are shown with dashed lines, and water molecules are represented by red spheres.

The best Ca-blocked crystals diffracted to 1.52 Å resolution and, according to Xtrapol8^38^, yielded the 30% occupancy of the state, which is in good agreement with UV-Vis measurements on crystals (Supplementary Fig. 9). While the ground (not photoactivated) states of OLPVR1 structures under different conditions are nearly identical, except for ion-binding sites, the Ca-blocked state differs drastically from the open state in both configuration of the ion-gating residues and the ion binding (Fig. 5). While the open state structure features no ion-associated electron densities, the Ca-blocked state structure features three ions bound inside the protein: one Ca^2+^ ion and two Na^+^ ions. As previously proposed for the OLPVR1 mechanism^9^, in the absence of calcium ions, the light activation causes the release of a sodium ion from the D68-E132 binding site, accompanied by the rotation of the side chain of T70 and the rearrangement of the H-bond network in the extracellular side of the protein. In contrast, no sodium release and no T70 rotation were observed in the Ca-blocked state, indicating that the sodium ions are fixed at the entrance since the second sodium ion is trapped inside the channel because the channel is blocked by the Ca^2+^ ion (Fig. 5b). It also suggests that the ions within the channel interact with one another.

In the retinal Schiff base (RSB) region, we observe the absence of three coordinated water molecules (w9, w15, and w18) in both open and Ca-blocked states. However, the configuration of the gating residues is different. Upon opening the channel, the E51 side chain breaks its interaction with the N205 residue, triggers the release of RSB water, and forms H-bonds with the D76 and D200 counterions. Both D76 and D200 residues rearrange their side chains to form an interconnected triad with the E51 residue (E51-D76-D200). However, in the Ca-blocked state, neither D76 nor D200 rearranges, and E51 is found in a partially flipped conformation that prevents the formation of the triad (Fig. 5b). In this configuration, the E51 and D200 side chains are 3.4 Å apart, compared with 2.6 Å in the open state. While the 2.6 Å distance corresponds to a strong H-bond interaction, the 3.4 Å bond is a weak electrostatic H-bond. This indicates a partial loss of coordination between the residues caused by calcium binding. In the Ca-blocked state, the D200 side chain forms a strong 2.6 Å H-bond with the R73 side chain, whereas in the open state, the H-bond between D200 and R73 is absent. Besides that, in the Ca-blocked structure, the side chain of R73 undergoes an additional rotation of its guanidine group, which results in the formation of a strong 2.1 Å H-bond between R73 and N197 side chains, which is not observed in the open state (Fig. 5b).

In the intracellular side of the open state structure, the ion pathway opening is initiated by the side chain flips of the Q48 and E44 residues, which also result in the appearance of two additional transiently bound water molecules (w1’ and w561’) that occupy the available space^9^. In the Ca-blocked state, both E44 and Q48 are not flipped and are found in the ground-state-like conformation.  Notably, the calcium ion in site A, coordinated by the side chains of T87 and T88, is observed in the same conformation in the blocked state, without any signs of the ion leaving this site.

Moreover, we identified a 6.8 σ-level electron density peak on the F_O_^Ca-blocked^-F_O_^Ca-inner^ difference map in the proposed ion pathway near the carbonyl group of K204, which is associated with the corresponding lack of rearrangement of the E44 side chain, and is not observed in the open state structure (Supplementary Fig. 10). This electron density is located at the channel constriction, between E44, S208, and the carbonyl oxygen of K204, with a corresponding distance of 2.2 Å, indicating a strong electrostatic interaction. Using 500-ns-long molecular dynamics simulations, we tested three potential candidates for this site: a water molecule, a calcium ion, and a sodium ion (Supplementary Fig. 11). We observed that the calcium ion at site B induces a significant shift in charge distribution within the protein, attracting nearby water molecules and thereby destabilizing the calcium ion at site A, which then vacates its site within 150 ns across all runs. In contrast, both the sodium ion and the water molecule remained close to their starting positions throughout the simulation, indicating that they remained stable at site B. However, the average H-bond distances for the water molecule and the sodium ion are approximately 2.7 and 2.3 Å, respectively^39,40^. Besides that, the analysis of E44 side chain fluctuations indicates that the sodium ion induces the smallest fluctuations among the three candidate molecules, further pointing towards the presence of sodium in site B (Supplementary Note and Supplementary Fig. 12). While we cannot directly rule out the presence of a water molecule at site B, we believe the electron density is more likely to correspond to a sodium ion, given both its high coordination and strong hydrogen-bond interactions with neighboring residues.

### OLPVR1 forms a dimer in the membrane

Microbial rhodopsins are known to exhibit different oligomeric states that can influence protein function and are closely linked to the phylogenetic origin of the protein^41–43^. In general, MRs are found to form dimers, trimers, pentamers, and, on some occasions, hexamers. While the crystal structure of MRs often reflects their oligomeric state in native membranes, in many cases, crystal packing can alter contacts between monomers, changing the protein arrangement in the membrane^44^. OLPVR1 forms a very loose dimer in the crystal structure, which is unlikely to reflect an oligomeric state of the protein correctly^7^. OLPVRII, being a close homolog of OLPVR1 from group 2 of viral rhodopsins, forms a highly stable pentamer^45^. The individual protomers of OLPVR1 and OLPVRII align with a high RMSD score (1.6 Å), whereas OLPVR1 does not align well with rhodopsins that are organized as dimers, such as CrChR2, yielding a lower RMSD score (6.3 Å) (Supplementary Fig. 13). To investigate the oligomeric state of OLPVR1 under physiological conditions, we reconstituted the protein into a lipid bilayer and performed high-speed atomic force microscopy (HS-AFM). The HS-AFM images revealed that OLPVR1 forms a dimer in the membrane. Using a combination of model building and molecular modeling, we computed a dimer model of OLPVR1^46^, consisting of two individual protomers connected by the TM3 and TM4 helices, similar to the CrChR2 dimer. Next, we assessed the stability of the monomer and dimer models in the different lipid environments (Supplementary Figs. 14 and 15). We observed that both monomer and dimer models remain stable over a 1 μs simulation and do not exhibit significant tilting, indicating that the monomer model of OLPVR1 can serve as a representative model for ion translocation modeling.

### Calcium binding in OLPVR1 causes partial destabilization of retinal

Unlike other calcium-binding MRs^47^, the OLPVR1 absorption spectrum maximum is insensitive to calcium under physiological conditions (Supplementary Fig. 16a), further indicating that calcium ions do not bind in the vicinity of the RSB or its counterions (D76 and D200). However, calcium ions influence the pH equilibrium of retinal deprotonation at alkaline pH (>pH 9.5). In the absence of calcium ions, the RSB deprotonation occurs with a p*K*_a_ of 10.6, whereas under the calcium-saturated conditions (100 mM CaCl_2_), the p*K*_a_ value is lowered (p*K*_a_ = 9.5). The calcium-binding concentration dependence was determined by measuring the evolution of the OLPVR1 absorption spectrum at pH 10.2. The absorption spectra difference follows a sigmoid curve depending on the inflection point at 17.0 ± 0.6 mM CaCl_2_ (Supplementary Fig. 16b). The influence of calcium binding on the RSB destabilization suggests that calcium binding occurs in the vicinity of the retinal chromophore but not near the RSB itself. To further evaluate the influence of calcium on OLPVR1 stability, we measured the thermal unfolding curves of OLPVR1 under different calcium concentrations (Supplementary Fig. 16c). In the absence of calcium, thermal denaturation occurred at Tm = 81.6 ± 0.3 °C. Under calcium-saturated conditions (100 mM CaCl_2_), the Tm was 77.2 ± 0.8 °C. The dependence of T_m_ on the concentration of calcium was surprisingly similar to the pH dependence, following a similar sigmoid dependence (Supplementary Fig. 16d) with the inflection point p*K*_a_ = 28.9 ± 3.4 mM CaCl_2_. While these values correlate well with the non-specific calcium-binding constant, we believe that both effects are likely due to specific calcium binding at site A, which is in direct proximity to the retinal cofactor. A possible explanation for this is the difference in lipid environment, as retinal destabilization experiments were conducted with OLPVR1 solubilized in n-dodecyl-β-D-maltoside (DDM). In contrast, the ATR-FTIR experiments were performed with OLPVR1 reconstituted in liposomes. It has to be noted that the reported destabilization of the retinal is reversible, not associated with any helical motion, and is less pronounced on thermal stability than a previously reported Zn^2+^ binding to a heliorhodopsin^48^.

## Discussion

### Molecular model of OLPVR1 inhibition by calcium ions

**Fig. 6:**
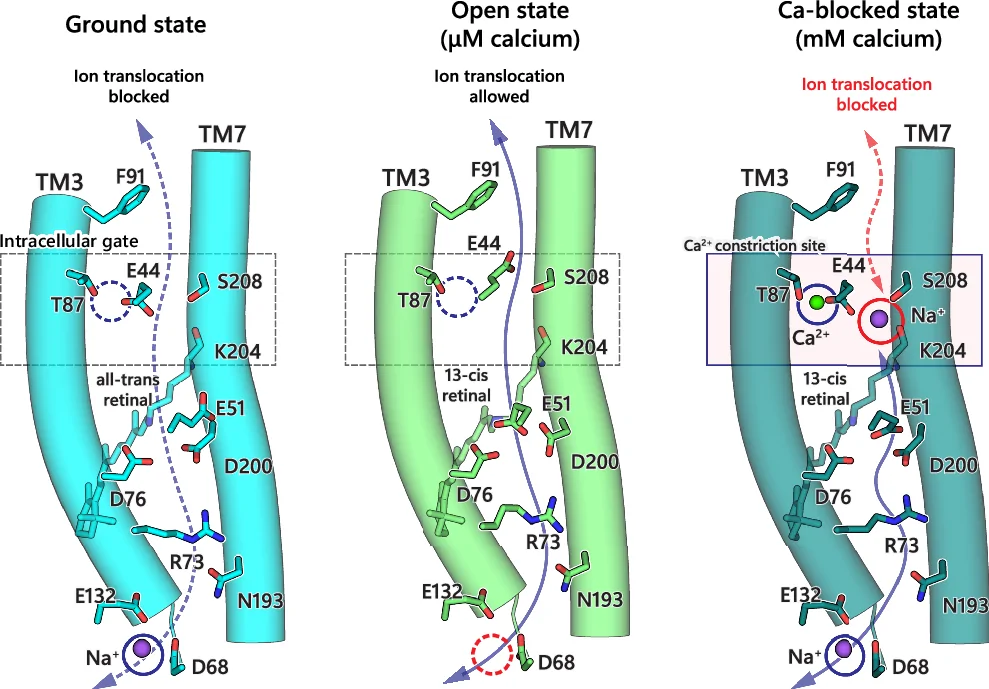
Molecular mechanism of VCR1s channeling inhibition by calcium. A simplified model of VCR1 ion translocation inhibition is illustrated using the atomic-resolution structures of OLPVR1 in its ground, open, and Ca-blocked states, presented in the left, middle, and right subplots, respectively. The conformations of the key residues are shown with sticks, and the positions of the sodium and calcium ions are highlighted. The composition of the Ca^2+^ constriction site is indicated with the red box, and the proposed ion translocation pathway is drawn with an arrow line.

The results presented in this and previous studies^7–9^ provide a comprehensive picture of OLPVR1 function under different Na^+^ and Ca^2+^ conditions and across all pH values. Based on our structural data, we propose a simplified model of calcium’s influence on OLPVR1 function, summarized in Figs. 1 and 6.

Under micromolar concentrations of calcium (<1 mM Ca^2+^), OLPVR1 functions as a cation-selective channelrhodopsin that is capable of transporting both monovalent^7^ (H^+^, Na^+^, and K^+^) and divalent^8^ (Ca^2+^) ions. When the concentration of calcium rises above a certain threshold (~2 mM), the calcium binds to the interior of the protein. This is consistent with the FTIR observation. Subsequently, it suppresses the opening of the intracellular gate upon light activation of the protein. Sodium binding affects the OLPVR1 photocycle length and the formation of the open state in solution (P4–P6 state), which is consistent with previously reported results on chloride binding for halorhodopsins^20,22^ and sodium binding by sodium pumps^16,49^. The interaction of OLPVR1 with calcium is significantly more pronounced. As we demonstrated in this study, when the calcium concentration reaches the critical threshold, OLPVR1 binds calcium at site A (between the side chains of T87 and T88), thereby affecting the interaction between the side chains of E44 and T87. Upon photoactivation, the side chain of E44 remains in a ground-state-like conformation and undergoes a change in protonation state, becoming deprotonated in the later stages of the photocycle. FTIR results showed that E51 also plays an important role in calcium binding to E44. This change in intracellular gate composition creates a transient sodium-binding site, formed by the side chains of E44 and S208 and the carboxyl oxygen of the retinal-binding K204 residue (Figs. 2 and 5). Transient sodium binding consequently causes slight rotation and partial destabilization of the retinal and, as a result, changes in the conformations of key gating residues, such as E51, T70, R73, D76, N193, and D200. Furthermore, calcium binding alters the OLPVR1 photocycle, causing both elongation of the photocycle and impaired formation of the two photointermediates (P_3_ and P_4_ states).

### Calcium inhibition may be a universal feature of viral channelrhodopsins

Microbial rhodopsins encoded by nucleoplasmic large DNA viruses (NCLDV) comprise two large subfamilies, VCR1 (also known as VR1) and VR2, both of which are NCLDV-specific^50^ (Supplementary Fig. 17). Despite their phylogenetic proximity, the VR2 family has a highly conserved pentameric fold that is hypothesized to facilitate ion translocation^45^. In contrast, the VCR1 rhodopsins exist as dimers in the membrane (Supplementary Note, Supplementary Fig. 13). Recent progress in the search for new NCLDVs has led to the identification of dozens of additional viral families and a significant increase in the number of NCLDV-associated genes^51^. Interestingly, most identified viruses encode VCR1 genes, suggesting that their function is likely similar over a wide range of infectable hosts^52^. We have previously reported that the VCR1 family has a core of highly conserved residues, including E44, E51, R73, D76, D200, and K204, which play a crucial role in their ion-channeling function^7,9^. In this work, we demonstrate that the same set of conserved residues mediates VCR1 calcium inhibition, thereby linking this distinct calcium interaction to its light-gated ion-channel function. Recently, we have also reported that another member of the VCR1 family, VirChR1, is capable of binding calcium ions during its photoreaction, further supporting the possibility that one of the primary functions of VCR1 is linked to its interaction with calcium^30^.

### OLPVR1 structures are consistent with a single-file-like ion mechanism

Understanding ion selectivity and permeation in channelrhodopsins, particularly for K⁺ and Na⁺, necessitates not only atomic-resolution structures but also direct visualization of ions within the conduction pathway. The presence of even a single bound ion can significantly influence local protein conformation, and the sufficiently high conductance observed in channelrhodopsins implies that multiple ions can occupy the pore simultaneously. Hodgkin and Keynes first proposed the concept of single-file ion movement in potassium channels, in which ions were shown to interact as they traverse the membrane^23^. This idea was later confirmed by structural studies and mechanistic models, revealing that ion–ion interactions within a narrow pore underlie selectivity and conductance^24–26^.

In the case of OLPVR1, one Na^+^ ion is bound in the ground state, no Na^+^ ions are observed in the open state, and two different Na^+^ ions are present in the Ca-blocked state (Fig. 1). In the Ca-blocked state, the interaction between Ca^2+^ and the side chain of E44 creates a local constriction site, causing the binding of a second Na^+^ ion. The presence of multiple Na⁺ ions inside the channel suggests possible ion–ion interactions between different Na^+^ ions within the channel. Notably, the persistent Na⁺ ion at the channel entry may facilitate translocation of ions already within the pore, a behavior reminiscent of the push–pull dynamics observed in classical potassium channels^53^. This interaction could contribute to increased conductance via a mechanism analogous, though not necessarily identical, to single-file permeation in potassium channels^25^.

While our atomic-resolution structures provide direct evidence for discrete Na⁺ occupancy at the extracellular entry site and, under Ca²⁺-blocked conditions, at the cytoplasmic constriction, we note important limitations of crystallography in describing the full permeation cycle. Na⁺ ions in a hydrated, polar pore are expected to exhibit short residence times and partial hydration, which may result in low site occupancies and elevated apparent mobility, particularly in photoactivated intermediates that are only fractionally populated. Consequently, additional transient binding positions along the conductive pathway may remain structurally unresolved. Our data are therefore consistent with a “single-file–like” permeation regime; however, alternative scenarios cannot be excluded. Discriminating between these mechanisms will require complementary functional and spectroscopic experiments under systematic ion substitution, targeted mutagenesis of pore-lining residues, and time-resolved structural approaches.

### The cellular function of viral channelrhodopsins is still a puzzle

VCR1s have strong preferential localization in the internal membranes, specifically in the endoplasmic reticulum (ER)^8^. Unlike other characterized channelrhodopsins, which prefer to reside in a plasma membrane environment^8^, VCR1s may exhibit biased localization due to their overall low TM helix length and the unusual composition of the TM4 helix, which has an additional juxtamembrane domain (Supplementary Note, Supplementary Fig. 18). The existence of structurally embedded factors that determine membrane localization strongly suggests that the cellular function of VCR1 rhodopsins is directly linked not only to calcium but also to the internal membranes, and specifically to the ER membranes^8^.

As we demonstrate in this work, calcium inhibition of VCR1s occurs at millimolar calcium concentrations, which would impair their function if expressed in outer membranes^54^. However, the intracellular Ca^2+^ concentrations range from 10^−7^ to 10^−4 ^M, which is insufficient to inhibit channels and thus allows Ca^2+^ transport. Since many microbes infected by giant viruses lack eyespot-like organelles, it is unlikely that VCR1 rhodopsins can mediate complex phototaxis comparable to that of ChR1 and ChR2. Instead, the expression of VCR1s in the ER membranes of infected cells would trigger Ca^2+^ release upon a high-light signal, identical to the effect observed in oocytes and HEK cells^8^. In mature cells, Ca^2+^ release is associated with various stress stimuli^55–58^, including high-light conditions^59^. We cannot rule out other potential roles for VCR1s, and therefore, it remains a question that warrants further study.

## Methods

### Cloning, expression, purification, and crystallization

The protein production was performed by optimizing previously described protocols for these samples^7^. In brief, the *E. coli* cells of strain C41 (StabyCodon T7, Eurogentec, Belgium) were transformed with the modified OLPVR1-His8 and BRIL-VirChR1-His8 in the modified pET15b expression plasmid^60^. The constructs are available in Supplementary Data 1. Transformed cells were grown in shaking baffled flasks in an autoinducing medium ZYP-5052 containing 100 mg/L kanamycin at 37 °C. When the OD600 in the growing bacterial culture is 0.8–1.0 (glucose level <10 mg/L), 10 μM all-trans-retinal (Sigma-Aldrich), and 1 mM isopropyl β-d-1-thiogalactopyranoside were added, the incubation temperature was reduced to 20 °C and incubated for 18 h. After incubation, cells were collected by centrifugation (5000×*g*, 30 min) and disrupted in an M-110 P Lab Homogenizer (Microfluidics) at 20,000 p.s.i. in a buffer containing 20 mM Tris-HCl, pH 8.0, with 50 mg/L DNase I (Sigma-Aldrich). The membrane fraction of the cell lysate was isolated by ultracentrifugation at 90,000×*g* for 1 h at 4 °C (Type 70 Ti Fixed-Angle Titanium Rotor, Beckmann). The pellet was resuspended in a buffer containing 20 mM Tris, pH 8.0, 0.1 M NaCl, and 1% n-dodecyl β-D-maltoside (DDM, Anatrace, Affymetrix) and stirred for 18 h for solubilization. The insoluble fraction was removed by ultracentrifugation at 90,000×*g* for 1 h at 4 °C. The supernatant was loaded on a Ni-NTA column (Qiagen) and washed with a buffer containing 20 mM Tris, 150 mM NaCl, 30 mM imidazole, and 0.05% DDM buffer (pH 8.0). The protein was eluted in a buffer containing 20 mM Tris, 150 mM NaCl, 300 mM imidazole, and 0.05% DDM (pH 8.0). The eluate was subjected to size-exclusion chromatography on a 20 ml Superdex 200i 10/300 GL column (GE Healthcare Life Sciences) in a buffer containing 10 mM Tris, pH 8.0, 150 mM NaCl, and 0.05% DDM. Protein-containing fractions with an A280/A500 absorbance ratio (peak ratio, p.r.) of lower than 1.5 were pooled and dialyzed against 100 volumes of 20 mM Tris, 150 mM NaCl, and 0.05% DDM (pH 8.0) buffer twice for 2 h to dispose of imidazole. The purified protein was concentrated to 40 mg/ml for crystallization.

The crystals of OLPVR1 were grown using the in meso approach described in our previous works^7,12,17,18,44,61^. In particular, the solubilized protein (20–40 mg/ml) was mixed with the premelted at 42 °C monopalmitolein (MP, Nu-Chek Prep) in a 2:3 ratio (protein:lipid) to form a lipidic mesophase. The mesophase was homogenized using coupled syringes (Hamilton) by transferring it from one syringe to another until a homogeneous, gel-like material was formed. NT8 crystallization robot (Formulatrix) dispensed 150 nL of the mesophase and 400 nL of the precipitant solution on a 96-well LCP glass sandwich plate (Marienfeld). The crystals were grown at 20 °C and appeared in 1 to 3 weeks. The best crystals were obtained at a protein concentration of 30 mg/ml in 100 mM Tris, pH 8.0, 900 mM NaCl, and 24% (w/v) PEG 6000.

### Determination of the crystal structures of OLPVR1 with calcium and barium ions bound to the surface of the channel

Once the crystals reached their final size, the crystallization wells were opened and covered with 100 μL of harvesting solution, which consisted of crystallization buffer and either 100 mM calcium chloride (resulting in Ca-surface crystals) or 100 mM barium chloride (resulting in Ba-surface crystals). Crystals were harvested using the Dual Thickness MicroMounts (MiteGen, USA) after 24 hours of soaking in the dark and were immediately flash-cooled in liquid nitrogen (LN2).

Diffraction data from the Ca-surface and Ba-surface crystals were collected at ID23-1 (ESRF, Grenoble, France). The data collection protocol was optimized with BEST^62^. Initially, data from both complexes were collected at a wavelength of 1.7 Å (7.293 keV). On the one hand, this gives a reasonably high resolution at the edges of the detector; on the other, close to the K edge of calcium (4.038 keV; f″ =  1.5 e^−^ at the chosen wavelength) and the L-I edge of barium (5.989 keV; f″ = 9.3 e^−^ at the selected wavelength). While the Ba-surface crystals yielded a good anomalous signal, the Ca-surface crystals did not produce a significant anomalous signal. Therefore, the final set with calcium was taken at a wavelength of 0.8856 Å (14 keV) to maximize the resolution.

Diffraction images were integrated using XDS^63^. In the case of a Ca-surface structure, the reflection intensities were anisotropically scaled using the Staraniso web service, with the cutoff criteria being local(I/sigI) = 1.2. For a Ba-surface structure, the reflection intensities were scaled using XSCALE, with cutoff criteria of CC1/2 = 16% and Friedel’s law assumed not to be satisfied. The data collection statistics are shown in Table 1.

**Table 1:** Data collection and refinement statistics

	Ca-surface	Ba-surface	Ca-inner	Ca-blocked
**Data collection**				
Space group	P 2_1_ 2_1_ 2	P 2_1_ 2_1_ 2	P 2_1_ 2_1_ 2	P 2_1_ 2_1_ 2
Cell dimensions				
*a*, *b*, *c* (Å)	46.45, 115.36, 53.50	46.22, 115.03, 53.29	46.39, 115.45, 53.42	46.18, 115.65, 53.49
*α*, *β*, *γ* (°)	90, 90, 90	90, 90, 90	90, 90, 90	90, 90, 90
Resolution (Å)	36.18 – 1.45 (1.62 – 1.45) *	26.65 – 2.10 (2.15 – 2.10)	43.05 – 1.41 (1.46 – 1.41)	39.27 – 1.52 (1.62 – 1.52)
*R*_pim_	0.029 (0.449)	0.083 (1.207)	0.016 (0.919)	0.023 (1.207)
*CC*_1/2_	0.998 (0.645)	0.997 (0.177)	0.999 (0.272)	1.000 (0.241)
*I* / s*I*	11.6 (1.6)	6.7 (0.7)	19.0 (0.8)	14.7 (0.6)
Completeness spherical (%)	65.9 (11.9)	100 (99.9)	90.9 (43.4)	75.9 (22.0)
Completeness ellipsoidal (%)	86.4 (52.5)	N/A	95.2 (66.0)	93.2 (63.0)
Redundancy	4.8 (4.5)	6.1 (4.7)	13.0 (9.2)	4.3 (3.9)
SigAno	N/A	1.097/0.947**	N/A	N/A
Anomal Corr	N/A	27/17**	N/A	N/A
**Refinement**				
Resolution (Å)	35.08 – 1.45	26.65 – 2.10	35.08 – 1.45	39.27 – 1.52
No. reflections work / free	33960/1684	17216/886	33961/1684	34118/1692
*R*_work_ / *R*_free_	0.1689/0.1976	0.2073/0.2489	0.1858/0.2064	0.1949/0.2259
No. atoms	2545	2233	2477	2800
Protein	2112	1952	2048	2430
Retinal	40	20	40	40
Lipids	260	184	261	223
Solvent	133	77	128	107
*B*-factors	27.92	45.34	28.65	34.06
Protein	24.66	43.50	25.41	31.88
Retinal	18.12	35.22	18.16	26.81
Lipids	50.17	64.01	51.43	55.31
Solvent	39.05	50.07	37.41	41.87
R.m.s. deviations				
Bond lengths (Å)	0.006	0.003	0.010	0.007
Bond angles (°)	0.77	0.53	1.09	0.88
Ramachandran favored (%)	99.54	99.54	99.07	99.08
Ramachandran outliers (%)	0	0	0	0
Rotamer outliers (%)	0.87	1.87	0.89	1.13
Clashscore	4.76	3.11	4.25	5.69

*Values in parentheses are for the highest resolution shell

**Values are for the lowest and for the 4.70−4.20 Å resolution shells

Polyala model of the previously published OLPVR1 structure (PDB ID: 9IN7^9^) was used as a search model for molecular replacement in Phaser^64^ from Phenix^65^. The resultant structure (P2_1_2_1_2 space group with 1 molecule per asymmetric unit, ASU) was rebuilt in Phenix.Autobuild^66^. Several cycles of interactive refinement in Coot^67^ and anisotropic B-factor refinement in Phenix.Refine^68^ led to the final model, the quality of which was assessed with Phenix. The cavities were calculated using HOLLOW^69^. Hydrophobic-hydrophilic boundaries of the membrane were computed using a PPM server^70^. The refinement statistics are presented in Table 1.

Calcium binding was detected as a 10.8 σ-level peak on the F_O_^Ca-surface^-F_O_^Ca-free^ difference map, where F_O_^Ca-surface^ were the structural factors of the Ca-surface crystals, while F_O_^Ca-free^ were the structural factors of the previously published calcium-free OLPVR1 ground state structure (PDB ID: 9IN7^9^). The binding was then confirmed with the CheckMyMetal web service^29^. Barium binding was detected at the same site as a 5.9 σ-level peak on the anomalous maps built in Phenix.Refine^68^. Estimated occupancies of Ca^2+^ and Ba^2+^ cations are 0.5 and 0.3, respectively.

### Determination of the crystal structure of OLPVR1 with a calcium ion bound to the inner part of the channel

To allow calcium and barium ions to enter the channel (cation-inner state), the protocol from the previous chapter was modified. To obtain Ca-inner and Ba-inner states, we soaked the crystals in the crystallization buffer containing 10 mM CaCl_2_ (we reduced the salt concentration because Ca-inner crystals are highly sensitive to laser activation at high salt concentrations; see the next chapter) or 100 mM BaCl_2_, respectively. The soaking was also done for 24 h, but now under constant illumination by the dim light of the microscope lamp. After 24 h of soaking under light, the lamp was turned off for 30 min. The crystals were then harvested and frozen in liquid nitrogen.

The diffraction data from the Ca-inner state crystals were collected at the P14 EMBL beamline of the PETRAIII synchrotron source. Initially, we also tried to collect anomalous data, but due to the absence of a strong anomalous signal, the final dataset was collected with a wavelength of 0.9755 Å (12.71 keV) to maximize resolution. Diffraction images were integrated using XDS^63^. The reflection intensities were anisotropically scaled using the Staraniso web service, with the cutoff criteria being local(I/sigI) = 0.5. The model of the Ca-inner state was built as described in “Determination of the crystal structures of OLPVR1 with calcium and barium ions bound to the surface of the channel”. The data collection and refinement statistics are shown in Table 1.

All our attempts to obtain OLPVR1 with Ba^2+^ ions bound to the inner part of the channel failed–no significant densities were detected on the anomalous nor on the F_O_^Ba-inner^-F_O_^Ba-free^ difference map inside the channel, where F_O_^Ba-inner^ were the structural factors of OLPVR1 soaked in barium chloride under microscope lamp light, while F_O_^Ba-free^ were the structural factors of the previously published barium-free OLPVR1 structure (PDB ID: 9IN7). In contrast F_O_^Ca-inner^-F_O_^Ca-free^ difference map, where F_O_^Ca-inner^ were the structural factors of OLPVR1 soaked in calcium chloride under microscope lamp light, while F_O_^Ca-free^ were the structural factors of the previously published calcium-free OLPVR1 structure (PDB ID: 9IN7^9^), revealed an 18.1 σ-level peak near T87 and T88 residues. Moreover, only the alternative conformation of these residues that supported calcium coordination were preserved, compared to multiple conformations present in this region in the Ca-free structure (PDB ID: 9IN7). This indicated that calcium is bound to the cytoplasmic part of the channel. The estimated occupancy of calcium in the inner binding site is 0.4. Interestingly, no calcium binding was detected at the surface. Importantly, this occurred only when crystals were soaked under constant illumination of the microscope light.

As the observed coordination of the calcium cation was unusual according to the CheckMyMetal web service^29^, we also performed MD studies to confirm that Ca^2+^ is stable in this binding site, which are described in “Molecular dynamics simulations”.

### Determination of the crystal structure of OLPVR1 in the calcium-blocked state

To reveal the structural basis for calcium-dependent inhibition of OLPVR1 channeling, we performed cryotrapping with the Ca-inner state crystals, obtained as described in ‘Determination of the crystal structure of OLPVR1 with calcium ion bound to the inner part of the channel’. Accumulation and determination of the calcium-blocked structure was conducted using crystal cryotrapping. UV-visible absorption spectra were measured at the *ic*OS laboratory at the ESRF (Grenoble, France)^71^, using a DH-200-BAL deuterium-halogen lamp (Ocean Optics, Dunedin, FL) as a reference light and a QE65 Pro spectrometer (Ocean Optics, Dunedin, FL). The crystal was placed between two objectives under the 100 K cryostream (Oxford). The lamp was connected to one of the objectives through a 200-µm-diameter fiber, resulting in the 50 µm focal spot on the crystal. The spectrometer was connected to the opposite objective with a 400-µm-diameter fiber. The spectra were recorded with 100 ms acquisition time and averaged 20 times. For the crystal activation, a 532 nm laser (CNI Laser, Changchun, P.R. China) was connected through the shutter to the third objective, whose optical axis is perpendicular to the lamp and spectrometer objectives. The laser was connected through a 600-µm-diameter fiber, resulting in the 150-µm-diameter laser spots on the crystal. The mean size of the crystals was 150 × 75 × 20 μm. During the cryotrapping, the plate-like crystals were oriented so that the largest plane (150 ×  75 μm) was perpendicular to the laser beam. Before the cryotrapping experiments, we first determined the absorption spectrum of OLPVR1 in crystals (Supplementary Fig. 9).

The accumulation of the Ca-blocked state was performed as follows. First, the crystal was placed into a cryostream at 100 K. Following the recording of the ground spectrum, the crystal was illuminated by the laser together with the simultaneous blocking of the cryostream for 1–5 s, allowing the crystal to heat up to 293 K. After the time delay, the cryostream was unblocked, and the laser was switched off once the crystal went back to 100 K. The resulting red-shifted spectrum of the crystal indicated the partial transition of OLPVR1 to the Ca-blocked (P_blocked_) state (Supplementary Fig. 9). Notably, the red shift induced by a green laser (532 nm) was much more significant for Ca-inner crystals than for Ca-free crystals that we used in our previous work to obtain the open state structure, so we did not need to switch to using a blue laser (473 nm) to accumulate a sufficient fraction of the Ca-blocked state. The highest occupancy of the Ca-blocked state was achieved when crystals were illuminated for 3 seconds with a 532 nm laser at a power density of 2 mW/cm². The estimated occupancy of the P_blocked_ intermediate from the 5-Gaussian fit was 27 ± 8%. In our occupancy analysis, we excluded the Gaussians at 280 and 439 nm because their amplitudes remained constant under laser illumination. To check if the Ca^2+^ blockage is reversible, the third spectrum was recorded after blocking the cryostream in the dark for 2 s. We found that the crystals partially return to the ground state; however, the majority of the protein (21 ± 7%) remains in the Ca-blocked state, thereby further distinguishing it from the open state in the absence of calcium.

Initially, we performed cryotrapping with OLPVR1 crystals soaked in a crystallization buffer supplemented with 10, 20, 50, and 100 mM CaCl_2_. However, when the concentration of CaCl_2_ was 20 mM or higher, the cryotrapping resulted in the transparent crystals (Supplementary Fig. 9e, f). The reaction was irreversible, resulting in the complete loss of diffraction. This indicates that the high concentration of calcium was harmful for OLPVR1, likely resulting in the loss of the retinal upon light illumination. Thus, for our subsequent experiments, the concentration of calcium chloride was chosen as 10 mM.

A similar cryotrapping setup was established at the P14 EMBL beamline of the PETRAIII synchrotron source (Hamburg, Germany) for the X-ray diffraction data collection from the Ca-blocked crystals. The Ca-blocked state structure was built using extrapolated maps contoured with Xtrapol8^38^. The estimated occupancy of the Ca-blocked state (using the difference-map method in Xtrapol8) was 30%, which is in good agreement with the spectral data. To construct the Ca-blocked state, we also used k-weighted TV-denoised isomorphous difference maps built with Meteor^72^ (*phaseboost* protocol with default parameters). Examples of the TV-denoised difference maps can be found in Supplementary Fig. 19. Finally, the two structures (Ca-inner state and Ca-blocked states) were merged into one (with occupancies of 70 and 30%, respectively) for the final refinement in Phenix.Refine.

F_O_^Ca-blocked^-F_O_^Ca-inner^ difference map (where F_O_^Ca-blocked^ were the structural factors of the cryotrapped Ca-blocked state, while F_O_^Ca-inner^ were the structural factors of OLPVR1 soaked in calcium chloride under microscope lamp light) revealed a 6.8 σ-level peak near the carbonyl group of the retinal-carrier lysine residue. Importantly, no negative peaks were detected near the sodium-binding site at the extracellular part of the channel and near the calcium binding site near T87 and T88 residues. This complicated the identification of the origin of the observed positive peak, so we performed MD simulations that are described in “Molecular dynamics simulations”.

### Molecular dynamics simulations

OLPVR1 in the ground (non-illuminated and illuminated) and the Ca-blocked states were simulated in the monomeric form. Rectangular unit cells contained 205 POPC molecules, 14350 water molecules, 0.1 M NaCl, 0.01 M CaCl_2_ ions for the monomer in the calcium-bound illuminated ground state (PDB ID 9SOF) and calcium-blocked state (PDB ID 9SOG); 1000 lipid molecules (10 POPA, 30 SLPI, 30 SAPI, 30 OLPS, 40 PSM, 50 POPE, 50 CHOL, 70 SAPE, 80 PSPE, 160 POPC, 180 PLPC, 270 SAPC), 48710 water molecules, 0.15 M NaCl and 1035 lipid molecules (5 POPA, 20 GLPA, 25 POPI, 55 PAPS, 75 PAPE, 80 SSM, 80 NSM, 85 POPE, 115 POPC, 165 PLPC, 330 CHOL), 45732 water molecules, 0.15 M NaCl for the monomer in the non-illuminated ground state (PDB ID 9IN7) in ER and plasma membranes^73^, respectively.

OLPVR1 in the non-illuminated ground state was also simulated in the dimeric form (starting model obtained using homology modeling). Rectangular unit cells contained the same mixture of lipids as for the monomer, 48242 water molecules, 0.15 M NaCl and the same mix of lipids as for the monomer, 47,193 water molecules, 0.15 M NaCl for the dimer in ER and plasma membranes, respectively. All atomistic systems were prepared using the Membrane Builder tool^74^ of the CHARMM-GUI web server^75^. Starting structures included water molecules, internally bound Na^+^ and Ca^2+^ ions, and fatty acids (near the pore), as observed in the X-ray structures. Positions of other ions were generated using the Monte-Carlo method^76^. Protonation states of titratable amino acid residues, excluding E44 and E51, were assigned using PROPKA3^77,78^. E44 was deprotonated, and E51 was protonated in the illuminated ground and the Ca-blocked states; E44 and E51 were protonated in the non-illuminated ground state; the Schiff base was protonated. We analyzed planarity and bond length alternation as defined in ref. ^79^, and observed that the retinal remained in the 13-cis conformation in the Ca-blocked state simulations. (Supplementary Fig. 20).

MD simulations were conducted using GROMACS 2022^80^ and the CHARMM36m force field^81,82^, TIP3P water model^83^, and multi-site Ca^2+^ model^84^. The parameters for retinal bound to lysine were adapted from ref.^85^. Systems were energy minimized using the steepest descent method, thermalized, and simulated for 500 ns for systems containing a POPC bilayer and for 1000 ns for systems containing ER and plasma bilayers using the leapfrog integrator with a time step of 2 fs, at a reference temperature of 303 and 310 K, respectively, and at a reference pressure of 1 bar. Backbone atoms were harmonically restrained to their experimentally determined positions with a constant of 1000 kJ mol^−1^ nm^−2^ throughout the simulations of the systems containing a POPC bilayer. Temperature was coupled using Nosé-Hoover thermostat^86^ with a coupling constant of 1 ps^−1^. Pressure was coupled with a semi-isotropic stochastic cell rescaling barostat^87^, with a relaxation time of 5 ps and a compressibility of 4.5 · 10^−5^ bar^−1^.

The simulations were performed using periodic boundary conditions. The center of mass of the reference structure was scaled using the pressure-coupling scaling matrix. The covalent bonds to hydrogens were constrained using the LINear constraint solver (LINCS) algorithm^88^. The nonbonded pair list was updated every 20 steps with a cutoff of 1.2 nm. Force-based switching function with a switching range of 1.0–1.2 nm and the particle mesh Ewald method with a 0.12 nm Fourier grid spacing and a 1.2 nm cutoff were used to treat van der Waals and electrostatic interactions. Average positions of the membrane boundaries and thickness values were calculated using g_lomepro, version 1.0.2^89^.

### High-speed AFM measurements

The reconstitution of OLPVR1 into a lipid bilayer was achieved using the nanodisc method with slight modifications^43^ based on the manufacturer’s protocol (Sigma‒Aldrich, USA). We used a lipid mixture with a 3:1 ratio of POPE to POPG. The lipid mixture (120 μg) was dissolved in chloroform and evaporated under a stream of N2 gas to completely remove the solvent. The lipid mixture was then suspended in 50 μl of buffer containing 20 mM Hepes-KOH (pH 7.4), 100 mM NaCl, and 4% DDM, followed by sonication for approximately 1 min using a tip sonicator (TAITEC). The dissolved OLPVR1 (1 nmol) and membrane scaffold proteins (50 μl, 1 mg/mL final concentration) (MSP1E3D1, Sigma-Aldrich, M7074) were added to the lipid suspension and mixed for approximately 1 h with rotation in the dark at 4 °C. Finally, 60 mg of Bio-Beads SM-2 (Bio-Rad, Hercules, 1523920) were added. The samples were dialyzed overnight at 4 °C to remove the detergent. According to the manufacturer’s protocol, the nanodisc samples were typically fractionated on a column for size purification (~10 nm in diameter); however, we did not purify the reconstituted samples, and as a result, we obtained flat membranes with limited sizes, typically less than 30 nm in diameter.

For this study, a laboratory-built HS-AFM was employed^43,90^. In this apparatus, the cantilever deflection is measured using the optical beam deflection method. A 780 nm laser beam is directed onto the backside of a cantilever with a gold film by using an objective lens (Nikon, CFI S Plan Fluor ELWD 60×). The incident and reflected laser beams are then separated via a polarized beam splitter. The cantilever (Olympus, BL-AC7DS-KU4) dimensions are 6–7-μm in length, 2 μm in width, and 90 nm in thickness, with a spring constant, a resonant frequency, and a quality factor of ~0.2 N/m, 400 kHz, and 2, respectively, in liquid. The rate of data collection is 2.0 frames per second. The free oscillation amplitude was maintained at about 1 nm, and the set-point amplitude for the feedback control was set to ~90% of the free amplitude during HS-AFM scanning. An amorphous carbon tip of roughly 100 nm length was fabricated on the original bird’s beak tip by electron beam deposition. It was sharpened to a radius of approximately 2 nm by plasma etching in an argon atmosphere to create a sharp probe. To reduce the interaction force between the sample and the AFM tip, HS-AFM was operated in “only trace imaging” (OTI) mode^91^. Operation and data collection for HS-AFM were performed using laboratory-developed software written in Visual Basic. NET (Microsoft). All HS-AFM experiments were performed at room temperature (24–26 °C) and were independently repeated at least three times, producing consistent results. Imaging was performed in a buffer solution containing 20 mM Tris-HCl (pH 8.0) and 100 mM KCl. The mica surface was treated for 3 min with 0.00005% (3-aminopropyl) triethoxysilane (APTES) (Sigma-Aldrich).

### ATR-FTIR spectroscopy

Ca^2+^ binding to OLPVR1 reconstituted into membranes was monitored by Attenuated Total Reflection (ATR)-FTIR spectroscopy in two experimental modes^34,35,47,48^. The first is that two buffers, A and B, were introduced alternately, and FTIR spectra were obtained from the difference between them under dark or light conditions (Supplementary Fig. 6a). The second is that samples were illuminated in a buffer, from which light-minus-dark difference FTIR spectra were obtained (Supplementary Fig. 6b). Purified samples in 0.05 % DDM were reconstituted with a protein-to-lipid (asolectin; Sigma-Aldrich) molar ratio of 1:5 (except for Fig. 4d) or 1:20 (Fig. 4d), by removing DDM with a buffer containing 20 mM Tris-HCl, 150 mM NaCl, pH 8 (except for Fig. 4d) and Bio-Beads SM-2 (Bio-Rad) at 4 °C in dark conditions. The OLPVR1 sample in asolectin liposomes was washed repeatedly with buffers containing 2 mM NaCl/NaH_2_PO_4_ (pH 7.5) (except for Fig. 4d) or 2 mM NaCl/KH_2_PO_4_ (pH 7.5) (Fig. 4d) and collected by ultracentrifuging for 20 min at 222,000×*g* at 4 °C in dark conditions. The lipid-reconstituted OLPVR1 was placed on the surface of a silicon ATR crystal (Smiths, with three internal total reflections) and allowed to dry naturally. The sample was then rehydrated with a buffer, and the physically adsorbed sample on an ATR crystal was stable. The temperature was maintained at 283 K by circulating water.

In the first experiment, two buffers, A and B, were introduced alternately via a flow system at a flow rate of 0.6 mL min^−1^ (Supplementary Fig. 6a). The perfusion buffer consists of 20 mM Tris-HCl, pH 8 (buffer A), and 20 mM Tris-HCl with various CaCl2 concentrations (buffer B), pH 8. ATR-FTIR spectra were recorded in kinetics mode at 2 cm^−1^ resolution using an FTIR spectrometer (Agilent) equipped with a liquid nitrogen-cooled mercury-cadmium-telluride (MCT) detector. An average of 1140 interferograms per 10 min was recorded first with buffer A, then with buffer B, and finally with buffer A. Each spectrum was measured when the baseline, the difference between two spectra, became minimal, which took 5 min for the addition of Ca^2+^ and more than 30 min for the removal of Ca^2+^ (Supplementary Fig. 6a). Dark and light spectra correspond to spectral acquisitions with and without illumination of the sample. A light source was installed above the ATR crystal, and a 501 nm interference filter and a condenser lens were placed directly beneath it. The difference spectra were calculated as the averaged spectra in buffer B minus buffer A. The absorption of unbound salt and water vapor was subtracted from the raw spectra to correct the spectra. The baseline drift caused by protein swelling/shrinkage was also subtracted to obtain the final spectra. This experiment was applied not only to the wild-type protein but also to the E44Q and E51Q mutant proteins, which were expressed in Sf9 cells as reported previously^37,92^.

In the second experiment, the flow system was not used. Instead, the sample on the ATR crystal was filled with a buffer in a pool (Supplementary Fig. 6b). Buffers were composed of 20 mM Bis-tris propane (BTP), pH 8; or 20 mM BTP, 100 mM NaCl, pH 8; or 20 mM Tris-HCl, 5 mM MgCl_2_, pH 8; or 20 mM Tris-HCl, 5 mM CaCl_2_, pH 8 (Fig. 4d); or 20 mM Tris-HCl, 5 mM CaCl_2_, pH 8 (Fig. 4e). The “dark” spectrum was obtained for 3 min in the dark. Then, the sample was illuminated in a buffer, and the “light” spectrum was obtained over 30 seconds. We calculated the difference to obtain the light-minus-dark spectrum, which was repeated 8–9 times (Fig. 4d) and 18 times (Fig. 4e). We found that the photocycle of OLPVR1 was longer in the presence of Ca^2+^ than in its absence, and thus, a 5-min longer interval was taken for the measurements in the presence of Ca^2+^ (Supplementary Fig. 6b). The light-minus-dark spectra were obtained in H_2_O and D_2_O.

### Thermal stability measurements

The thermal stability of OLPVR1 was investigated using the nano differential scanning fluorimetry (nanoDSF) method. The experiment was done using the Prometheus NT.48 (NanoTemper, Germany). No protein labeling was applied. The OLPVR1 concentration was adjusted to 1 mg/mL for all measurements. The OLPVR1 sample buffer was composed of 20 mM Tris, pH 8, 150 mM NaCl, and 0.1% (w/w) DDM, and was supplemented with X mM CaCl2, where X was varied from 0 to 200. The melting point (Tm) was calculated as the ratio of the fluorescent signals at 350 and 330 nm (F350/F330), using a sigmoid fit of ref. ^93^.

### Ultrafast UV/vis spectroscopy at neutral pH conditions

Ultrafast UV/vis spectroscopy was conducted using a home-built pump-probe setup described elsewhere^94^. In short, a Ti:Sa chirped pulse regenerative amplifier (MXR-CPA-iSeries, Clark-MXR Inc.) provided fs-laser pulses. The laser system was operated at a central output wavelength of 775 nm with a repetition rate of 1 kHz, yielding laser pulses with a width of 150 fs. The excitation pulses were generated in a two-stage NOPA setup, configured to produce pulses with a central wavelength of 510 nm. White light continuum probing pulses were generated by focusing the laser fundamental into a 5 mm CaF_2_-crystal. Subsequently, the generated white light was split into the reference and sample paths, with only the sample path guided through the sample of interest. Absorption signals were detected by a system composed of two spectrographs (Multimode, AMKO), equipped with a grating (500 nm blaze, 1200 grooves per mm), a photodiode array (S8865-64, Hamamatsu), and a driver circuit (C9118, Hamamatsu). The obtained signals were digitized using a 16-bit data acquisition card (NI-PCI-6110, National Instruments). Pump and probe pulses were set to the magic-angle condition to prevent any anisotropic effects. The cuvette was moved in a plane perpendicular to the laser beams to exchange the sample between laser shots and to prevent degradation. Samples were measured in a 1 mm quartz glass cuvette, and the optical density was adjusted to ~0.3 OD per mm.

### UV/vis pump-probe spectroscopy resolving the ns timescale at neutral pH conditions

To bridge the experimental time gap on the ns timescale, a home-built pump-probe setup consisting of two synchronized fs-laser systems (Spitfire Ace and Spitfire Pro, both from Spectra Physics) was used. The general design was loosely adapted from ref. ^95^. In this setup, the Spitfire Ace laser system was used to generate the pump pulse with a two-stage NOPA, and the Spitfire Pro laser system was used to generate the probe pulse by focusing the laser fundamental into a 3 mm CaF_2_ Crystal. To achieve synchronization, both lasers are connected via a Lok-to-Clock (Models 3930 and 3931, Spectra Physics) and a Digital Delay Generator (Model DLG645, Stanford Research Systems). Overall, the Spitfire Pro system consists of three different units. The Tsunami, which acts as the seed, is pumped by the Millenia, the pump laser called Empower, and the regenerative Amplifier Spitfire Pro, which also provides the name of the whole system. The Lok-to-Clock sets the pulse and the delay of the seed laser of the Spitfire Pro (Tsunami) via a signal from a photodiode from the seed laser of the Spitfire Ace (MaiTai). The frequency is set to 80 MHz, resulting in a maximum delay of 12.5 ns. Furthermore, the time delay generator (TDG) of the Spitfire Ace provides a signal to the Digital Delay Generator, which is passed to the TDG of the Spitfire Pro after a given delay time. Based on the 1 kHz repetition rate, this can be applied to achieve delay times of up to 1 ms. To properly set the time zero, first, the settings are identified for the TDG of the Spitfire Ace, the Lok-to-Clock, and the DLG645, which cause both pulses to hit the sample simultaneously. Afterward, a variable delay can be achieved by adjusting the delay times for the Lok-to-Clock and the DLG645, respectively. Absorption signals were detected using a spectrometer comprising a sensor (MMS UV-vis II, Carl Zeiss) and a preamplifier (Tec5). Protein concentration was adjusted to yield an optical density of 0.6 in a 1 mm quartz cuvette. Pump and probe beams were set to the magic-angle condition to minimize anisotropic effects. Furthermore, the cuvette was continuously moved to prevent sample degradation. The pump beam was adjusted to a central wavelength of 510 nm and a power of 200 nJ/pulse.

### Transient UV/vis flash photolysis of protein crystals

A home-built flash-photolysis setup was used to measure the in crystallo photocycle kinetics of OLPVR1 from 5 µs to 4.75 s. The excitation wavelength was set to 510 nm using an optical parametric oscillator (OPO, preciScan, GWU-Lasertechnik), pumped by an ns Nd:YAG laser (SpitLight 600, Innolas Laser). The average output energy was set to ~2.2 mJ/cm². The white light generated by a Xenon or Mercury-Xenon lamp (LC-08, Hamamatsu) was used to probe the absorption changes from 340 to 700 nm with a 20 nm step size. Therefore, the white light was guided through two identical monochromators. Each monochromator was equipped with two gratings (400 and 600 nm, both with 1200 L/mm blaze). The gratings were switched at 500 nm according to the blaze angle to achieve optimal white-light intensity. One monochromator was placed in front of the sample, and the other one was placed after the sample. The signals were detected with a photomultiplier tube (Photosensor H6780-02, Hamamatsu) and measured as electrical signals using two oscilloscopes (5244B/D, Pico Technology) that overlapped in time. Thirty acquisitions were measured and averaged for each probing wavelength to increase the signal-to-noise (S/N) ratio. The large raw data files were reduced afterward using forward averaging and a combined linear and logarithmic timescale.

The sample preparation of the crystal samples was performed similarly to refs. ^20,21^. A 50 µm Teflon spacer with a hole in the middle was placed on a CaF_2_ window. 2–10 μL of the LCP-crystal mixture was placed in the middle of the CaF_2_ window, and a second CaF_2_ window was carefully placed on top to create a homogenous sample spot in the middle of the windows. Vacuum grease was used to adhere the Teflon spacer to the CaF2 windows and to seal the cuvette properly, preventing dehydration of the crystals. The flash-photolysis data were subjected to the model-free lifetime distribution analysis (LDA) using OPTIMUS software^96^. The LDA yields the lifetime distributions of the individual photointermediate transitions, which are visualized in a lifetime density map (LDM).

### Transient UV/vis flash-photolysis spectroscopy at neutral pH conditions

The used home-built flash-photolysis setup consists of an ns Nd:YAG laser (SpitLight 600, Innolas Laser) and an optical parametric oscillator (preciScan, GWU-Lasertechnik). The OPO output wavelength was set to 510 nm, and the output energy was adjusted to ~2.2 mJ m^−2^. Absorption changes were probed using white light provided from either a Xenon or a Mercury-Xenon lamp (LC-08, Hamamatsu). To select the individual probing wavelengths, white light was guided through two identical monochromators (500 nm blaze, 1200 lines per mm), with the first placed in front of the sample and the second after the sample. The detection system used a photomultiplier tube (H6780-2, Hamamatsu), and the signal was subsequently converted into an electrical signal. The measured signal was ultimately recorded using two oscilloscopes (PicoScope 5244B/D, Pico Technology), each set to cover overlapping timescales. For each probing wavelength, 30 acquisitions were measured and averaged to optimize the S/N ratio. The large raw data files were subjected to data reduction using a combined linear and logarithmic timescale to obtain files of reasonable size for further analysis. All samples were measured in a 2 × 10 mm quartz glass cuvette. The 2 mm optical path was used for sample excitation, while the 10 mm path was used to probe the induced absorption changes. The protein concentration was adjusted to result in an optical density of ~1 OD per 10 mm.

### Kinetic analysis of time-resolved spectroscopic data obtained at neutral pH conditions

Time-resolved spectroscopic data were analyzed using both lifetime distribution analysis (LDA) and global lifetime analysis (GLA) with OPTIMUS software, available at www.optimusfit.org. When retrieved from LDA results, lifetime values were derived from the center of the respective lifetime distribution. Therefore, lifetimes are provided as approximate values, as the whole distribution describes the observed transition. The lifetimes obtained for the additionally performed GLA can be retrieved from the associated DAS.

### Flash photolysis at acidic pH

The pH dependence of calcium inhibition was investigated using time-resolved adsorption spectroscopy. A series of measurements was performed over the pH range 6–3 with a step size of 1.00 ± 0.05 in a sodium citrate buffer (0.1% DDM, 140 mM NaCl, 10 mM Na-citrate, adjusted with HCl). A set of pH values was obtained with and without the addition of 60 mM CaCl_2_. The buffer system was selected based on its wide working range. The citrate concentration was deliberately kept low to prevent the rapid precipitation of insoluble calcium salts. All measurements were performed soon after obtaining the buffer to prevent changes in the concentrations of its components. The sample was illuminated with a short flash at 510 nm. The protein for the experiments was concentrated to 150 mM NaCl and, before measurement, diluted into a prepared buffer by more than a factor of 50. Dilution factors were identical for paired samples (with and without calcium) at each pH value to ensure equivalent ionic strength and protein concentration across conditions. Excitation/detection systems were configured as follows: a Brilliant B laser with an OPO Rainbow (Quantel, France) was used, providing 4-ns pulses at 510 nm with an energy of ~1.4 mJ per pulse. Samples (5 × 5–mm spectroscopic quartz cuvette; Hellma GmbH & Co.) were placed in a thermostated house between two collimated and mechanically coupled monochromators (LOT MSH150, Germany). The probing light (xenon arc lamp, 75 W, Hamamatsu, Japan) passed through the first monochromator and, after a second monochromator, reached a photomultiplier tube (PMT) detector (R12829, Hamamatsu). The current-to-voltage converter of the PMT determines the time resolution of the measurement system of ca. 50 ns (measured as an apparent pulse width of the 5-ns laser pulse). Two digital oscilloscopes (Keysight DSO-X4022A) were used to record the traces of transient transmission changes in two overlapping time windows. The maximal digitizing rate was 10 ns per data point. Transient absorption changes were recorded from 10 ns after the laser pulses until the phototransformation was complete. At each wavelength, 15 laser pulses were averaged to improve the signal-to-noise ratio. The quasilogarithmic data compression reduced the initial number of data points per trace (~32,000) to ~850 points, evenly distributed on a log timescale, yielding ~100 points per time decade. The wavelengths were varied from 300 to 700 nm in 10 nm increments using a computer-controlled step motor. Absorption spectra of the samples were measured before and after each experiment on a standard spectrophotometer (Avantes Avaspec 2048 L).

### Statistics and reproducibility

Statistical parameters, including data dispersion and precision measures (mean and s.d.), are reported, where applicable. A number of experiments for each dataset is typically *n* = 1 for individual measurements, unless stated otherwise. Crystals for data collection were randomly selected from the crystallization well. MD simulations were repeated *n* = 3 times; individual trajectories are reported. No data were excluded from the analysis. No statistical method was used to predetermine the sample size. The experiments were not randomized. The investigators were not blinded to allocation during experiments and outcome assessment.

### Reporting summary

Further information on research design is available in the Nature Portfolio Reporting Summary linked to this article.

## Supplementary information

Supplementary Information file

Description of Additional Supplementary Files

Supplementary Data 1

Reporting Summary

Transparent Peer Review file

## Source data

Source data

## Data Availability

The atomic coordinates and structure factors generated in this study have been deposited in the Protein Data Bank under accession codes 9SOD (OLPVR1 Ca-surface state), 9SOE (OLPVR1 Ba-surface state), 9SOF (OLPVR1 Ca-inner state), and 9SOG (OLPVR1 Ca-blocked state). Previously published OLPVR1 structures used in this study are available under accession codes 9IN7 (ground state), 9IN8 (open state), and 9IN9 (low pH state). The source data, including raw time-resolved spectroscopy, ATR-FTIR, AFM, thermal stability and nanoDSF data, as well as all other reported data, were provided as Source Data files. Initial and final configurations for molecular dynamics simulations are available on the Zenodo repository [https://doi.org/10.5281/zenodo.20798399]. Source data are provided with this paper.
