# Supplementary material for: Molecular mechanism of calcium inhibition in viral channelrhodopsins
Source: Nat Commun. 2026 Sep 16;17:9875. doi: 10.1038/s41467-026-77716-5 (PMC13582892; doi:10.1038/s41467-026-77716-5)
Supplement: Supplementary file 1 [file 41467_2026_77716_MOESM1_ESM.pdf]

Supplementary Note

Biased localization of VCR1s in eukaryotic membranes

Cellular membranes generally exhibit considerable diversity in lipid content, permeability, viscosity, and other parameters[1]. The localization of integral membrane proteins depends to a larger degree on lipid composition, membrane thickness, and the presence of signal peptides. The targeted localization of MRs to different mammalian membranes has been thoroughly studied. While a combined approach using genetic modification has shown dramatic improvements for many chlorophyte channelrhodopsins, VCR1s have been shown to preferentially localize to internal membranes rather than the plasma membrane (PM)[2,3]. Our previous efforts to target VCR1s to the PM using various signal and membrane-trafficking peptides were unsuccessful. Moreover, VCR1s showed virtually no PM-associated signal across all constructs tested[3]. Thus, we hypothesize that the localization of VCR1s in internal membranes is essential for their function and is structurally predisposed. To understand the structural adaptation of VCR1s to different membrane compartments, we calculated the positioning of OLPVR1 in mammalian PM and ER membranes using the PPM server[4]. The resulting positioning showed that OLPVR1 adapted well to the ER membrane but not to the PM, where it was significantly tilted, preventing it from exposing the ECL2 loop to the surface (Figure 1). In contrast, the juxtamembrane ICL2 helix was observed in a similar orientation for both structures and was not submerged in the membrane. While the composition of the ICL2 helix and short TM4 helix likely affects membrane localization of OLPVR1, a broad generalization of similar concepts to all VCR1s requires a statistical justification.

Phylogenetic analysis of VCR1s reveals that the ECL2 helix is highly conserved among VCR1s, a characteristic not observed in other channelrhodopsin groups (Supplementary Figures 17 and 18). We used the TMHMM server to analyze the TM helix content of VCR1s and compared it to that of chlorophyte channelrhodopsins (CCRs) and class A GPCRs. Using the prediction results, we calculated the average length of each TM helix across all three groups and compared them using the Student's t-test. Surprisingly, not only do VCR1 rhodopsins have the shortest helices among the three groups, but also their TM4 helix consists of only 17.5 ± 1.8 (confidence interval for the mean of 95%, CI) residues, which is more than 3 residues shorter than the TM4 helix in CCRs and GPCRs (Supplementary Figure 18d). Given that the mean hydrophobic length of TM

domains in ER and PM membranes is 20.4 and 24.4 residues, respectively[5], the TM4 helix is likely to destabilize in the PM environment and, therefore, is a primary reason for VCR1 biased localization. On the other hand, the ER membranes have a lower bilayer thickness and are significantly more adaptable to the TM lengths of different membrane proteins[6].

To further investigate the features of the interaction of OLPVR1 with the surrounding membrane, 1000 ns-long molecular dynamics simulations of the monomeric (Supplementary Figure 14) and dimeric (Supplementary Figure 15) OLPVR1 with the membranes mimicking the natural mammalian ER and plasma membranes were conducted. In all simulations, we observed membrane deformation induced by OLPVR1. The membranes around the OLPVR1 are bent toward the C-terminus near TMs 1, 6, and 7 (~3-4 Å). Moreover, the ER membrane in this region is thickened (~ 3-4 Å), and in addition to this, the ER membrane and, especially, the plasma membrane are compressed near the TMs 3,4,5 (~ 2-3 Å and ~ 5-6 Å, respectively).

Calcium dependence of the OLPVR1 photocycle disappears at low pH.

As we show in the previous chapters, the binding of calcium inside the OLPVR1 channel depends on the protonation states of the key glutamates, E44 and E51. Our previously reported atomic-resolution structure of OLPVR1 at pH 2.5 (PDB ID: 9IN9) showed flips of both E44 and E51, suggesting their protonation at acidic pH or protonation of neighboring residues. This could alter Ca2+ interactions with the protein, thereby influencing calcium's ability to inhibit OLPVR1's channeling function. The pH dependence of calcium-induced inhibition was investigated using time-resolved spectroscopy (Supplementary Figure 5). A series of measurements was conducted over the pH range 6 to 3, where deprotonation/protonation of the key glutamates is expected. For each pH value, parallel measurements were performed in the presence of 60 mM $CaCl_2$. The kinetic data in the pH range 6 to 4 were broadly similar to the data presented for pH 8 (Figure 2). Representative kinetic traces of OD changes over time at pH 3, with and without $CaCl_2$, are presented in the corresponding figures. Notably, we did not observe any significant differences between the datasets in the absence of calcium and in the presence of 60 mM of $CaCl_2$.

Importance of Ca^{2+} for phototaxis

As discussed in our previous works[2,7], VCR1s comprise a phylogenetically distinct group of light-gated ion channels that are evolutionarily specific to nucleocytoplasmic large DNA viruses

(NCLDVs, giant viruses). Unlike many other MRs that have undergone lateral gene transfer (LGT) between marine species, VCR1s are almost exclusively found in NCLDV genomes, suggesting their importance for viral metabolism. Previously, the VCR1s were hypothesized to be involved in the bioenergetics of the infected cell, similar to light-gated proton pumps, such as proteorhodopsins[8]. This hypothesis is based on the experimental evidence that one of the VCR1s, DTS rhodopsin, showed a light-induced pH change when expressed in *E. coli* cells. However, light-induced ion channels can induce similar light responses when expressed in cellular membranes. Further experiments demonstrated that multiple VCR1s function as light-gated ion channels, as determined by electrophysiological methods in both cellular and artificial membranes.

Many channelrhodopsins regulate phototaxis and motility in the cells and often reside in specific organelles. The ChR1 and ChR2 channelrhodopsins from green algae *Chlamydomonas reinhardtii* are among the most well-studied ion channels that were found to enable the flagellar motion[9,10]. The ChR1 and ChR2 channelrhodopsins differ in function, mediating responses to low- and high-intensity light, respectively[11,12]. Under low-light conditions, changes in light-induced photocurrent control the flagellar beating and the direction of cell swimming[13]. Under high-light conditions, the prolonged activity of channelrhodopsins causes the subsequent activation of voltage-dependent Ca^{2+} channels in the flagellar membrane, triggering the photophobic response[14]. Both responses are regulated by calcium ions, which mediate secondary amplification related to the light trigger. Lowering calcium concentration in the cell suppresses the flagellar motion, and adding chelating agents abolishes the motion[9].

The existence of TM8 domains in VCR1 rhodopsins

We found six VCR1 rhodopsins with extended C-terminal domains, all of which encode a transmembrane helical domain (Supplementary Figure 21). Upon closer inspection, we found that the C-terminal extension comprises at least three separate structural domains: an ICL4 juxtamembrane helix domain, a proline-rich domain (PRD), and a transmembrane helix 8 (TM8). Unlike previously reported 8TM enzymerhodopsins with N-terminal TM0 helix[15], the C-terminal TM helices have not yet been reported in microbial rhodopsins. The motif search in the C-terminal domain sequences has identified similarity to prokaryotic cytochrome b561[16]. Interestingly, proline-rich domains with the PxxP motif are often found in SH3-domain proteins,

which are involved in signaling pathways regulating the cytoskeleton and tyrosine kinase activity[17]. While microbial rhodopsins, and especially eukaryotic rhodopsins, sometimes have elongated C-terminal domains (exceeding 100 aa), the functional importance of these domains remains unclear[18]. Hypothetically, the TM8 helix can serve as a binding domain that couples VCR1 rhodopsins with some other integral membrane proteins, such as BI-1 proteins, that can be directly activated by the Ca^{2+} waves[19]. These and other possibilities further highlight the functional importance of VCR1 rhodopsins and their flexible role for giant viruses.

Side-chain fluctuations during the molecular modeling experiments

The molecular dynamics simulations reveal distinct dynamical behavior among residues located near the intracellular gate of OLPVR1 (Supplementary Figure 11). We analyzed the fluctuations of 4 key residues in the region: E44, Q48, T87, and T88. We split the residues into two groups based on side chain length and added a reference residue outside the IG region, resulting in group 1 comprising E44, Q48, and E66, and group 2 comprising T87, T88, and T60 (Supplementary Figure 12a). Within Group 1, the residues E44 and Q48 display side-chain fluctuations comparable or slightly smaller than the reference residue E66, indicating enhanced conformational flexibility in the intracellular region surrounding the gate. In contrast, residues T87 and T88 in Group 2 remain stable across all simulations, with RMSF values at least 2 times lower than T60 (Supplementary Figure 12d). Comparisons between individual experiments show that, in general, the residues are not affected by ion replacement, with 2 notable exceptions. First, Q48 fluctuations are significantly higher in the ground state than in the Ca-blocked state, regardless of the ion, with average RMSDs of 1.55 Å and 0.38 Å, respectively. That indicates that Q48 is likely flexible in the ground state, but is locked in the Ca-blocked state. Second, the fluctuations of E44 are ion-dependent, with the lowest mobility in the ground state (av_RMSD = 0.7 Å), followed by different RMSDs depending on the ion, with av_RMSDs of 1.17, 1.23, and 1.53 Å, in the case of sodium, water, and calcium modeled in site B, respectively (Supplementary Figure 12b). These results correlate with the predicted ion displacement (Supplementary Figure 11) and suggest that molecular modeling indicates that water and sodium ions can be accommodated at site B during the 500 ns simulations.

Supplementary References

1. Harayama, T. & Riezman, H. Understanding the diversity of membrane lipid composition. *Nat. Rev. Mol. Cell Biol.* **19**, 281–296 (2018).
2. Zabelskii, D. *et al.* Viral rhodopsins 1 are an unique family of light-gated cation channels. *Nat. Commun.* **11**, 5707 (2020).
3. Eria-Oliveira, A.-S. *et al.* Hijacking of internal calcium dynamics by intracellularly residing viral rhodopsins. *Nat. Commun.* **15**, 65 (2024).
4. Lomize, A. L., Todd, S. C. & Pogozheva, I. D. Spatial arrangement of proteins in planar and curved membranes by PPM 3.0. *Protein Sci.* **31**, 209–220 (2022).
5. Sharpe, H. J., Stevens, T. J. & Munro, S. A Comprehensive Comparison of Transmembrane Domains Reveals Organelle-Specific Properties. *Cell* **142**, 158–169 (2010).
6. Prasad, R., Sliwa-Gonzalez, A. & Barral, Y. Mapping bilayer thickness in the ER membrane. *Sci. Adv.* **6**, eaba5130 (2020).
7. Zabelskii, D. *et al.* Ion-conducting and gating molecular mechanisms of channelrhodopsin revealed by true-atomic-resolution structures of open and closed states. *Nat. Struct. Mol. Biol.* https://doi.org/10.1038/s41594-025-01488-7 (2025) doi:10.1038/s41594-025-01488-7.
8. Needham, D. M. *et al.* A distinct lineage of giant viruses brings a rhodopsin photosystem to unicellular marine predators. *Proc. Natl. Acad. Sci.* **116**, 20574–20583 (2019).
9. Harz, H. & Hegemann, P. Rhodopsin-regulated calcium currents in Chlamydomonas. *Nature* **351**, 489–491 (1991).
10. Hegemann, P. Algal Sensory Photoreceptors. *Annu. Rev. Plant Biol.* **59**, 167–189 (2008).

11. Sineshchekov, O. A., Jung, K.-H. & Spudich, J. L. Two rhodopsins mediate phototaxis to low- and high-intensity light in Chlamydomonas reinhardtii. *Proc. Natl. Acad. Sci.* **99**, 8689–8694 (2002).

12. Sineshchekov, O. A., Govorunova, E. G. & Spudich, J. L. Photosensory Functions of Channelrhodopsins in Native Algal Cells. *Photochem. Photobiol.* **85**, 556–563 (2009).

13. Schaller, K., David, R. & Uhl, R. How Chlamydomonas keeps track of the light once it has reached the right phototactic orientation. *Biophys. J.* **73**, 1562–1572 (1997).

14. Beck, C. & Uhl, R. On the localization of voltage-sensitive calcium channels in the flagella of Chlamydomonas reinhardtii. *J. Cell Biol.* **125**, 1119–1125 (1994).

15. Ikuta, T. *et al.* Structural insights into the mechanism of rhodopsin phosphodiesterase. *Nat. Commun.* **11**, 5605 (2020).

16. Asard, H., Barbaro, R., Trost, P. & Bérczi, A. Cytochromes *b* 561: Ascorbate-Mediated Trans-Membrane Electron Transport. *Antioxid. Redox Signal.* **19**, 1026–1035 (2013).

17. Kay, B. K., Williamson, M. P. & Sudol, M. The importance of being proline: the interaction of proline‐rich motifs in signaling proteins with their cognate domains. *FASEB J.* **14**, 231–241 (2000).

18. Sharma, K. *et al.* Deciphering the role of cytoplasmic domain of Channelrhodopsin in modulation of the interactome and SUMOylome of Chlamydomonas reinhardtii. *Int. J. Biol. Macromol.* **243**, 125135 (2023).

19. Ishikawa, T., Watanabe, N., Nagano, M., Kawai-Yamada, M. & Lam, E. Bax inhibitor-1: a highly conserved endoplasmic reticulum-resident cell death suppressor. *Cell Death Differ.* **18**, 1271–1278 (2011).

20. Sievers, F. & Higgins, D. G. Clustal Omega. *Curr. Protoc. Bioinforma.* **48**, (2014).

21. Robert, X. & Gouet, P. Deciphering key features in protein structures with the new ENDscript server. *Nucleic Acids Res.* **42**, W320–W324 (2014).

22. Hallgren, J. *et al. DeepTMHMM Predicts Alpha and Beta Transmembrane Proteins Using Deep Neural Networks*. http://biorxiv.org/lookup/doi/10.1101/2022.04.08.487609 (2022) doi:10.1101/2022.04.08.487609.

23. Fadini, A., Apostolopoulou, V., Lane, T. J. & Van Thor, J. J. Denoising Reveals Low-Occupancy Populations in Protein Crystals. Preprint at https://doi.org/10.1101/2024.11.06.622276 (2024).

24. Carrasco-Busturia, D., Linares, M., Norman, P. & Haugaard Olsen, J. M. Multiphoton Absorption Spectra of Channelrhodopsin-2 via Multiscale Simulation Methods. *J. Chem. Theory Comput.* **22**, 1133–1148 (2026).

25. Finn, R. D. *et al.* Pfam: the protein families database. *Nucleic Acids Res.* **42**, D222–D230 (2014).

Supplementary Figures

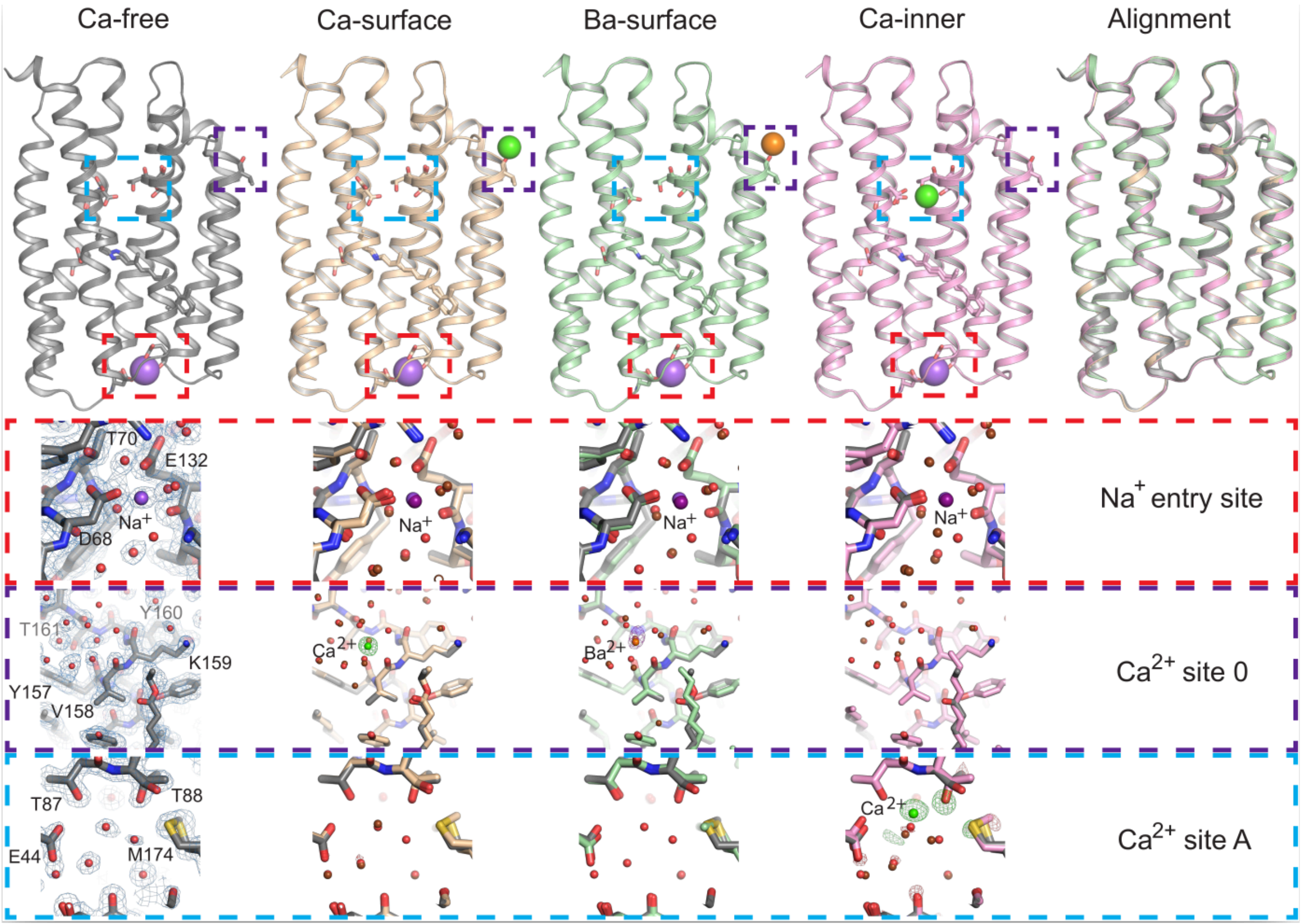

Supplementary Figure 1. Structures of OLPVR1 in complexes with calcium and barium ions. Structures of the Ca-free (PDB ID: 9IN7[9]), Ca-surface (PDB ID: 9SOD), Ba-surface (PDB ID: 9SOE), and Ca-inner (PDB ID: 9SOF) states are compared in the sodium entry site, Ca-binding site 0 at the surface, and Ca-binding site A in the channel interior. For the Ca-free state structure, the 2*m*Fo-*D*Fc map is contoured at the 1σ level. For the Ca-surface state structure, the $F_o^{Ca\text{-}surface}$-$F_o^{Ca\text{-}free}$ isomorphous difference map is contoured at the 5σ level. For the Ba-surface state structure, the anomalous map is contoured at the 5σ level. For the Ca-inner state structure, the $F_o^{Ca\text{-}inner}$-$F_o^{Ca\text{-}free}$ isomorphous difference map is contoured at a 5σ-level.

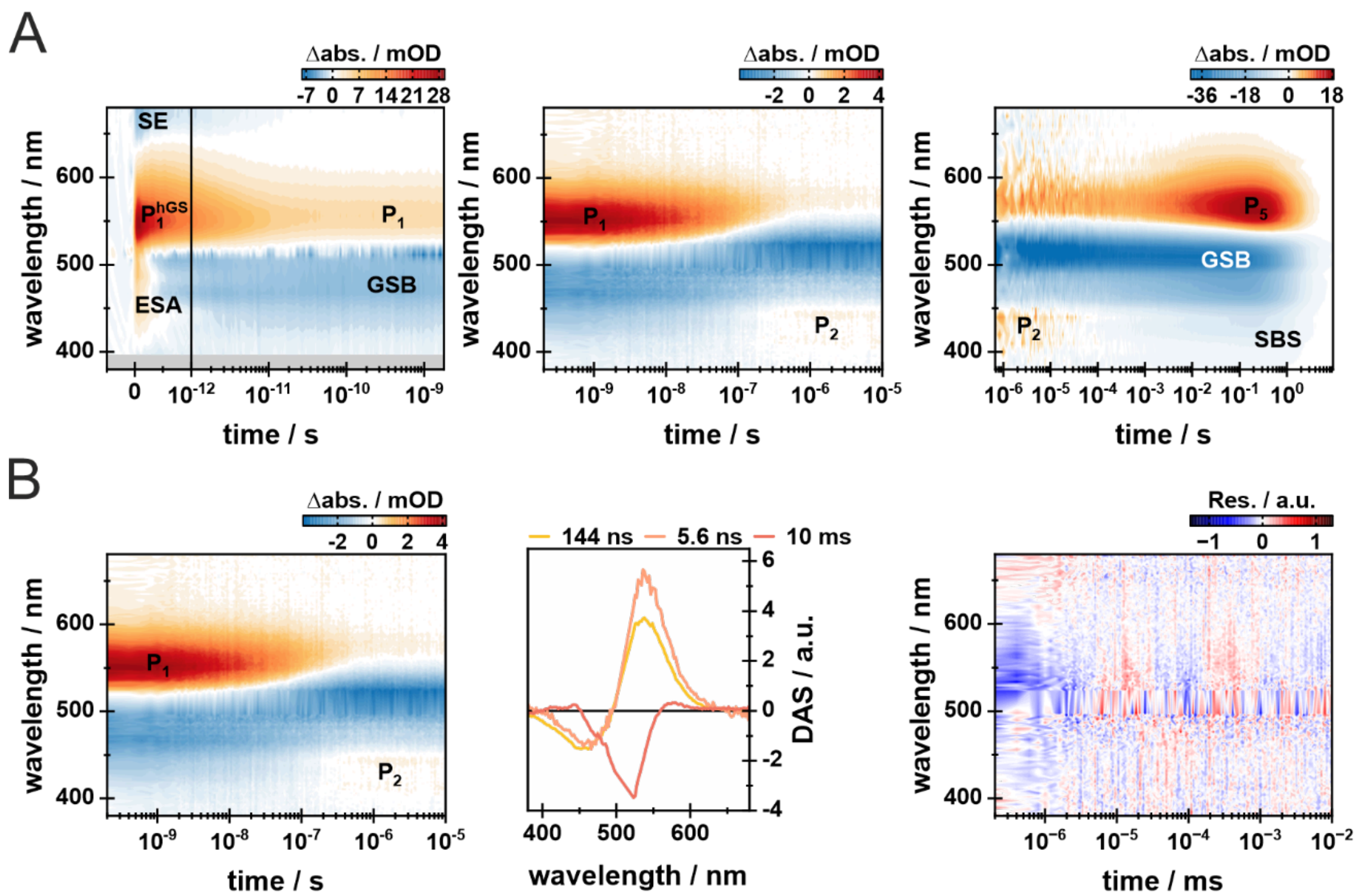

Supplementary Figure 2. Overview of the complete OLPVR1 wt photocycle from fs to s at pH 8.0 in the absence of sodium and calcium. A. Time-resolved UV/vis spectroscopic experiments of OLPVR1 wt at pH 8.0, 0 mM NaCl, 0 mM $CaCl_2$. The panels show the ultrafast dataset up to 1.8 ns (left), the dataset spanning from 200 ps up to 10 µs (middle) and the flash photolysis dataset covering the time interval from 670 ns to the end of the photocycle (right). **B.** Detailed view on the dataset covering the ns timescale, which was acquired with two synchronized fs laser systems. Data set (left) and DAS (middle) to retrieve the lifetimes and the residuals (right) of the fit. Wavelengths in the range 500–520 nm were excluded from plotting and fitting due to extensive scattering of the pump pulse. Thus, data in this range are extrapolated in the 2D contour plots and in the analysis representations.

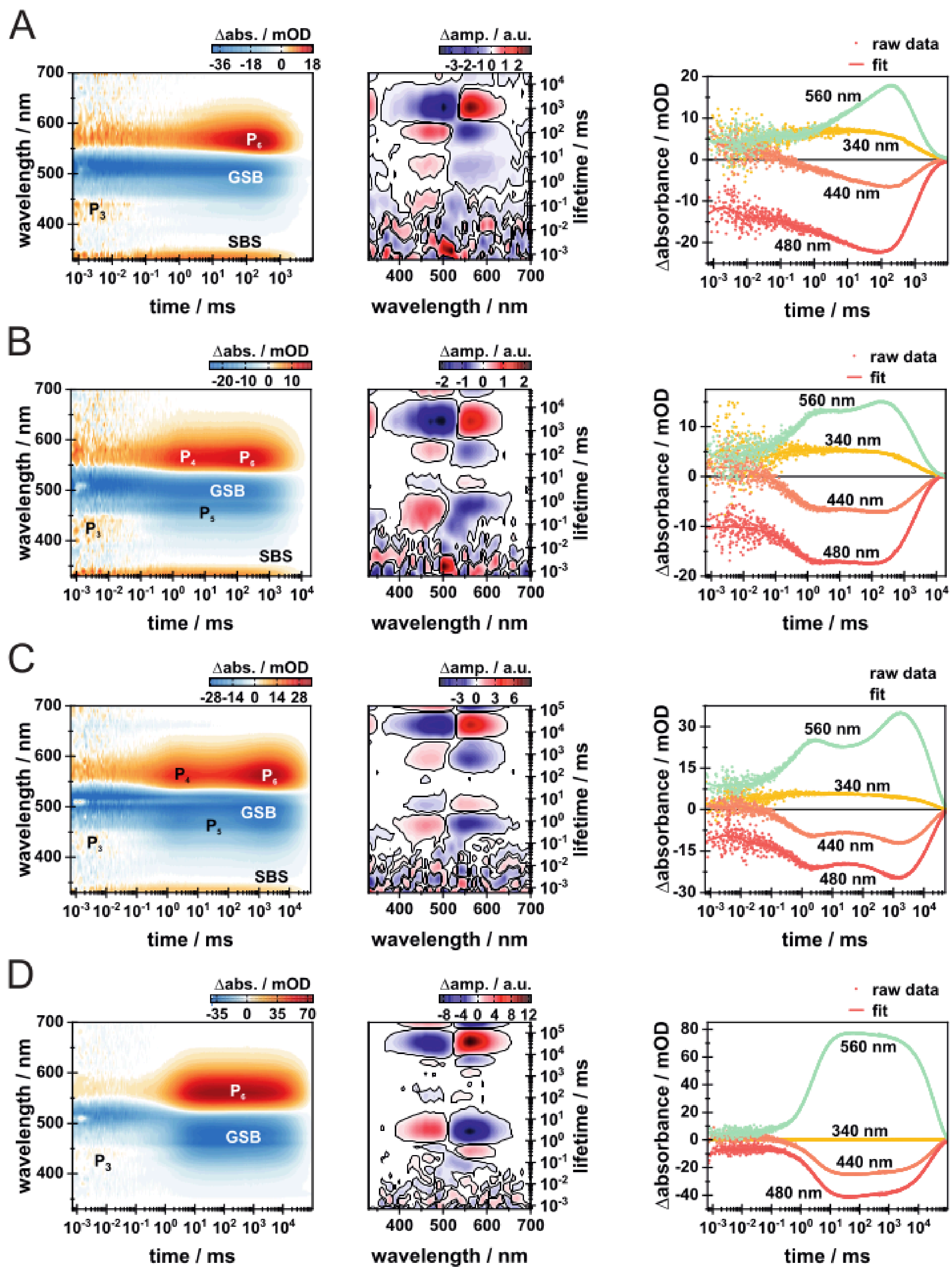

Supplementary Figure 3. Ion-dependent flash photolysis data of OLPVR1. Flash photolysis

measurements of OLPVR1 at pH 8.0, A. 0 mM NaCl, 0 mM CaCl2, B. 150 mM NaCl, 0 mM $CaCl_2$, C. 1000 mM NaCl, 0 mM $CaCl_2$, and D. 150 mM NaCl, 60 mM CaCl2 are shown as 2D-contour plots. Additionally, the corresponding lifetime distribution maps (LDMs) are shown, along with transients at characteristic wavelengths (340 nm, 440 nm, 480 nm, and 560 nm). The raw data is visualized as dots, while the fit is visualized as lines.

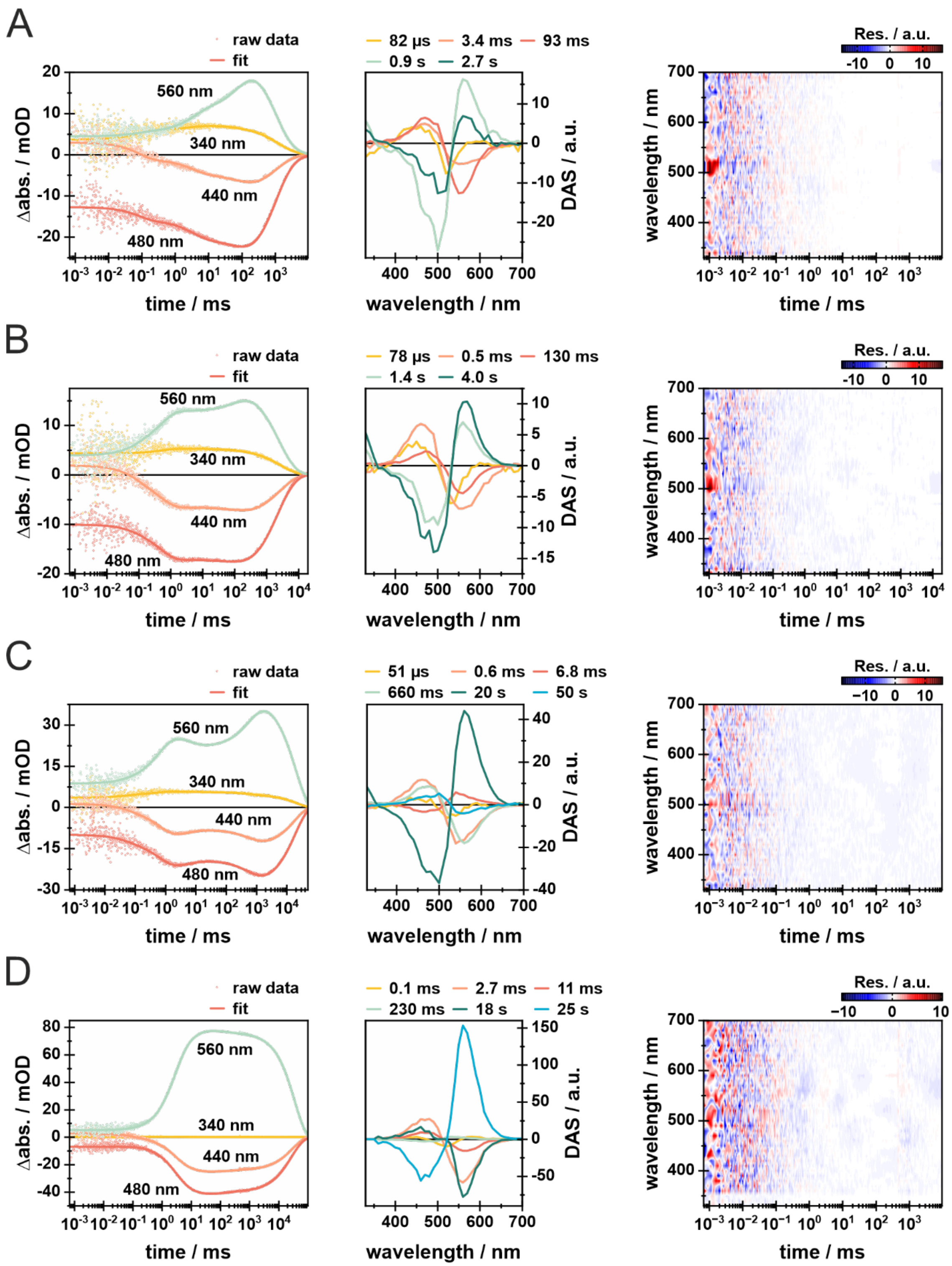

Supplementary Figure 4. Analysis of ion-dependent flash photolysis data using the global lifetime analysis method. Characteristic transients (left) of flash photolysis measurements of OLPVR1 at pH 8.0 **A.** 0 mM NaCl, 0 mM $CaCl_2$, **B.** 150 mM NaCl, 0 mM $CaCl_2$, **C.** 1000 mM

NaCl, 0 mM $CaCl_2$, and **D.** 150 mM NaCl, 60 mM $CaCl_2$. Additionally, the corresponding decay-associated spectra (DAS, middle) and the residuals (right) of the fitting procedure are shown.

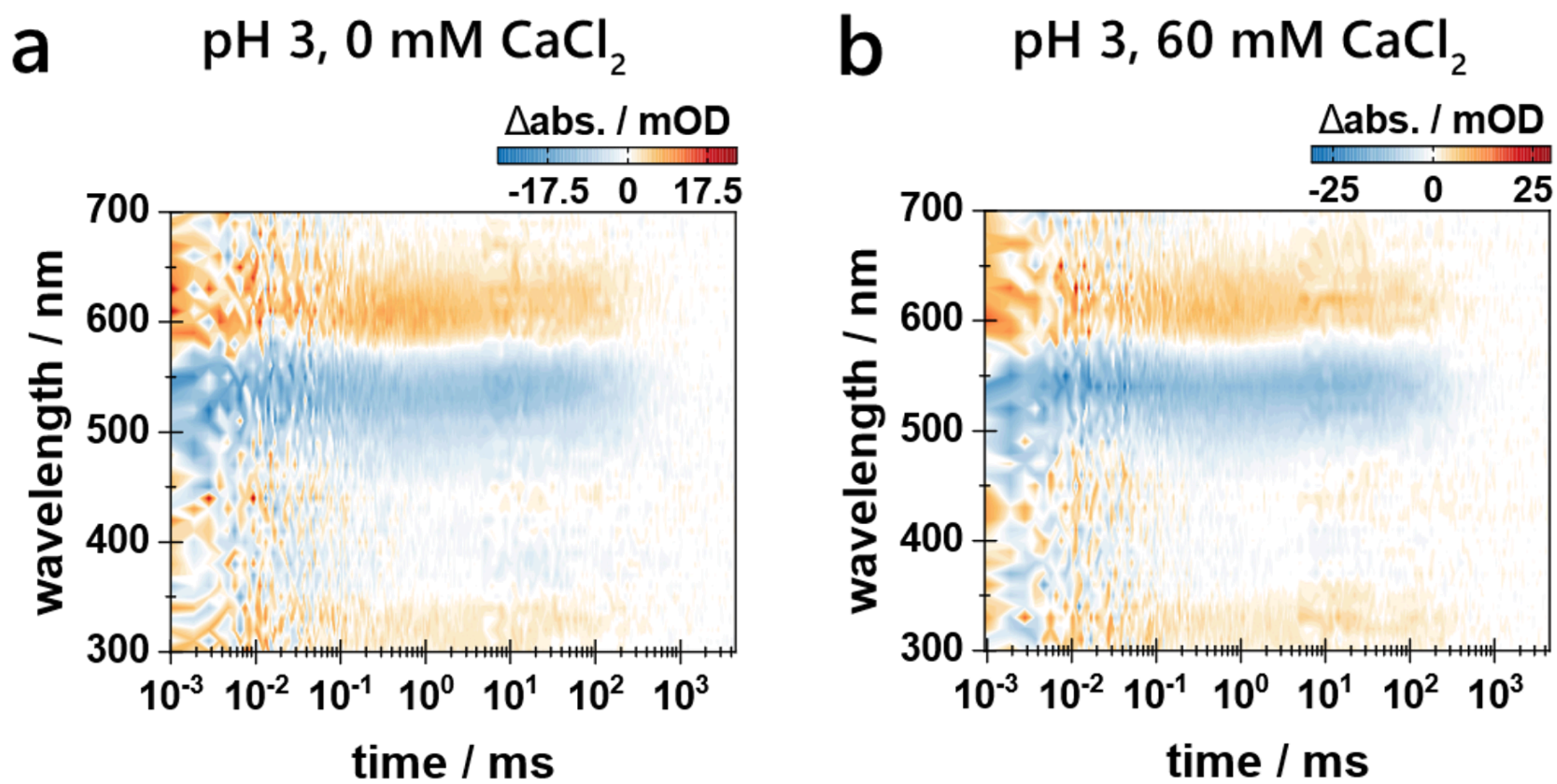

Supplementary Figure 5. Influence of calcium on OLPVR1 photocycles at pH 3. a. Transient absorption map of OLPVR1 photocycle at pH 3 in the absence of calcium. **b.** Transient absorption map of OLPVR1 photocycle at pH 3 in the presence of 60 mM $CaCl_2$.

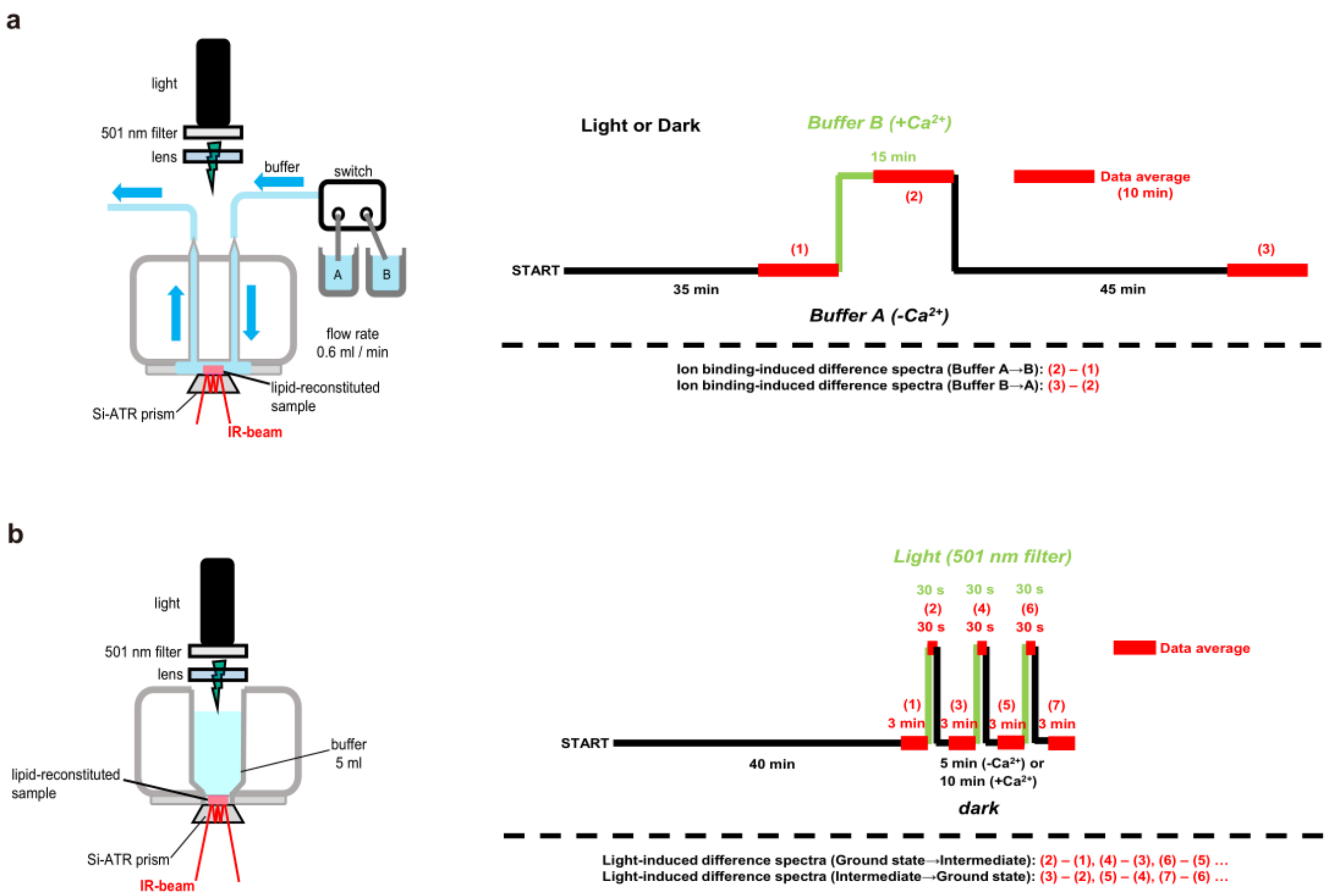

Supplementary Figure 6. Experimental setup of ATR-FTIR spectroscopy. a. Difference FTIR spectra obtained by buffer exchange using a flow system. Two buffers, A and B, were introduced alternately by a flow system, from which the presence-minus-absence difference FTIR spectra of Ca^{2+} were obtained under dark or light conditions. Buffer A does not contain Ca^{2+}, while buffer B contains various Ca^{2+} concentrations. b. Light-minus-dark difference FTIR spectra obtained by illuminating the sample in a buffer. The “light” spectrum was measured during illumination, while the “dark” spectrum was measured for the resting state of OLPVR1. The light-minus-dark difference FTIR spectra were averaged by repeating the measurement. As the photocycle of OLPVR1 was longer in the presence of Ca^{2+}, the measurement cycle was longer.

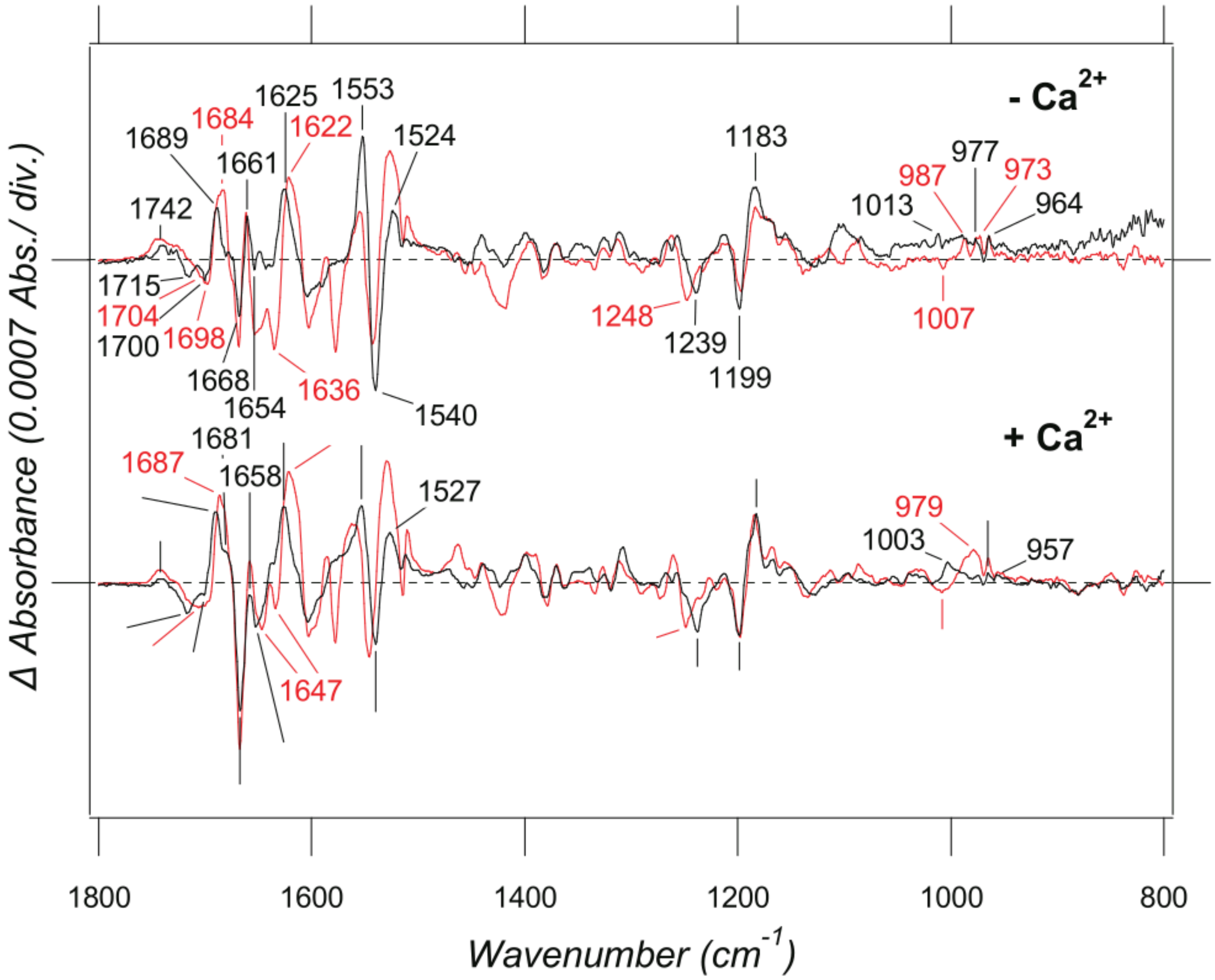

Supplementary Figure 7. Light-induced difference FTIR spectra of OLPVR1. Light-minus-dark difference FTIR spectra of OLPVR1 in the absence (Top) and presence (Bottom) of 5 mM $CaCl_2$. Spectra were measured in H_2O (black) and D_2O (red) at pH 8 and 283 K. In the presence of $CaCl_2$ (Bottom), the frequencies that differ from those in the absence are only labeled.

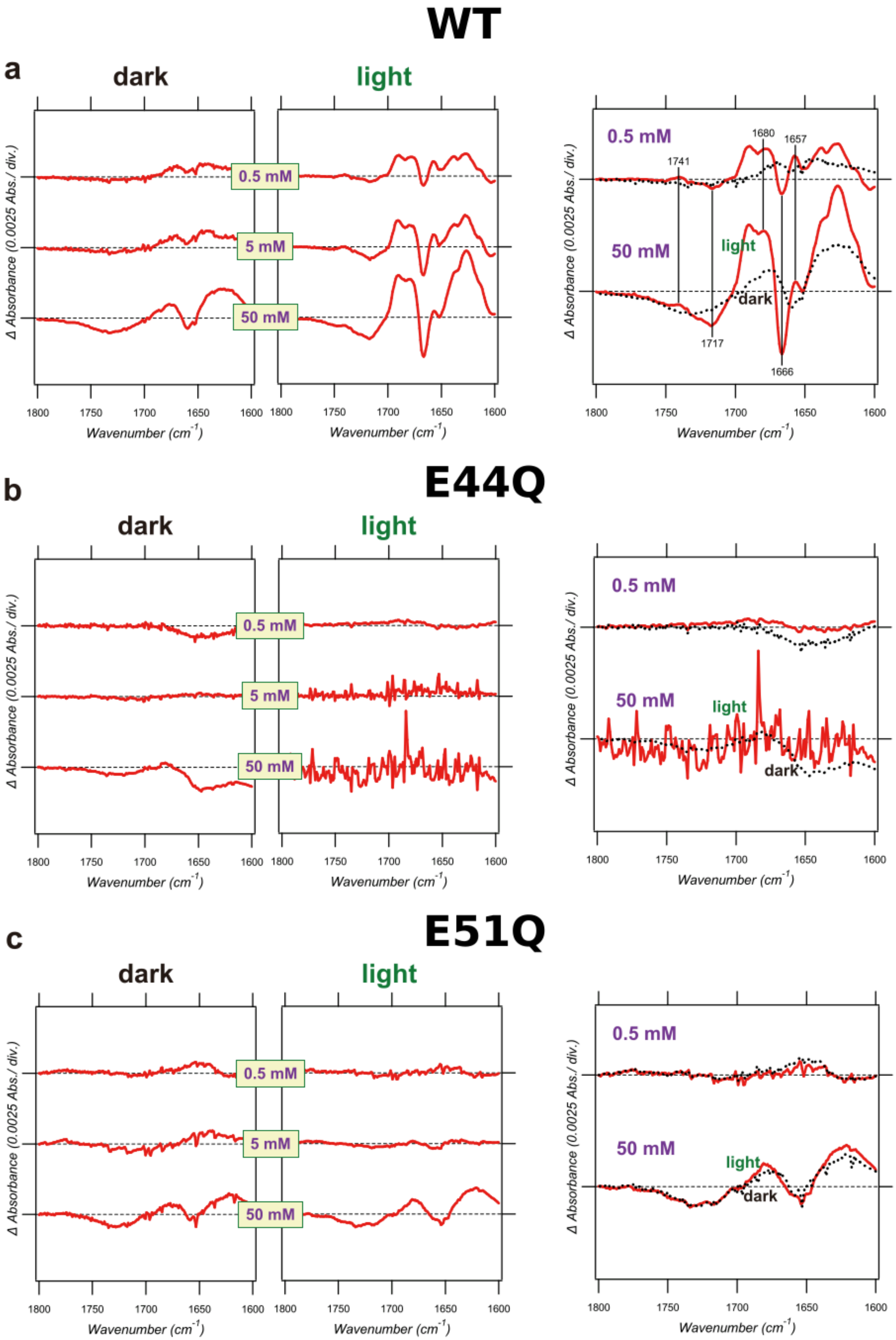

WT
a
dark
light
0.5 mM
5 mM
50 mM
Δ Absorbance (0.0025 Abs./ div.)
Wavenumber (cm-1)
1800
1750
1700
1650
1600
1741
1680
1657
1717
1666
E44Q
b
E51Q
c

Supplementary Figure 8. Calcium binding to the mutant proteins of OLPVR1 monitored by FTIR spectroscopy. The presence-minus-absence difference FTIR spectra of various Ca^{2+} concentrations for the wild-type (a), E44Q (b), and E51Q (c) under dark (Left) and light (Middle) conditions. Difference spectra were obtained using a buffer-exchange system at pH 8 and 283 K, and the sample was illuminated with 501 nm light. The presence-minus-absence difference FTIR spectra of 0.5 mM (Top) and 50 mM (Bottom) Ca^{2+} under dark (black dotted line) and light (red line) (Right).

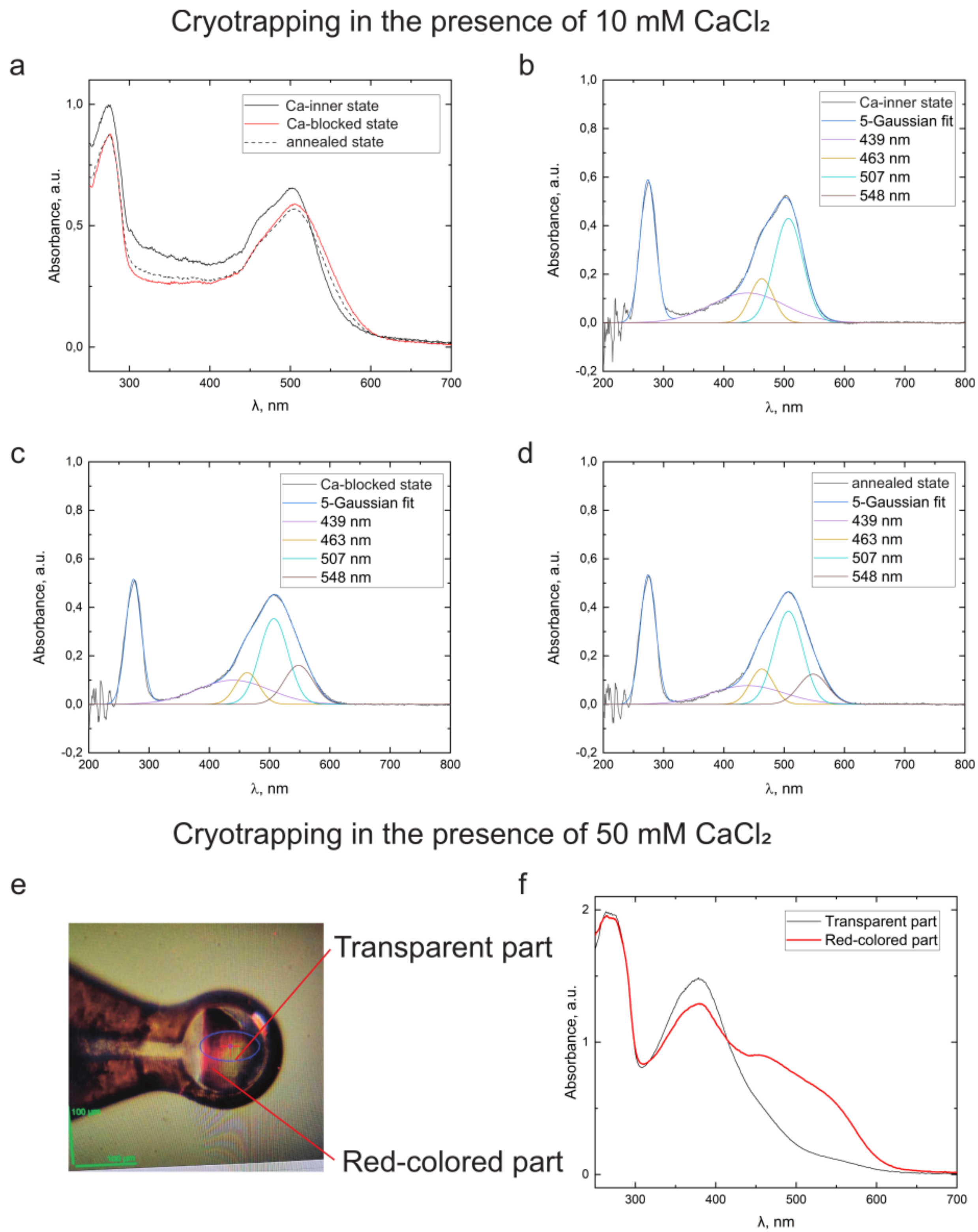

Supplementary Figure 9. Cryotrapping of OLPVR1 crystals in the presence of calcium. a. Cryotrapping of OLPVR1 crystals at RT with a 532 nm laser in the presence of 10 mM $CaCl_2$.

Spectra before trapping (Ca-inner state), after trapping (Ca-blocked state), and after annealing at RT for 2 seconds (annealed state) are shown. **b** to **d**. 5-Gaussian fit of Ca-inner state, Ca-blocked state, and annealed state, respectively. **e** A photo of the OLPVR1 crystal after its cryotrapping at RT with a 532 nm laser in the presence of 50 mM $CaCl_2$. **f**. Spectra corresponding to panel **e**.

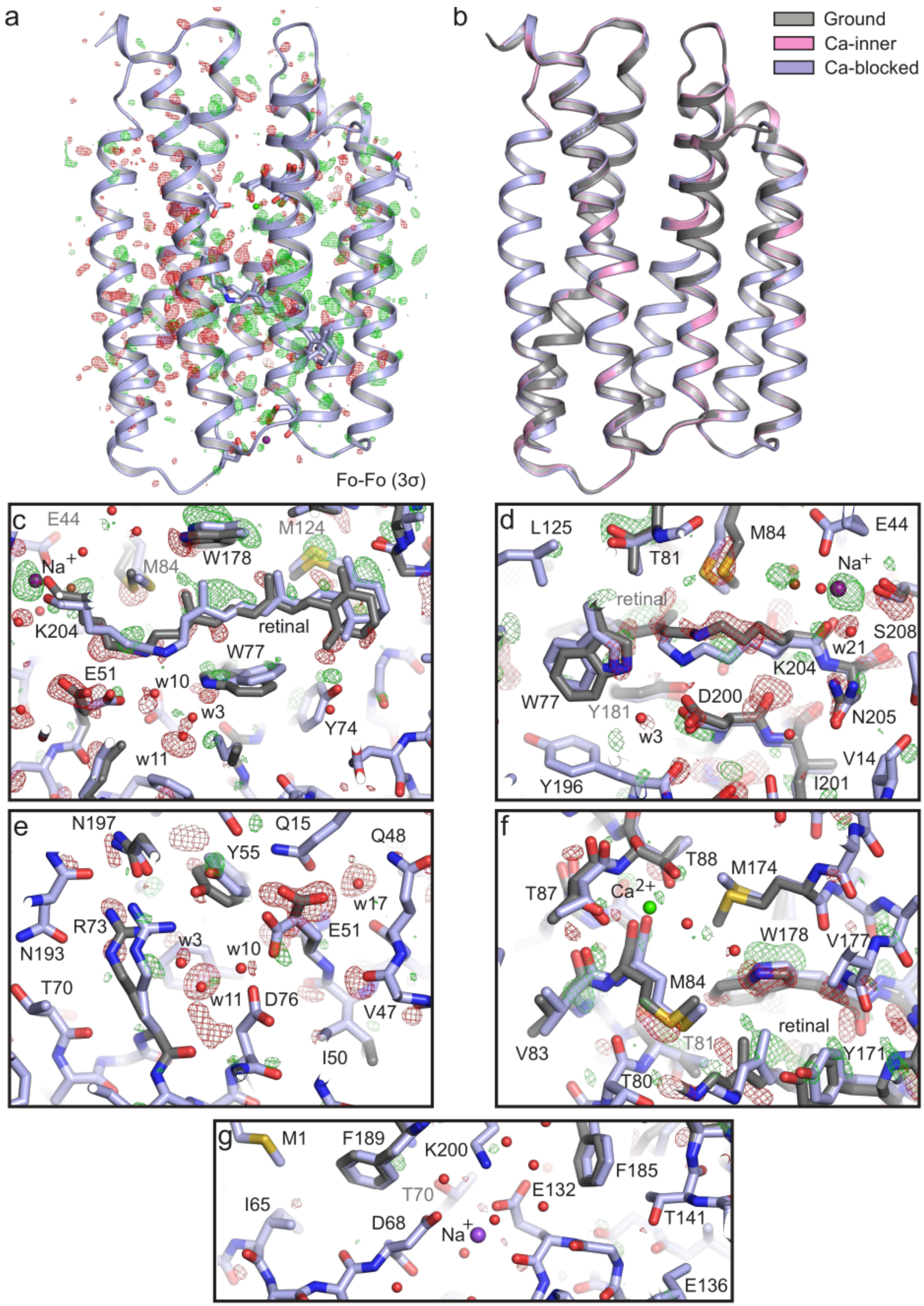

a
Fo-Fo (3σ)
b
Ground
Ca-inner
Ca-blocked
c
E44
Na+
M84
W178
M124
K204
retinal
W77
E51
w10
w3
Y74
w11
d
L125
T81
M84
E44
Na+
retinal
S208
w21
K204
W77
Y181
D200
N205
w3
V14
Y196
I201
e
N197
Q15
Q48
Y55
w17
R73
E51
N193
w3
w10
T70
w11
D76
V47
I50
f
T88
M174
T87
Ca2+
W178
V177
M84
V83
T81
retinal
Y171
T80
g
M1
F189
K200
F185
T70
E132
I65
T141
D68
Na+
E136

Supplementary Figure 10. Structure of the Ca-blocked state of OLPVR1. a. Overall structure of OLPVR1 in the Ca-blocked state. **b**. Alignment of OLPVR1 in the ground (gray, PDB ID: 9IN7[9]), Ca-inner (pink, PDB ID: 9SOF), and Ca-blocked (blue, PDB ID: 9SOG) states. **c**. RSB pocket. **d**. Central and Cytoplasmic gates. **e**. Extracellular gate. **f**. Ca-binding site A. **g**. Na-entry site. **c** to **g**. Non-photoactivated Ca-inner state (conf A, colored gray, occ=70%) and Ca-blocked state (conf B, colored blue, occ=30%) are depicted with sticks. $Fo^{Ca\text{-}blocked}$-$Fo^{Ca\text{-}inner}$ isomorphous difference map is contoured at a 3σ-level.

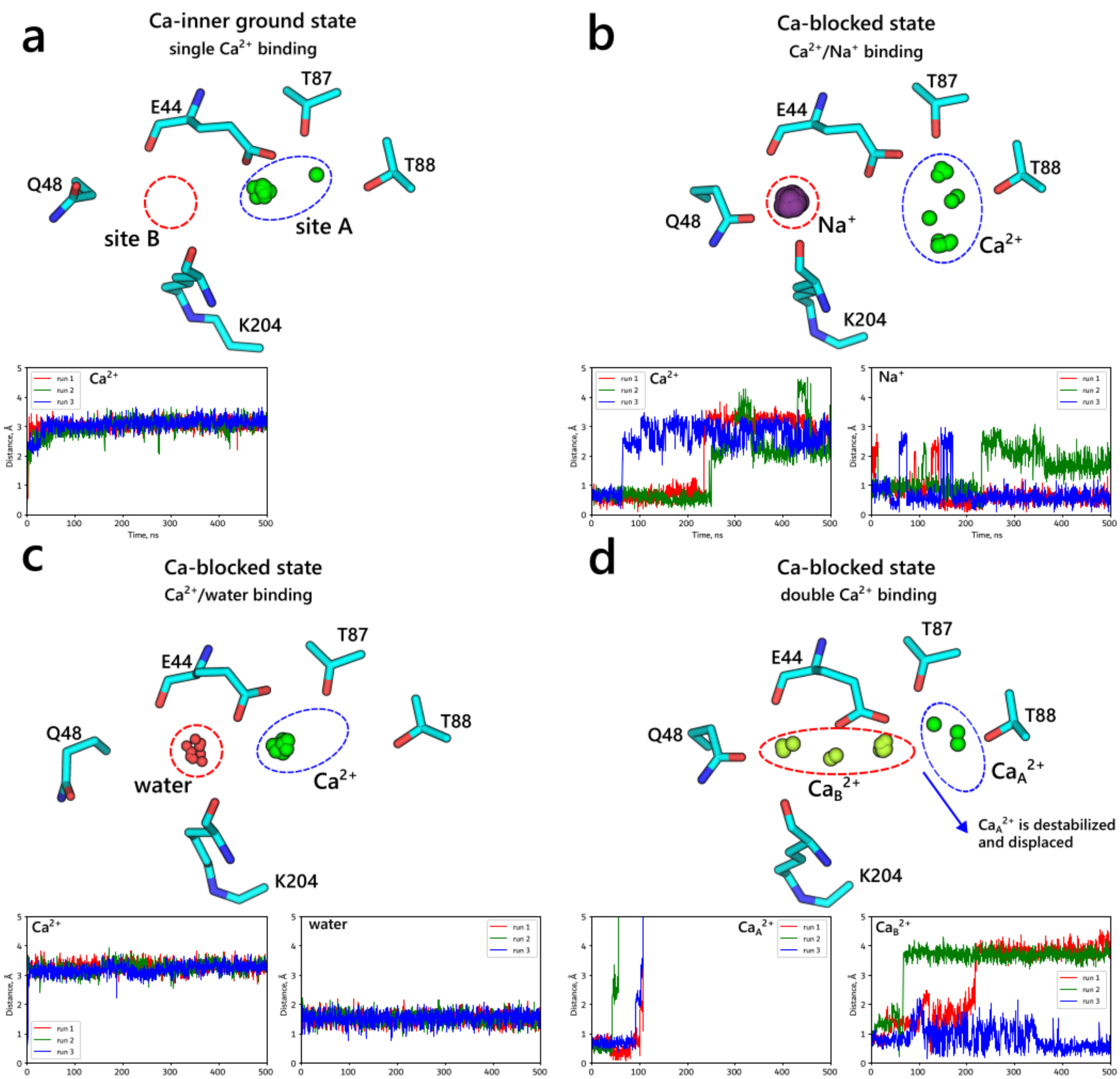

Supplementary Figure 11. Predicted stability of the ions in the OLPVR1 binding sites. a.

Magnified view of the internal ion-binding sites of OLPVR1 (top), and the displacement of ions from their initial positions as a function of time (bottom), calculated for the Ca-inner ground state structure. **b-d** For the Ca-blocked state, the electron density at site B was modeled as sodium, water, and calcium ions, with the corresponding results presented in subplots b, c, and d, respectively. The positions of sites A and B are shown as dashed blue and red ellipses, respectively. Three independent replicas (n=3) were performed for each experiment, and the ion positions are shown for the first replica. In the case of the double calcium-binding experiment, the calcium in site A was displaced from the intracellular gate within 100 ns and, therefore, is not present in the area of interest. In all simulations, E44 was deprotonated, and E51 was protonated.

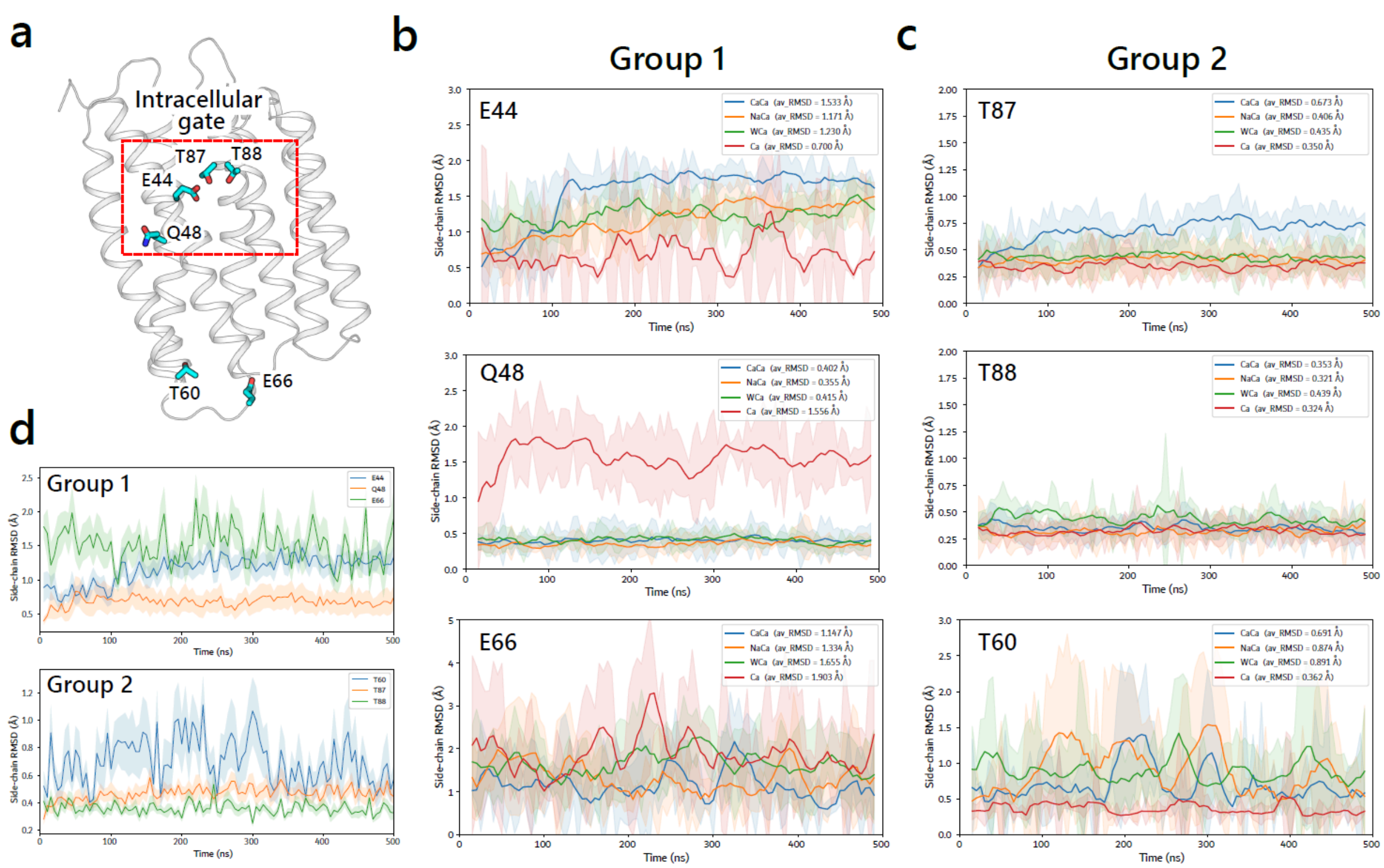

Supplementary Figure 12. Side-chain dynamics of intracellular-gate residues in OLPVR1 revealed by molecular dynamics simulations. a. Structural overview of OLPVR1, highlighting the residues analyzed in this study. The protein is shown as a grey cartoon, with selected side chains shown as cyan sticks. The region proposed to form the intracellular gate (E44, T87, and T88) is indicated by a dashed red box. **b.** Time-dependent side-chain RMSD for Group 1 residues (E44, Q48, and E66). RMSD values were calculated after aligning the backbone atoms of the corresponding residue to remove local backbone motion. Curves represent the smoothed

mean across three independent trajectories, with shaded regions indicating the standard deviation. Four simulation conditions are shown: CaCa (blue), NaCa (orange), WCa (green) and Ca (red). Average RMSD values over the trajectory are indicated in the legend. **c.** Same analysis for Group 2 residues (T87, T88, and T60). Compared with Group 1, these residues display generally smaller fluctuations and weaker condition-dependent differences. **d.** Group-averaged RMSD profiles obtained by averaging the residue-wise traces within Group 1 (E44, Q48, and E66) and Group 2 (T60, T87, and T88). Group 1 exhibits greater conformational fluctuations and stronger dependence on conditions, whereas Group 2 remains comparatively stable throughout the simulations. All RMSD values are reported in Å, the time axis is shown in nanoseconds, and the initial relaxed frame was omitted from the analysis.

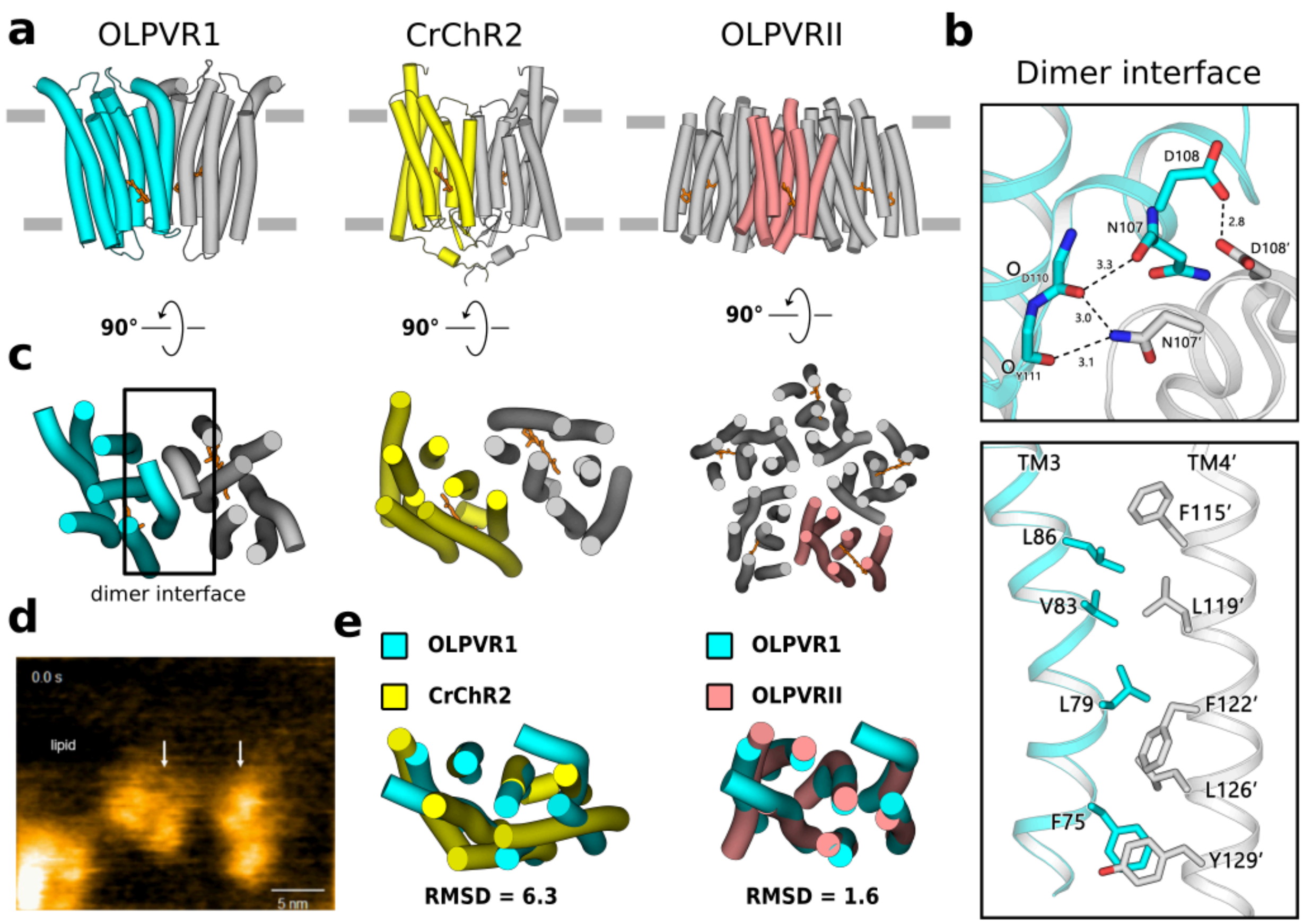

Supplementary Figure 13. Dimer interface of OLPVR1. a. Comparison of OLPVR1 dimer (PDB ID: 9IN7[9]), CrChR2 dimer (PDB ID: 6EID[15]), and OLPVRII pentamer (PDB ID: 6SQG[45]) viewed from the membrane plane. The retinal cofactor is shown with orange sticks. The

membrane boundaries are shown with gray rectangles. **b.** Magnified view of the OLPVR1 dimer interface on the intracellular side (top) and the hydrophobic interface in the membrane part. c. Comparison of OLPVR1 dimer, CrChR2 dimer, and OLPVRII pentamer viewed from the intracellular side. d. A representative HS-AFM image of the OLPVR1 dimer in the lipid membrane. Frame rate, 2.0 frames per second. e. Alignment of individual monomers of OLPVR1 with ChR2 and OLPVR1 with OLPVRII. Root-mean-square deviations (RMSD) were calculated using PyMOL.

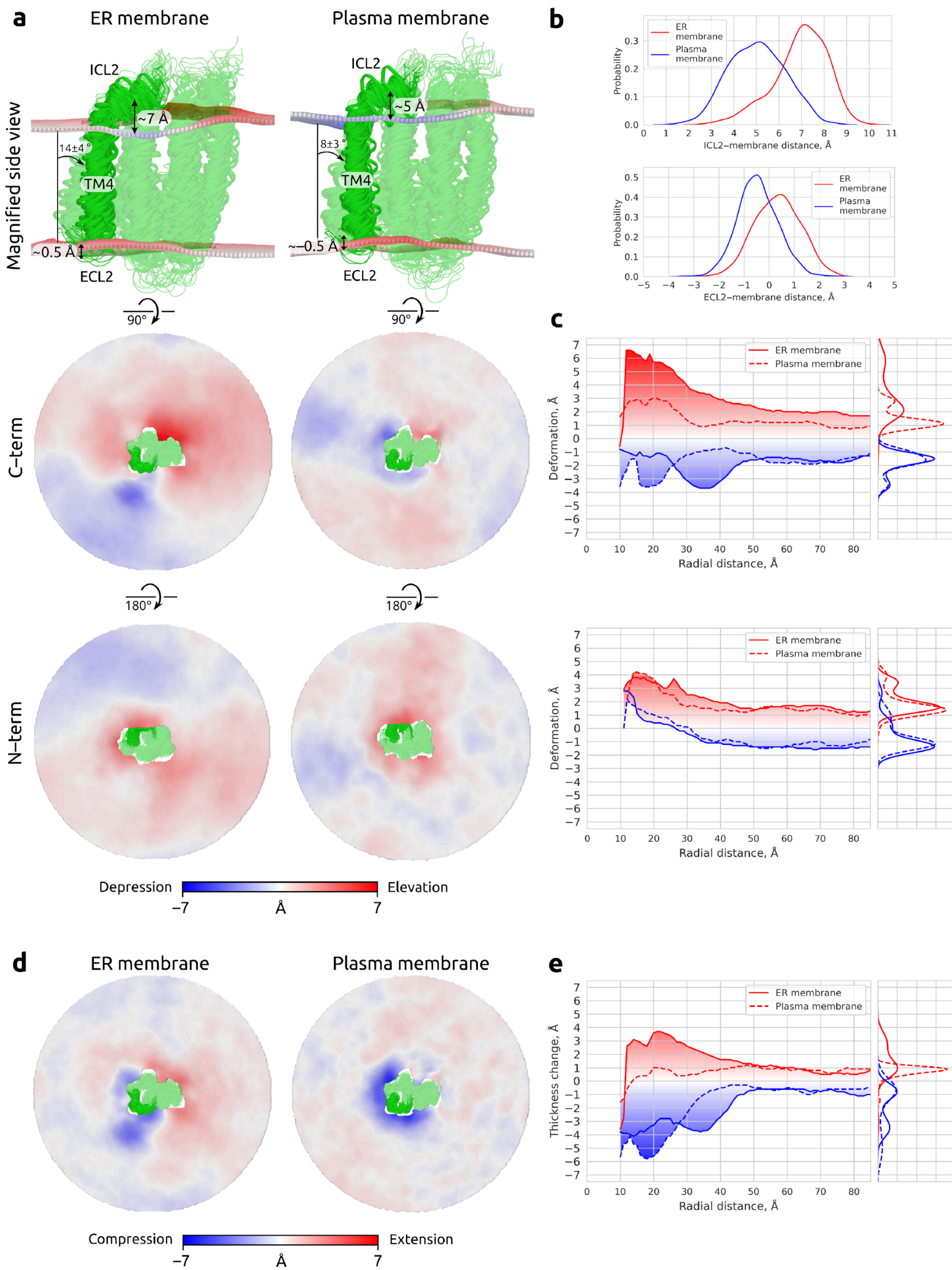

Supplementary Figure 14. Properties of the monomeric OLPVR1. a. Membrane deformation around the monomeric OLPVR1. Membrane borders are depicted as surfaces colored red when

elevated and blue when depressed. **b**. Distributions of Z-distances between the center of mass (COM) of ECL2 or ICL2 Cα atoms and the COM of lipid Cα carbon atoms within 10 Å of protein Cα atoms. **c**. Extreme deformation values in annuli of width 1 Å around the monomer. The maximum elevation values are colored red, the minimum depression values are colored blue, and the range of observed deformation values is colored with a blue-white-red gradient. Values do not start at zero because there are no lipids in that region. The ER-like membrane is more bent around the OLPVR1. **d**. Thickness changes in the membranes around the monomeric OLPVR1. Membrane compression and extension are shown in blue and red, respectively. e. Extreme values of thickness change in annuli of width 1 Å around the monomer. The maximum extension values are colored in red, and the minimum compression values are colored in blue. The range of observed thickness change values is colored using a blue-white-red color gradient. The ER and plasma membrane are thinned near the TMs 3, 4, and 5. The ER membrane is also thickened near TM1,6,7. The OLPVR1 is represented by 20 conformations taken every 50 ns. The TM4 and ICL2 are colored in bright green. Positions of the membrane boundaries (lipid Cα carbon atoms) are averaged over the trajectory length.

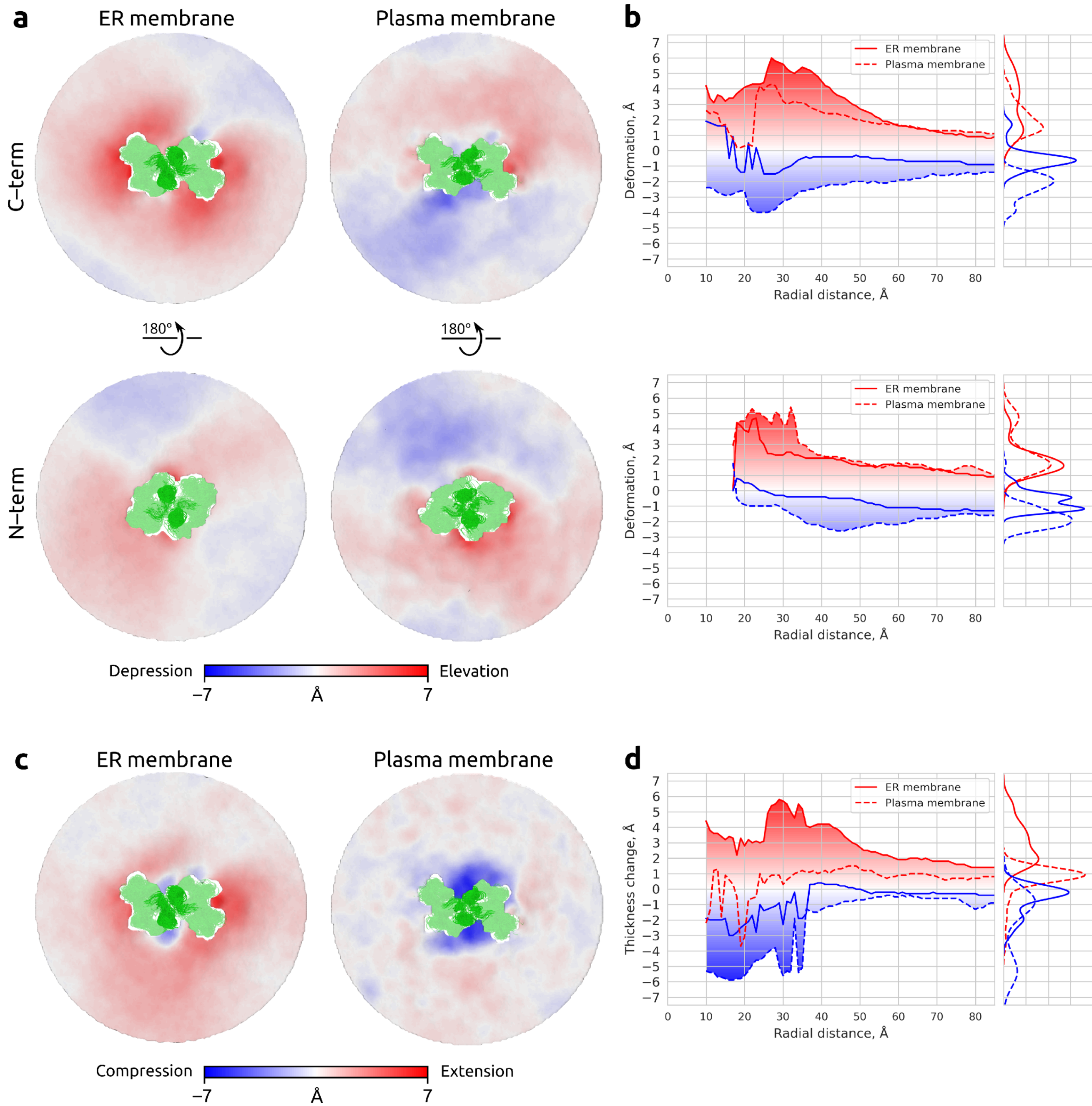

Supplementary Figure 15. Membrane perturbation by the dimeric OLPVR1. a. Membrane deformation around the dimeric OLPVR1. Membrane borders are depicted as surfaces colored red when elevated and blue when depressed. **b**. Extreme deformation values in annuli of width 1 Å around the dimer. The maximum elevation values are colored in red, and the minimum depression values are colored in blue. The range of observed deformation values is colored using a blue-white-red color gradient. Values do not start at zero because there are no lipids in that region. The ER-like membrane is more bent around the OLPVR1. **c**. Thickness changes in the

membranes around the dimeric OLPVR1. Membrane compression and extension are shown in blue and red, respectively. **d**. Extreme values of thickness change in annuli of width 1 Å around the dimer. The maximum extension values are colored in red, and the minimum compression values are colored in blue. The range of observed thickness change values is colored using a blue-white-red color gradient. The ER membrane, and especially the plasma membrane, is thinned near the TMs of 3, 4, and 5. The ER membrane is also thickened near TM1, TM6, and TM7. The OLPVR1 is represented by 20 conformations taken every 50 ns. The TM4 and ICL2 are colored in bright green. Positions of the membrane boundaries (lipid Cα carbon atoms) are averaged over the trajectory length.

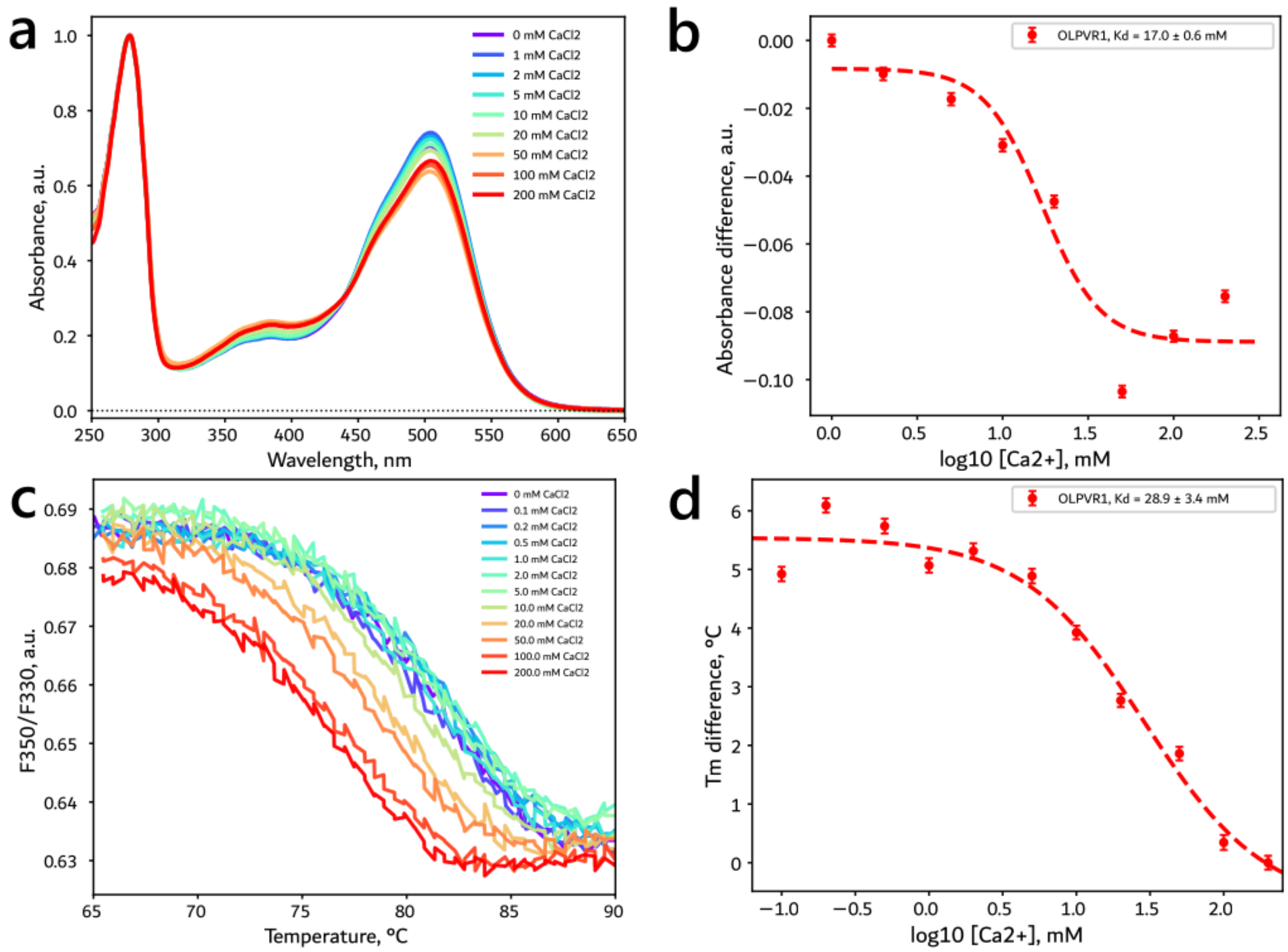

Supplementary Figure 16. Calcium-induced destabilization of the retinal in OLPVR1. a. UV-vis spectra of OLPVR1 at pH 10.2 with different concentrations of Ca^{2+} between 0 and 200 mM. The pH values for all samples were 10.2 ± 0.05. **b**. The sigmoid fit of the difference in absorption change versus calcium concentration. The dissociation constant (K_d) is determined to be 17.0 ± 0.6 mM $CaCl_2$. **c**. Calcium dependence of the unfolding F350/330 curves of OLPVR1

measured with the nanoDSF method. **d**. The sigmoid fit of the thermal unfolding temperature dependence on the concentration of calcium. The K_d of calcium binding was determined to be 28.9 ± 3.4 mM $CaCl_2$.

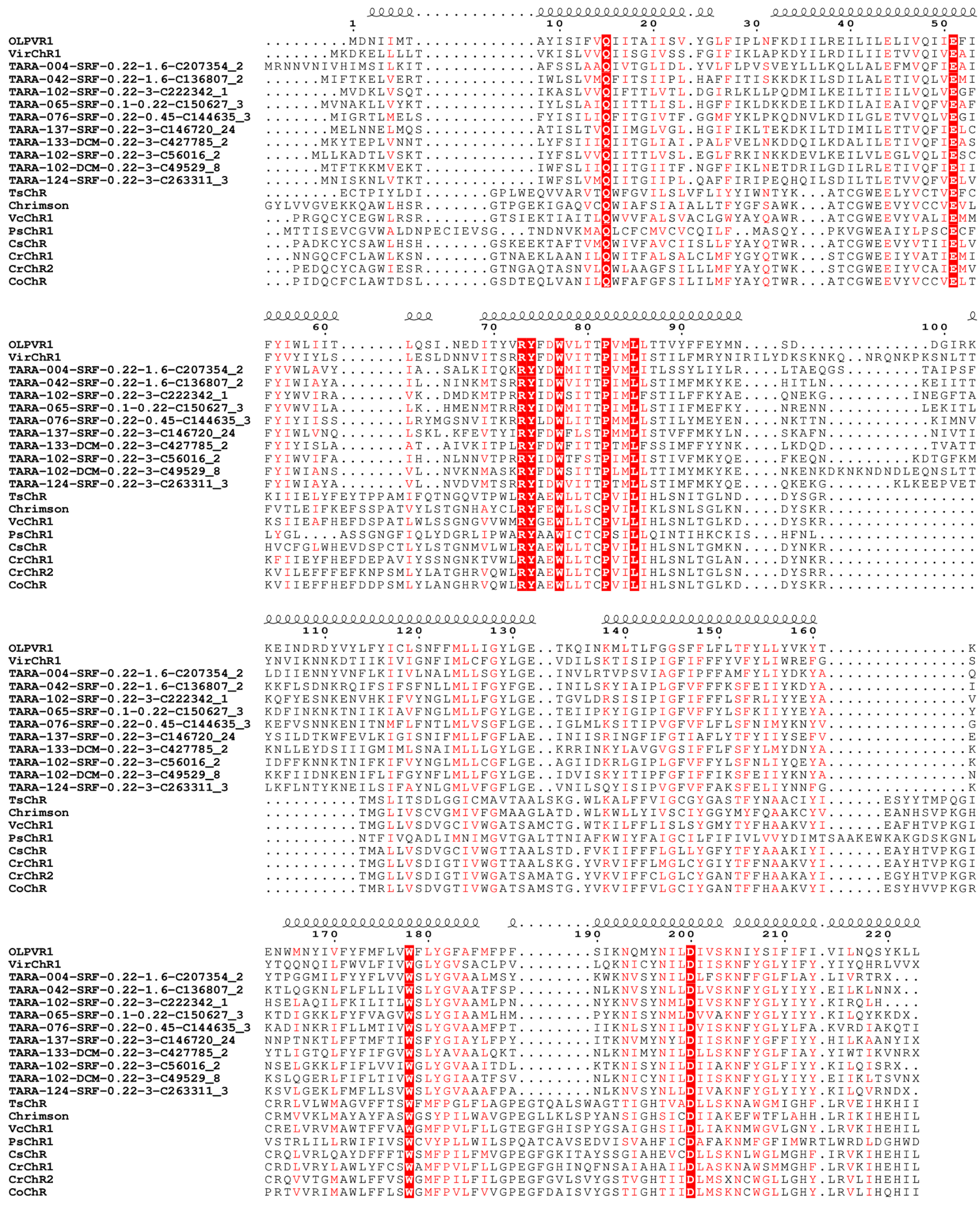

Supplementary Figure 17. Multiple sequence alignment of selected members from VCR1 and CCR families using the Clustal Omega method[20]. The visualization was done using ESPript 3.0 server[21]. The partially and fully conserved residues are shown in red font and red text highlight, respectively.

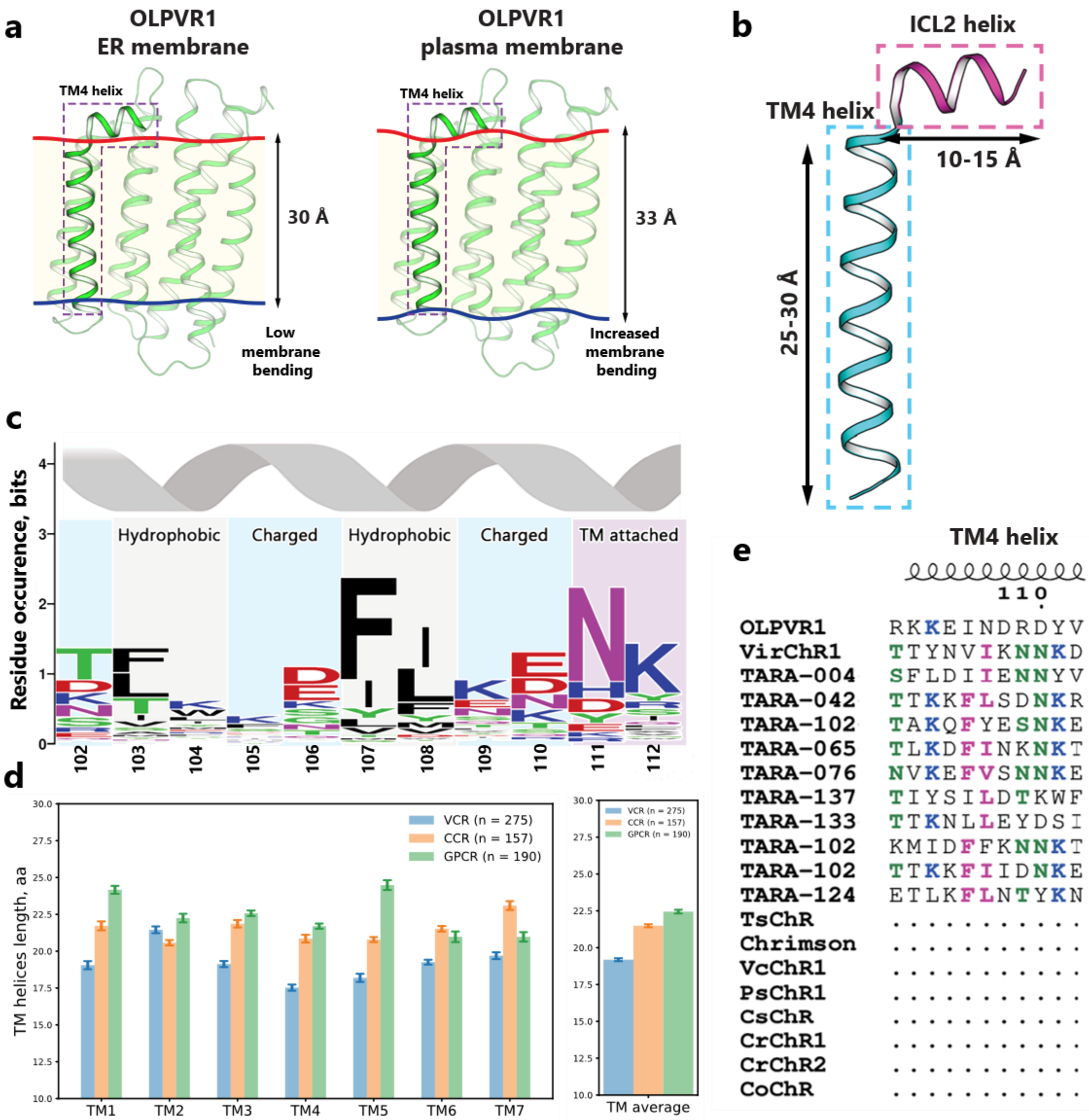

Supplementary Figure 18. Role of TM4 helix in biased localization of VCR1s. a. The schematic model of OLPVR1 adaptation to mammalian ER and plasma membranes and shown on the left and right panels, respectively[4]. The TM4 helix is highlighted with a purple box. **b**. Overview of the TM4 helix composed of the ICL2 juxtamembrane helix domain (pink) and TM4

transmembrane domain (cyan). **c**. The sequence logo was calculated for the ICL2 helix domain for the VCR1 family (n = 275). The repeating hydrophobic and charged regions are indicated. **d**. Comparison of the TM helices length calculated for VCR family (cyan), chlorophyte cation channelrhodopsins (CCR, orange), and GPCRs of type A (green). The prediction was performed with the DeepTMHMM prediction server[22]. The confidence interval for the mean (CI) of 95% is shown for each helix. The Student t-test (*** means p-value < 0.001) was used to compare VCR1 helix length with the CCR and GCPR groups. **e**. Multiple sequence alignment of the ICL2 helix domain for selected VCR and CCR sequences with Clustal Omega[20]. The ICL2 domain was not present in the CCR group. The full sequence alignment is shown in the Supplementary Figure 7.

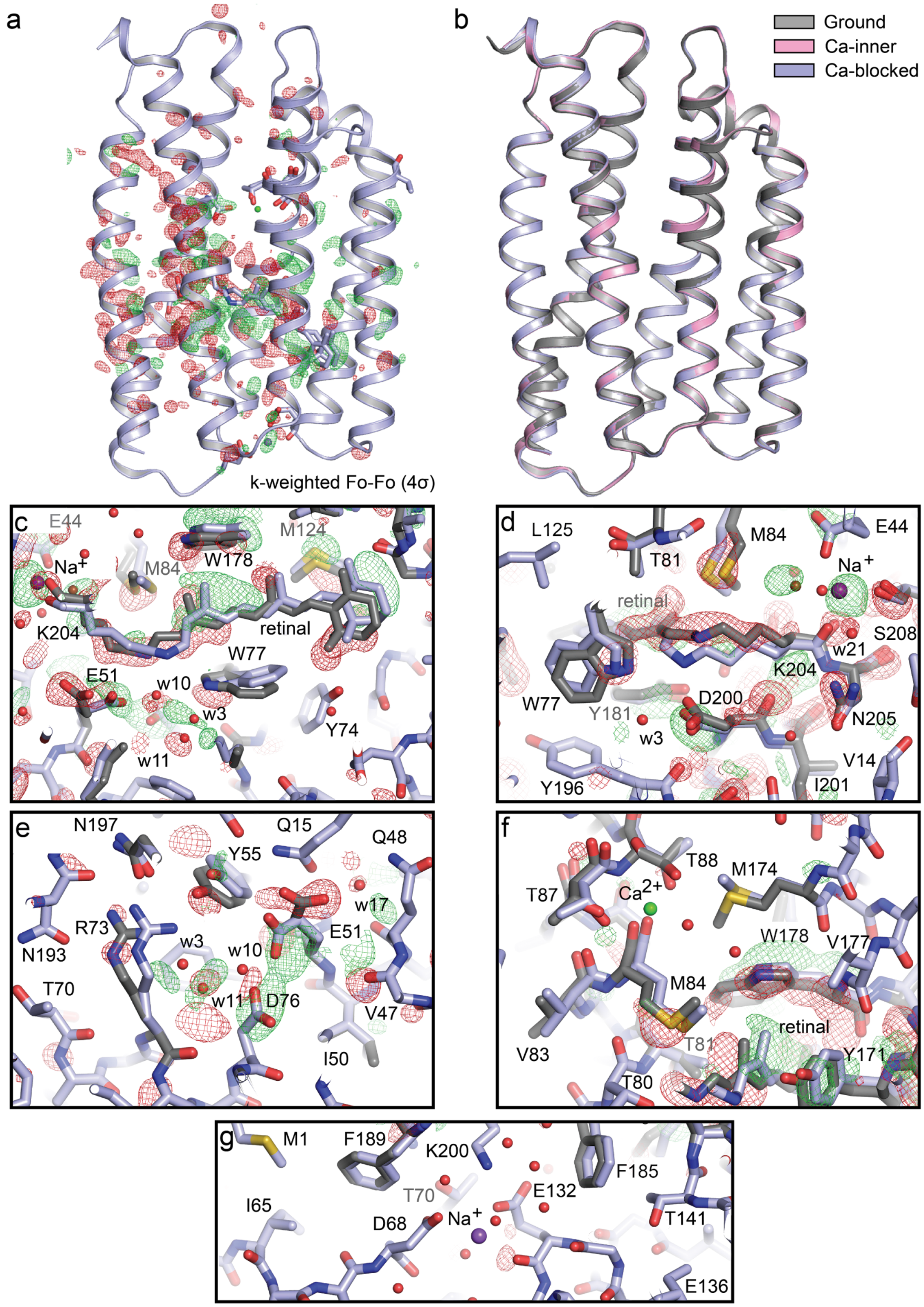
a
b
Ground
Ca-inner
Ca-blocked
k-weighted Fo-Fo (4σ)
c
E44
M124
Na+
M84
W178
K204
retinal
W77
E51
w10
w3
Y74
w11
d
L125
M84
E44
T81
Na+
retinal
S208
w21
K204
W77
D200
Y181
N205
w3
V14
Y196
I201
e
N197
Q15
Q48
Y55
w17
R73
E51
N193
w3
w10
T70
w11
D76
V47
I50
f
T88
M174
T87
Ca2+
W178
V177
M84
retinal
T81
V83
Y171
T80
g
M1
F189
K200
F185
T70
E132
I65
D68
Na+
T141
E136

Supplementary Figure 19. Structure of the Ca-blocked state of OLPVR1. a. Overall structure of OLPVR1 in the Ca-blocked state. **b**. Alignment of OLPVR1 in the ground (gray, PDB ID: 9IN7[7]), Ca-inner (pink, PDB ID: 9SOF), and Ca-blocked (blue, PDB ID: 9SOG) states. **c**. RSB pocket. **d**. Central and Cytoplasmic gates. **e**. Extracellular gate. **f**. Ca-binding site A. **g**. Na-entry site. **c** to **g**. Non-photoactivated Ca-inner state (conf A, colored gray, occ=70%) and Ca-blocked state (conf B, colored blue, occ=30%) are depicted with sticks. $Fo^{Ca\text{-}blocked}$-$Fo^{Ca\text{-}inner}$ k-weighted TV-denoised isomorphous difference map built with Meteor[23] is contoured at a 4σ-level.

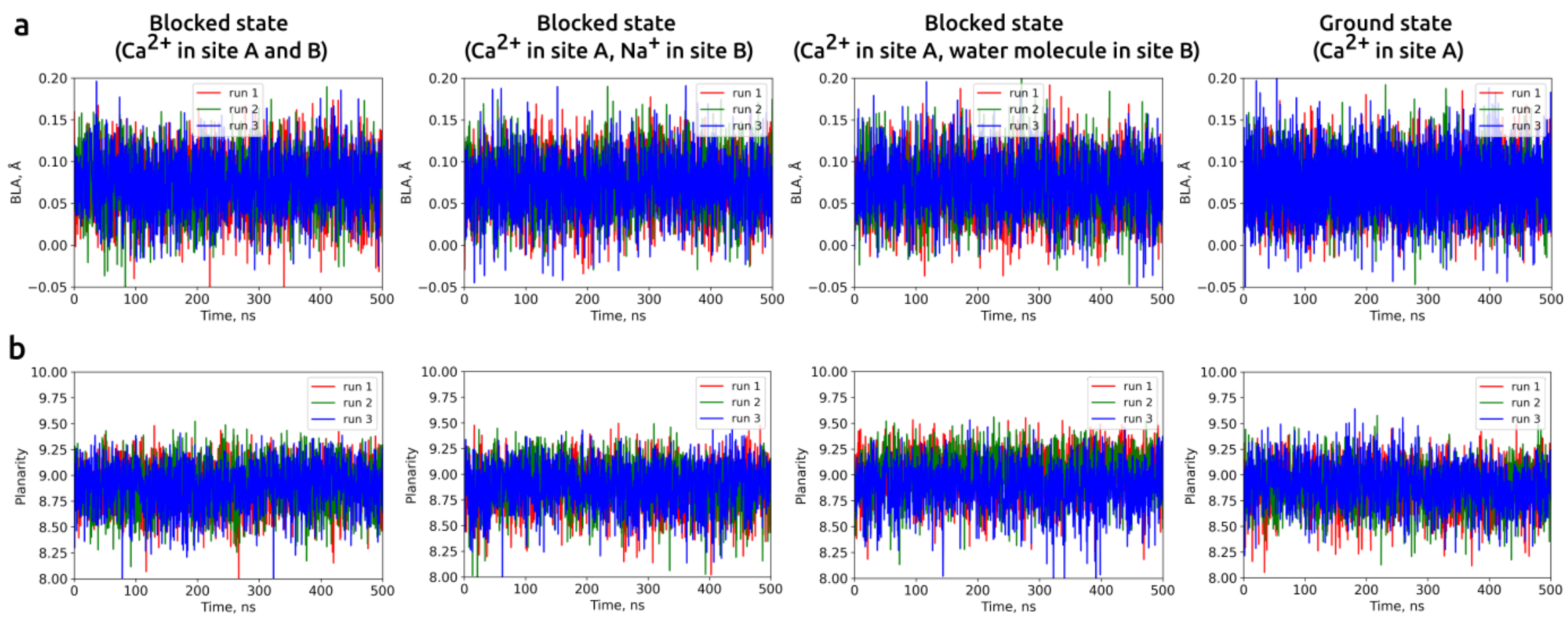

Supplementary Figure 20. Retinal dynamics in molecular dynamics simulations of OLPVR1. a. The bond-length alternation (BLA) of 13-cis (blocked state) and all-trans (ground state) retinal for 500 ns simulations. **b.** The planarity of 13-cis (blocked state) and all-trans (ground state) retinal during 500-ns simulations. In all simulations, the retinal maintains its starting structure for 500 ns. For each case, three replicas of the simulation were performed. These structural parameters were calculated using the equations from[24].

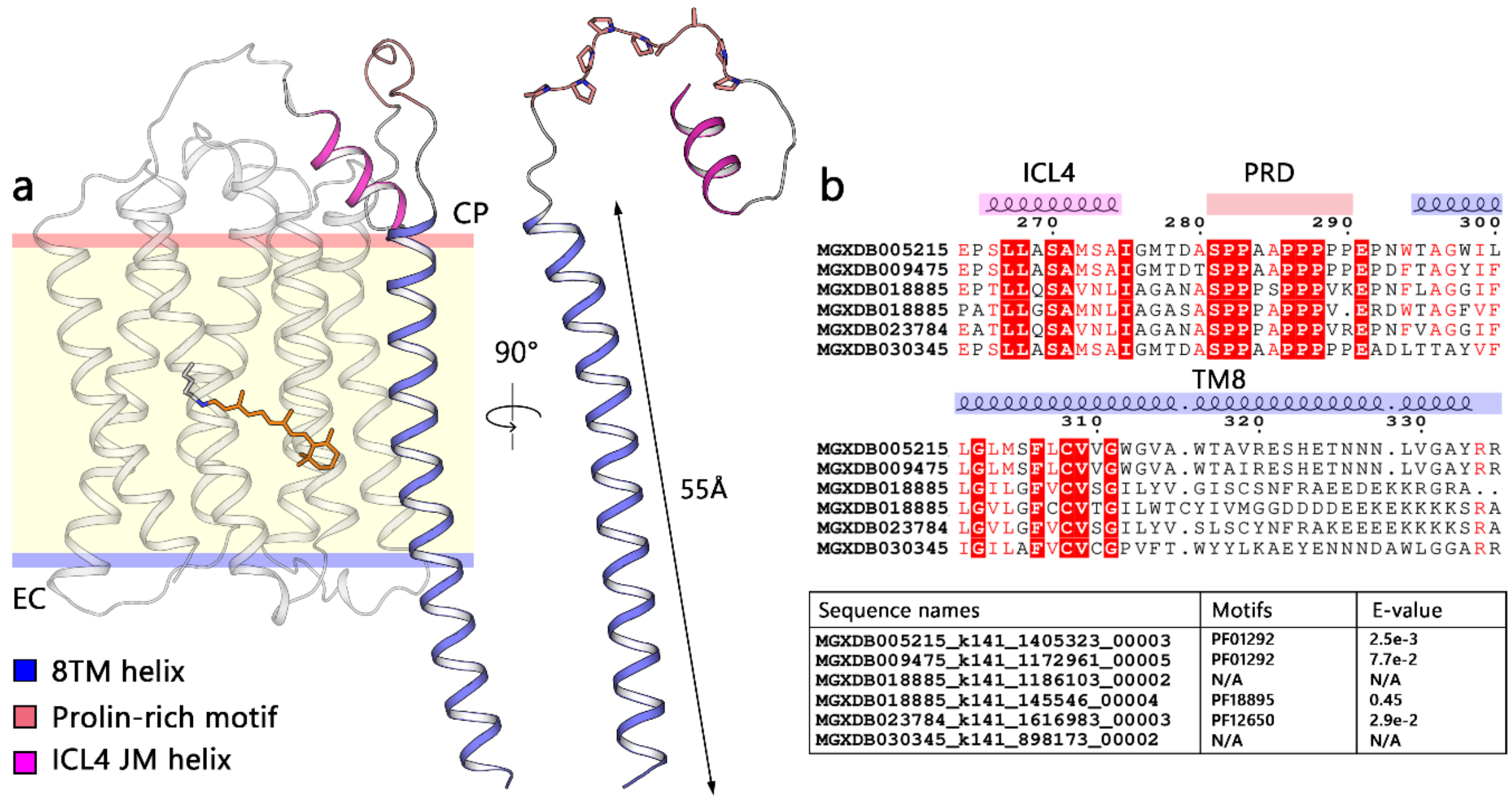

Sequence names	Motifs	E-value
MGXDB005215_k141_1405323_00003	PF01292	2.5e-3
MGXDB009475_k141_1172961_00005	PF01292	7.7e-2
MGXDB018885_k141_1186103_00002	N/A	N/A
MGXDB018885_k141_145546_00004	PF18895	0.45
MGXDB023784_k141_1616983_00003	PF12650	2.9e-2
MGXDB030345_k141_898173_00002	N/A	N/A

Supplementary Figure 21. VCR1 8TM proteins. a. AlphaFold-generated (CollabFold 1.6.1) model of an 8TM VCR rhodopsin (id: MGXDB005215_k141_1405323_00003). The membrane boundaries are indicated. The 8TM-associated domains, 8TM helix, proline-rich domain (PRD), and ICL4 helix are colored blue, salmon, and purple, respectively. b. Sequence alignment of the 8TM domains. The partially and fully conserved residues are shown in red font color and red text highlight color, respectively. Identified sequence motifs in the 8TM domains and their respective PFAM codes and e-values are shown on the bottom panel[25]. The motif search was done with the MotifSearch tool (https://www.genome.jp/tools/motif).
