# Supplementary material for: Molecular mechanism of calcium inhibition in viral channelrhodopsins
Source: Nat Commun. 2026 Sep 16;17:9875. doi: 10.1038/s41467-026-77716-5 (PMC13582892; doi:10.1038/s41467-026-77716-5)
Supplement: Supplementary file 2 [file 41467_2026_77716_MOESM2_ESM.pdf]

Title: Supplementary Data 1

Description: Genetic Constructs used in this study
