# Supplementary material for: Molecular mechanism of calcium inhibition in viral channelrhodopsins
Source: Nat Commun. 2026 Sep 16;17:9875. doi: 10.1038/s41467-026-77716-5 (PMC13582892; doi:10.1038/s41467-026-77716-5)
Supplement: Supplementary file 4 [file 41467_2026_77716_MOESM5_ESM.pdf]

nature portfolio

Peer Review File

Molecular mechanism of calcium inhibition in viral channelrhodopsins

Corresponding Author: Dr Dmitrii Zabelskii

This file contains all reviewer reports in order by version, followed by all author rebuttals in order by version.

Version 0:

Reviewer comments:

Reviewer #1

(Remarks to the Author)
In this manuscript the authors present new structural models of the viral channelrhodopsin OLPVR1 obtained by X-ray crystallography. They used Ca2+ soaking experiments and found Ca2+ binding sites, one of them leading to a blocked state.
The strength of this manuscript is that the structural models are extensively validated by several methods, including time-resolved UV/Vis spectroscopy including ultrafast measurement, FTIR-difference spectroscopy, MD simulations and atomic force measurements. In general, I can follow the authors' reasoning, and most of the results are supported by the data. However, some parts of the manuscript are not well integrated into the manuscript. The AFM experiments do not appear at all in the manuscript, and the part in the supplement is not well integrated with the rest of the manuscript. The MD simulations are not adequately described. Supplementary Figures 3, 4 & 5 are not mentioned in the main text.
Questions regarding the MD simulations:
Why are different box sizes used for different membranes? It seems that for the calculations in the POPC membrane, the complete backbone was restrained, but in the other membranes not. Why is that the case? Which simulation system is shown in Figure ED7? Would backbone restriction change the result significantly?
Monomer and dimers were simulated. What is the influence of the dimer?
For reporting on stable and unstable ion positions, I am missing a proper statistic. It is state of the art to perform at least three replicas. Further it is not clear which of the simulation system was used for the main statement.
How were the positions chosen and how the arrow in Figure ED7? Are there significant changes to the side chains as well? Please indicate which X-ray structures (pdb IDs) were used for each simulation.

Questions regarding UV/Vis-spectroscopy:
The description of ultrafast transient absorption is missing in the Methods section.
The lack of consistency between the ultrafast and flash photolysis experiments could also be due to the different excitation light source. An ultrashort pulse could lead to two photon processes and longer pulses could lead to excitation of the first intermediate or back reaction. This should be discussed.
While the LDM method used is state of the art, it would be nice to see multiexponential fits with residuals to evaluate the quality of the fits.

The FTIR data are of excellent quality, and the method is nicely illustrated in Figure ED3.
In the FTIR spectra shown in the extended data, most labels are missing
In Figure 4 c, it seems that absorbance differences are plotted. This should be displayed correctly: "-" sign instead of "~" or "/". The same in 4d "-" instead of "/".
For band assignment, it would be nice to measure mutants, but that is beyond the scope here.
In this part in noticed some typos: cm-1 superscript "-1" missing
a-helix (a should be Greek letter)
Are the protonation states of E44 and E51 in the MD simulation according to the FTIR results?
A double difference of the light and dark spectra in Figure 4b seems to reveal a band at 1748 cm-1. This should be discussed.

Page 7, line 92, "Data"
Page 12, line 16 should be "sub-micromolar"

Reviewer #2

(Remarks to the Author)
The manuscript describes a study of OLPVR1, a representative of viral channelrhodopsins, VCR1s, focusing on the molecular mechanism of Na+ permeation and Ca2+ inhibition. The study combines several approaches, including high-resolution X-ray crystallography, time-resolved UV-Vis flash photolysis, ATR-FTIR spectroscopy, thermal stability measurements, molecular dynamics simulations, and high-speed AFM, which together shed new light on the OLPVR1 ion conductance mechanisms. The authors hypothesize that VCR1s follow the model of single-file ion conductance earlier proposed for K+ channels. The results of this manuscript improve our understanding of the molecular mechanisms of VCR1 action, providing new insights into virus-host interactions and foundations for optogenetic tool engineering. Overall, the experiments appear to be of high quality, the manuscript is written clearly, and the results are sound and important. I only have a few comments aiming to help with further improvement of this excellent manuscript.
In K+ channels, the single-file mechanism of K+ permeation is illustrated by structures, where each binding site is represented by ion density. In the case of OLPVR1, positions of only two Na+ ions are visualized throughout the entire putative conductance pathway, substantially reducing the visualization power and confidence in the potential single-file mechanism of Na+ permeation. It would be nice to include a discussion of current limitations of the structural approach and outline other possible mechanisms of ion permeation.
Other comments:
1. Page 4, first sentence of Results. Calcium-bound structures were obtained through soaking crystals with calcium chloride. Did authors try to crystallize OLPVR1 in the presence of Ca2+, where they can control calcium concentration much better and where they may capture a somewhat different protein conformation or reveal different ion binding sites?
2. Page 10, line 7. Should “nearly covalent 2.1A H-bond” be replaced with “strong 2.1A H-bond”?
3. Figure 1. The box surrounding the inner Ca2+ binding site is colored the same way as the box surrounding the Ca2+ surface site, while only the latter one is shown in magnified view. To avoid confusion, please change the color of the box surrounding the inner Ca2+ binding site.
4. Figure 4c. Are there error bars for the absorbance measurements? If the points represent single measurements, should the measurements be repeated to be statistically reliable at each concentration? For the 1680-cm-1 band, is it possible to extend the concentration range of Ca2+ into high concentrations to estimate the Kd value?
5. Figure 5. Please fix typo on line 4 of the legend.
6. Figure 6. Should the upper Na+ binding site be shown on the left two panels with the dashed contour?

Reviewer #3

(Remarks to the Author)
This manuscript investigates the calcium and sodium binding in the OLPVR1 ion channel. These results appear significant and novel. The work includes a large experimental component, on which I can only comment in a limited way. However, I can provide more detailed feedback on the computational part. Overall, the MD simulations could yield substantially more quantitative information that would better support the authors' initial hypotheses and conclusions.

Comment 1: The authors have performed a 500 ns molecular dynamics simulation to examine water, calcium, and sodium binding sites. Several situations have been analyzed as shown in Figure 7. However, no statistical data are reported to support the stated conclusions. For example, the authors write "the water molecule quickly leaves site B" but this claim should be quantified. It would be very informative to:

1. Plot, as a function of time (over the last 200 ns of the production trajectory), the number of calcium ions, sodium ions, and water molecules occupying the binding site(s).

2. Compute and plot the residence time for calcium ions, sodium ions, and water molecules using the last 200 ns of the production trajectory. For example, the residence time of calcium and sodium when E44 is deprotonated and E51 is protonated.

Both analyses would strongly support the claim that the ions are indeed stable in the binding site.

Comment 2: Regarding calcium ion blocking the channeling pathway. Several conformations of the retinal cofactor are shown in Fig. 2. The caption reads "different structures". Is the retinal in the same isomeric state across all snapshots examined? Again, highly useful statistical information can be extracted from the MD trajectory.
It would be particularly insightful if the authors do compute retinal dihedral angles and bond-length alternation over time (as they do here: https://doi.org/10.1021/acs.jctc.5c01719) for the last 200 ns of the production trajectory, and discuss how the chromophore conformation is influenced by the surrounding residues.

Version 1:

Reviewer comments:

Reviewer #1

(Remarks to the Author)
The authors conducted a thorough revision, including additional MD simulations, as requested by two of the reviewers. They have satisfactorily addressed all of my points.

Reviewer #2

(Remarks to the Author)
The authors addressed my previous critiques and I have no further comments.

Reviewer #3

(Remarks to the Author)
The authors have addressed most of my concerns. There is only one remaining point that needs to be fixed.

Specifically, the added statement in the main manuscript:

“Retinal remained stable in the 13-cis conformation for the Ca-blocked state simulations,”

is somewhat vague, as the term “stable” is ambiguous.

The authors should specify what they mean by stability and indicate the structural parameters on which this conclusion is based. In this case, those parameters are bond length alternation (BLA) and planarity, for which different definitions can be found in the literature for the retinal Schiff base molecule. Since the authors have adopted a specific definition of BLA and planarity, I recommend supporting this claim with a statement such as:

“We analyzed planarity and bond length alternation as defined in Ref. [10.1021/acs.jctc.5c01719], and observed that retinal remained in the 13-cis conformation in the Ca-blocked state simulations.”

Version 2:

Reviewer comments:

Reviewer #3

(Remarks to the Author)
The authors have addressed all my comments.

Open Access This Peer Review File is licensed under a Creative Commons Attribution 4.0 International License, which permits use, sharing, adaptation, distribution and reproduction in any medium or format, as long as you give appropriate credit to the original author(s) and the source, provide a link to the Creative Commons license, and indicate if changes were

made.
In cases where reviewers are anonymous, credit should be given to 'Anonymous Referee' and the source.
The images or other third party material in this Peer Review File are included in the article's Creative Commons license, unless indicated otherwise in a credit line to the material. If material is not included in the article's Creative Commons license and your intended use is not permitted by statutory regulation or exceeds the permitted use, you will need to obtain permission directly from the copyright holder.
To view a copy of this license, visit https://creativecommons.org/licenses/by/4.0/

REVIEWER COMMENTS

First, we thank the Reviewers for their constructive comments, which prompted us to carefully revisit the manuscript and improve several key sections. In response to their suggestions, we repeated the molecular modeling experiments using three replicas and standardized conditions across all runs. We also added multiple new figures and revised several figures already included in the manuscript. In addition, the manuscript has been additionally reviewed for grammatical and stylistic accuracy.

We hope that the revised manuscript is now suitable for publication in *Nature Communications*. Our detailed responses to the reviewers' specific comments are provided below.

Reviewer #1 (Remarks to the Author):

In this manuscript the authors present new structural models of the viral channelrhodopsin OLPVR1 obtained by X-ray crystallography. They used Ca2+ soaking experiments and found Ca2+ binding sites, one of them leading to a blocked state.

The strength of this manuscript is that the structural models are extensively validated by several methods, including time-resolved UV/Vis spectroscopy including ultrafast measurement, FTIR-difference spectroscopy, MD simulations and atomic force measurements. In general, I can follow the authors' reasoning, and most of the results are supported by the data.

However, some parts of the manuscript are not well integrated into the manuscript. The AFM experiments do not appear at all in the manuscript, and the part in the supplement is not well integrated with the rest of the manuscript.

The MD simulations are not adequately described. Supplementary Figures 3, 4 & 5 are not mentioned in the main text.

Taking into account this comment of the reviewer, the Supplementary Figures 3,4 and 5, as well as other Figures, are now referenced in the main text, except for Supplementary Figure 11, which is referenced in the Supplementary Note.

Questions regarding the MD simulations:

- ***Why are different box sizes used for different membranes? It seems that for the calculations in the POPC membrane, the complete backbone was restrained, but in the other membranes not. Why is that the case? Which simulation system is shown in Figure ED7? Would backbone restriction change the result significantly?***

We thank the Reviewer for the important clarifying questions. When studying the interaction of OLPVR1 with lipid environments that mimic the plasma membrane and the endoplasmic reticulum, we used boxes with larger XY dimensions (18x18 nm^2) than for POPC membranes (9x9 nm^2) to avoid potential artifacts arising from periodic boundary conditions. Analysis of the membrane deformation induced by the protein showed that the chosen box sizes allow the deformation to decay with distance from the protein (Supplementary Figures 5 and 6 in the revised manuscript). To study the dynamics of ions within OLPVR1, we performed monomer simulations with harmonic backbone restraints in the POPC bilayer, and the results are shown in Extended Data Figure 8. Harmonic restraints on the monomer backbone were applied to focus on the interaction between side chains of important gating residues and ions, and to avoid any artifacts caused by the helical perturbations. It is important to note that all structures were collected at cryo temperatures (100K), whereas the modelling is performed at room temperature (297K), thereby increasing thermal perturbations. This approach was

previously applied to the study of ion stability in other rhodopsins, such as the anion pump SyHR[1] (doi.org/10.1038/s41467-022-34019-9). Also, some interactions might not be properly modelled by classical force fields (for example, the interactions among E51, D76, D200, and RSB, which suggest proton delocalization). Thus, in this case, we believe that the use of restraints enabled us to more accurately reproduce the protein's behaviour in a particular photointermediate state.

- ***Monomer and dimers were simulated. What is the influence of the dimer?***

In this manuscript, we discuss the oligomeric state of OLPVR1 in solution; however, due to the text limits, the corresponding discussion was moved to the supplementary data, and we agree with the Reviewer that this question warrants a more thorough discussion. The specific answer to this question requires additional context, which has been added in the updated manuscript.

Generally, microbial rhodopsins (MRs) exist in cells in a specific oligomeric, rather than monomeric state, which is also linked to their evolutionary origin[2]. Typically, MRs form trimers, pentamers, or hexamers, and dimers. However, crystallization conditions can significantly alter the native oligomeric state of MRs, thereby changing kinetic rates, as reported for OLPVR1 in this manuscript. In some cases, disruption of the oligomeric state can result in significant changes in protein function. For instance, for the sodium pump KR2, different sodium-binding sites were reported for monomer and pentamer forms of the protein, and the monomeric form was hypothesized to be inactive due to its tilt in the membrane [3–5].

In the case of OLPVR1, the nearest studied homologue is the OLPVRII protein from viral rhodopsin group 2, which forms a tightly bound pentamer in both solution and crystals[6]. In contrast, OLPVR1 lacks nearly all residues involved in pentamer formation and forms a loose dimer in crystals, which is unlikely to reflect the protein's native oligomeric state. We have previously suggested that OLPVR1 is likely to be a dimer in solution and reported a possible 'tight' dimer configuration[7]; however, in this work, we performed an atomic force microscopy experiment that directly confirmed that OLPVR1 exists as a dimer in solution. Therefore, to assess whether the dimer state influences the protein function, we performed molecular modelling. As discussed in the manuscript, we did not observe any major discrepancies and thus believe that the oligomeric state does not visibly affect protein function in OLPVR1.

This discussion is now added to the Results section in the section **'OLPVR1 forms a dimer in the membrane'.**

- ***For reporting on stable and unstable ion positions, I am missing a proper statistic. It is state of the art to perform at least three replicas. Further it is not clear which of the simulation system was used for the main statement.***

We agree with the Reviewer that three replicas should be performed. We re-run the corresponding molecular modelling experiments to ensure that all runs are fully compatible. We performed three replicas of the OLPVR1 simulation for the blocked state (three cases: Ca, Na, and a water molecule at site B) and for the illuminated ground state. The results are shown in the updated Extended Data Figure 8. We have also attached the updated trajectories to the revision files in case the Reviewers would like to inspect them.

- ***How were the positions chosen and how the arrow in Figure ED7? Are there significant changes to the side chains as well?***

We selected the positions of sites (site A and site B) as the likely ion-binding sites from the crystallographic data. The positions of selected residues are chosen based on their proximity to the

ions and potential hydrogen-bonding interactions with key water molecules in the intracellular gate (see Figure 2). The arrow indicates the time-dependent displacement of the calcium ion in the model (with two calcium atoms). This effect is persistent across simulations and present in three replicas of the updated trajectories.

The displacements of the side chains are presented in Supplementary Figure 4 of the updated version of the manuscript. We added an extended discussion of the results to the Supplementary note in the section '**Side-chain fluctuations during the molecular modelling experiments**'.

- ***Please indicate which X-ray structures (pdb IDs) were used for each simulation.***

We thank the Reviewer for this request for clarification, which we believe will enhance the manuscript's clarity and benefit its readers. PDB ID 9SOF was used to perform MD simulations of the monomeric OLPVR1 (with restraints) in the calcium-blocked state. PDB ID 9IN7 was used to perform MD simulations of the monomeric OLPVR1 (with no restraints) in the ground state. The structural model obtained by homology modelling was used to perform MD simulations of the dimeric OLPVR1 (with no restraints) in the ground state. The model of 'tight' OLPVR1 dimer was calculated via Galaxy Homomer server[8].

Questions regarding UV/Vis-spectroscopy:

The description of ultrafast transient absorption is missing in the Methods section.

We apologize for overlooking this important detail during manuscript preparation. Accordingly, we added an appropriate methods section for ultrafast spectroscopy and revised the methods section for flash photolysis spectroscopy, as the data were obtained in solution rather than *in crystallo*. Lastly, we added a new section describing the pump-probe setup consisting of two synchronized fs laser systems used to bridge the time gap on the ns timescale. This measurement reveals spectral evolution on the ns timescale and explains the "inconsistency" between the ultrafast and flash-photolysis timescales noted in the original version of the manuscript.

The lack of consistency between the ultrafast and flash photolysis experiments could also be due to the different excitation light source. An ultrashort pulse could lead to two photon processes and longer pulses could lead to excitation of the first intermediate or back reaction. This should be discussed.

We agree with the reviewer that differences between the end of the ultrafast regime and the beginning of the flash photolysis measurement could be due to setup-specific parameters. Yet, we want to point out that the points mentioned above are highly unlikely in our experiments, as we use comparably low pulse powers when measuring microbial rhodopsins in our lab. Under the same experimental conditions, we have repeatedly observed that different parts of the respective measurements are consistent (e.g., https://doi.org/10.1021/acs.jpclett.4c00693, https://doi.org/10.1038/s41467-024-47469-0). In an experiment with two synchronized fs-lasers, we closed the experimental time gap of the data provided in the original version of this manuscript (end of ultrafast timescale: 1.8 ns; beginning of flash photolysis timescale: 670 ns), we could show that the lack of consistency of the presented measurements is caused by kinetic processes occurring during this time gap (Supplementary Figure 1): We chose pH 8, 0 mM NaCl, 0 mM CaCl2 as the representative condition as it has the shortest photocycle duration among all conditions considered. Furthermore, we increased the pump energy from 90 nJ/pulse to 200 nJ/pulse to improve the S/N ratio, as signals are comparably small in this setup. The results of this additional experiment are shown in Supplementary Figure 1. As can be seen, the start of the measurement with the synchronized laser

setup (1.2 ns) exhibits the same signatures as the original ultrafast measurement. Furthermore, the end of the additional measurement shows identical signatures for the early microseconds as observed in the flash photolysis measurement. In addition, this dataset shows a kinetic component at around 100 ns, which can be associated with the P1-to-P2 transition. This further supports our previous interpretation that the inconsistency between the ultrafast and flash photolysis experiments arises from a transition occurring during the experimental time gap.

While the LDM method used is state of the art, it would be nice to see multiexponential fits with residuals to evaluate the quality of the fits.

We appreciate the Reviewer's comment that the LDM method is state-of-the-art; nevertheless, multiexponential analysis is more commonly applied and sometimes facilitates the evaluation of the provided fitting. Accordingly, we have employed global lifetime analysis to perform multiexponential fitting. The results are provided in Supplementary Figure 2. First, we show the same transients as in the LDM analysis (340 nm, 440 nm, 480 nm, and 560 nm), together with the corresponding fits. Secondly, we show the decay-associated spectra (DAS) together with the obtained lifetimes and, as requested by the reviewer, the fitting residuals. At this point, we want to stress that the flash photolysis technique is intrinsically more susceptible to noise at early times (ns-µs), leading to comparably larger residuals in this time window.

In the FTIR spectra shown in the extended data, most labels are missing

In Extended Data Figure 4, we labeled all frequencies in the absence of Ca (Top panel), while we labeled the frequencies in the presence of Ca (Bottom panel) that differ from those in the absence. We believe that such a presentation is helpful to readers, who can easily recognize whether vibrational bands are identical or different.

To make this point clear, we revised the manuscript as follows.

"… In the presence of $CaCl_2$ (Bottom), the frequencies that differ from those in the absence are only labeled." (Extended Data Figure 4 Legend)

In Figure 4 c, it seems that absorbance differences are plotted. This should be displayed correctly: "-" sign instead of "~" or "/". The same in 4d "-" instead of "/".

The sign was somehow complicated, and we have to agree that it is misleading. In Fig. 4c, Left is the intensity for the band at 1717 cm-1 that was obtained by the average between 1712 and 1711 cm-1; Middle is the intensity at 1680 cm-1 (single frequency); Right is the intensity difference between the positive 1657-cm-1 and negative 1666-cm-1 bands.

To clearly show this point, we changed the figure as you suggest and revised the manuscript as follows.

"… c. Typical IR band intensities at 1717 cm-1 (C=O stretch of protonated carboxylic acids), 1680 cm-1, and 1657-1666 cm-1 (amide-I of ?-helix) against Ca2+ concentration. The difference between light and dark was plotted at 1712~1722 cm-1 and 1680 cm-1. On the other hand, the difference of the 1657 (+)-cm-1 minus 1666 (-)-cm-1 bands was plotted for both dark (dotted line) and light (red circles). Solid lines were obtained from curve fitting using the Hill equation. While Kd values were not estimated precisely for the 1680-cm-1 band (Kd > 50 mM), Kd values were determined to be 1.1 mM (1717 cm-1) and …" (Figure 4 Legend)

For band assignment, it would be nice to measure mutants, but that is beyond the scope here.

Thank you for your important comment.

After the submission of this paper, Kandori group published two papers by low-temperature FTIR spectroscopy at 77 K; one on OLPVR1[9] (Aoyama et al. Biophys. Physicobiol. 2025) and another on OLPVR2[10] (Aoyama et al. J. Phys. Chem. B 2026). In the former paper, the 1722-cm-1 band of OLPVR1 was assigned to the C=O stretching vibration of protonated E51. Although the vibrational frequency of E44 was not assigned in the former paper, the latter paper reported a carboxylic C=O stretch at 1714 cm-1 in OLPVR2 that lacks E51 but possesses E44 (E42 in OLPVR2). As the microenvironments are similar between E44 in OLPVR1 and E42 in OLPVR2, E44 is possibly protonated in OLPVR1, with its C=O stretch near 1714 cm^{-1}. These papers are cited in the updated version of the manuscript.

We also performed ATR-FTIR measurements on E44Q and E51Q at room temperature after submitting this paper. Unfortunately, the C=O stretch region in the light-minus-dark spectra (Extended Data Figure 3b) was too noisy to identify vibrations. On the other hand, we found no Ca-binding to E44Q and E51Q (Extended Data Figure 5), indicating their important roles.

In the revised manuscript, we added a new figure (Extended Data Figure 5) and revised the manuscript as follows. Thank you.

"… in Figure 4b (1717 cm^{-1}). A recent difference FTIR study of OLPVR1 mutants at 77 K identified the 1722 cm^{-1} band as the C=O stretch of protonated E51[9] (Aoyama et al. 2025). Although the vibrational frequency of E44 was not assigned in the paper, a difference FTIR study of OLPVR2 at 77 K reported a carboxylic C=O stretch at 1714 cm-1[10] (Aoyama et al. 2026). OLPVR2 lacks E51 but possesses E44 (E42 in OLPVR2), and because of similar microenvironments between E44 in OLPVR1 and E42 in OLPVR2, E44 is probably protonated in OLPVR1, whose C=O stretch is located near 1714 cm^{-1}. We then applied presence-minus-absence difference FTIR spectroscopy to E44Q and E51Q at various Ca2+ concentrations using the flow system. Unlike the wild-type, both E44Q and E51Q exhibited no Ca2+ binding even under light conditions (Extended Data Figure 5), indicating important roles of E44 and E51 for Ca^{2+} binding."

In this part in noticed some typos: cm-1 superscript "-1" missing

We revised accordingly, thank you.

a-helix (a should be Greek letter)

We revised accordingly, thank you.

A double difference of the light and dark spectra in Figure 4b seems to reveal a band at 1748 cm-1. This should be discussed.

According to your suggestion, we labelled a positive peak at 1741 cm^{-1} in Figure 4b, and revised the manuscript as follows.

"… between dark and light, where a negative peak at 1717 cm^{-1} together with a small positive peak at 1741 cm^{-1} indicates perturbation of a carboxylate. From the titration curve …"

Reviewer #2 (Remarks to the Author):

The manuscript describes a study of OLPVR1, a representative of viral channelrhodopsins, VCR1s, focusing on the molecular mechanism of Na+ permeation and Ca2+ inhibition. The study combines several approaches, including high-resolution X-ray crystallography, time-resolved UV-Vis flash

photolysis, ATR-FTIR spectroscopy, thermal stability measurements, molecular dynamics simulations, and high-speed AFM, which together shed new light on the OLPVR1 ion conductance mechanisms. The authors hypothesize that VCR1s follow the model of single-file ion conductance earlier proposed for K+ channels. The results of this manuscript improve our understanding of the molecular mechanisms of VCR1 action, providing new insights into virus-host interactions and foundations for optogenetic tool engineering. Overall, the experiments appear to be of high quality, the manuscript is written clearly, and the results are sound and important. I only have a few comments aiming to help with further improvement of this excellent manuscript.

In K+ channels, the single-file mechanism of K+ permeation is illustrated by structures, where each binding site is represented by ion density. In the case of OLPVR1, positions of only two Na+ ions are visualized throughout the entire putative conductance pathway, substantially reducing the visualization power and confidence in the potential single-file mechanism of Na+ permeation. It would be nice to include a discussion of current limitations of the structural approach and outline other possible mechanisms of ion permeation.

We thank the Reviewer for this important point. We agree that, compared with K^+ channels, in which multiple discrete ion densities highlight each binding site, our crystallographic snapshots provide more limited direct visualization of Na^+ occupancy along the full permeation pathway. In OLPVR1, Na^+ is clearly observed at the extracellular entry site in the ground state, while the open state does not show stable ion-associated density, and an additional Na^+ position (site B) becomes detectable only in the Ca-blocked state due to the Ca^{2+}-induced constriction (together with the persistent entry Na^+). It is also important to note that the updated modelling results indicate that site B can accommodate a water molecule, which remains stable at the site over the modelling time (500 ns). Nevertheless, we believe that site B is likely occupied by the Na^+ ion, for two reasons:

1. The reported structures were obtained in sodium-saturated conditions of 900 mM NaCl, where the sodium ions are abundant.
2. In Site B, the proposed sodium ion is coordinated by the E44-S208-K204 triad (see Figure 2d) with three hydrogen bonds, each with a length of 2.2 Å. The typical hydrogen-bond lengths for water and sodium ions are 2.7–3.0 Å and 2.2–2.4 Å, respectively. Thus, under the cryo conditions (100 K).

These arguments have been added to the updated manuscript.

We consider our structural data as points towards a "single-file-like" permeation scenario, but we acknowledge that static crystallography alone cannot fully resolve the complete multi-ion permeation cycle, especially for highly mobile, partially hydrated monovalent ions and for photoactivated intermediates with partial occupancy. Importantly, the absence of Na^+ density in the open state should not be interpreted as the absence of ions in the conductive pore, but rather as reflecting transient, weakly populated, and rapidly exchanging ion positions that are challenging to capture structurally.

Following the Reviewer's suggestion, we have expanded the Discussion to explicitly describe the limitations of the structural approach. We also emphasize that complementary approaches (ion-substitution spectroscopy and electrophysiology, targeted mutagenesis, and time-resolved structural methods) will be needed to discriminate between possible permeation mechanisms.

We modified the Discussion to include the following (section **OLPVR1 transports ions via a single-file-like mechanism**):

While our atomic-resolution structures provide direct evidence for discrete Na^+ occupancy at the extracellular entry site and, under Ca^{2+}-blocked conditions, at the cytoplasmic constriction, we note important limitations of crystallography in describing the full permeation cycle. Na^+ ions in a hydrated, polar pore are expected to exhibit short residence times and partial hydration, which may result in low site occupancies and elevated apparent mobility, particularly in photoactivated intermediates that are only fractionally populated. Consequently, additional transient binding positions along the conductive pathway may remain structurally unresolved. Our data are therefore consistent with a "single-file–like" permeation regime; however, alternative scenarios cannot be excluded. Discriminating between these mechanisms will require complementary functional and spectroscopic experiments under systematic ion substitution, targeted mutagenesis of pore-lining residues, and time-resolved structural approaches.

Other comments:

Page 4, first sentence of Results. Calcium-bound structures were obtained through soaking crystals with calcium chloride. Did authors try to crystallize OLPVR1 in the presence of Ca2+, where they can control calcium concentration much better and where they may capture a somewhat different protein conformation or reveal different ion binding sites?

We appreciate this question. We attempted crystallization in the presence of Ca^{2+} by supplementing different concentrations of $CaCl_2$ (10-100 mM) into the LCP crystallization. However, we found that Ca^{2+} adversely affected the formation/stability of the LCP mesophase, resulting in crystals with substantially lower diffraction quality, which prevented reliable analysis of partially occupied cation-binding sites.

In contrast, Ca^{2+}-free conditions reproducibly yield crystals diffracting to true-atomic resolution, and these crystals tolerate post-growth $CaCl_2$ soaking sufficiently well to allow us to (i) obtain two reproducible Ca^{2+}-bound states using controlled soaking protocols and (ii) perform cryotrapping in Ca^{2+} conditions. We acknowledge that soaking can, in some cases, introduce artifacts due to lattice constraints and create ambiguity in the calcium concentration; however, in our case, the Ca^{2+}-bound structures remain highly similar to the Ca^{2+}-free structure, and the functional readouts under Ca^{2+} conditions corroborate the same binding event. We therefore believe that soaking provides reliable data on calcium binding by OLPVR1, not limiting its permeation into the channel due to lattice constraints. We have added a clarification to the Methods describing our co-crystallization attempts and the rationale for using soaking.

Page 10, line 7. Should "nearly covalent 2.1A H-bond" be replaced with "strong 2.1A H-bond"?

We revised accordingly, thank you.

Figure 1. The box surrounding the inner Ca2+ binding site is colored the same way as the box surrounding the Ca2+ surface site, while only the latter one is shown in magnified view. To avoid confusion, please change the color of the box surrounding the inner Ca2+ binding site.

We revised accordingly, thank you.

Figure 4c. Are there error bars for the absorbance measurements? If the points represent single measurements, should the measurements be repeated to be statistically reliable at each concentration? For the 1680-cm-1 band, is it possible to extend the concentration range of Ca2+ into high concentrations to estimate the Kd value?

The points in Figure 4c represent single measurements. However, as shown in Extended Daa Figure 3a, the presence-minus-absence spectrum was a mirror image of the absence-minus-presence spectrum at all Ca^{2+} concentrations. In addition, the spectral feature obtained by the flow system (Extended Data Figure 3a) was reasonably coincident with the light-minus-dark spectra (Extended Data Figure 3b). Based on these results, we believe they are statistically reliable.

A concentration higher than 50 mM was experimentally achievable. However, we did not perform such high concentrations. We believe two Kd values, 1 mM and >50 mM, are sufficient for this study.

Figure 5. Please fix the typo on line 4 of the legend.

We revised accordingly, thank you.

Figure 6. Should the upper Na+ binding site be shown on the left two panels with the dashed contour?

We revised accordingly, thank you.

Reviewer #3 (Remarks to the Author):

This manuscript investigates the calcium and sodium binding in the OLPVR1 ion channel. These results appear significant and novel. The work includes a large experimental component, on which I can only comment in a limited way. However, I can provide more detailed feedback on the computational part. Overall, the MD simulations could yield substantially more quantitative information that would better support the authors' initial hypotheses and conclusions.

Comment 1: The authors have performed a 500 ns molecular dynamics simulation to examine water, calcium, and sodium binding sites. Several situations have been analyzed as shown in Figure 7. However, no statistical data are reported to support the stated conclusions. For example, the authors write "the water molecule quickly leaves site B" but this claim should be quantified. It would be very informative to:

1. **Plot, as a function of time (over the last 200 ns of the production trajectory), the number of calcium ions, sodium ions, and water molecules occupying the binding site(s).**

The number of ions used in the modelling was always fixed and is specified for each modelling (2 Ca2+ ions, 1 Ca^{2+} and 1 Na^{+} ion, etc.). The trajectories of the ions are presented in Extended Data Figure 8. The specific claim regarding the water molecule is not supported by the newly performed simulations and was therefore fully rewritten (Results, section **Atomic resolution structure of the calcium-blocked photoactivated state of OLPVR1 reveals its inhibition mechanism**):

'Using 500-ns-long molecular dynamics simulations, we tested three potential candidates for this site: a water molecule, a calcium ion, and a sodium ion (Extended Data Figure 8). We observed that the calcium ion at site B induces a significant shift in charge distribution within the protein, attracting proximal water molecules, thereby destabilizing the calcium ion at site A, which then vacates its site within 150 ns across all runs. In contrast, both the sodium ion and the water molecule remained close to their starting positions throughout the simulation, indicating that they remained stable at site B. However, the average H-bond distances for the water molecule and the sodium ion are approximately 2.7 Å and 2.3 Å, respectively[40,41]. Besides that, the analysis of E44 side chain fluctuations indicates that the sodium ion induces the smallest fluctuations among the three candidate molecules, further pointing towards the presence of sodium in site B (Supplementary Note, Supplementary Figure 4).

While we cannot directly rule out the presence of a water molecule at site B, we believe the electron density is more likely to correspond to a sodium ion, given both its high coordination and strong hydrogen-bond interactions with neighbouring residues.'

2. **Compute and plot the residence time for calcium ions, sodium ions, and water molecules using the last 200 ns of the production trajectory. For example, the residence time of calcium and sodium when E44 is deprotonated, and E51 is protonated.**

Both analyses would strongly support the claim that the ions are indeed stable in the binding site.

We have calculated and plotted the distance from the initial position as a function of time (residence time) for the corresponding ion (or water molecule) assigned to the sites A and B. The plots can be found in the updated Extended Data Figure 8.

It is also important to note that E44 was deprotonated, and E51 was protonated in the illuminated ground and the Ca-blocked states; E44 and E51 were protonated in the non-illuminated ground state; the Schiff base was protonated. The protonation states of carboxylates matched the experimental FTIR data; thus, we do not report any additional molecular modelling experiments to avoid confusion. (Please refer to the FTIR results in the updated manuscript for further discussion)

Regarding calcium ion blocking the channeling pathway. Several conformations of the retinal cofactor are shown in Fig. 2. The caption reads "different structures". Is the retinal in the same isomeric state across all snapshots examined? Again, highly useful statistical information can be extracted from the MD trajectory.

It would be particularly insightful if the authors do compute retinal dihedral angles and bond-length alternation over time (as they do here: https://doi.org/10.1021/acs.jctc.5c01719) for the last 200 ns of the production trajectory, and discuss how the chromophore conformation is influenced by the surrounding residues.

We agree with the Reviewer that the analysis of retinal dihedral angles is an important reference. Following the suggestion, we have calculated the bond-length alterations (BLA) and planarity for all relevant trajectories. We have not observed any significant alterations, and 13-cis retinal remained stable over the modelling period (500 ns). It is important to note that we used classical force fields rather than the QM/MM modelling approach; therefore, our results are consistent within these constraints. We have added a short note in the revised manuscript addressing the Reviewer's comment. (Methods, section **Molecular dynamics simulations**)

'Retinal remained stable in the 13-cis conformation for the Ca-blocked state simulations (Supplementary Figure 10).'

References

1. Astashkin, R. *et al.* Structural insights into light-driven anion pumping in cyanobacteria. *Nat. Commun.* **13**, 6460 (2022).

2. Shibata, M. *et al.* Oligomeric states of microbial rhodopsins determined by high-speed atomic force microscopy and circular dichroic spectroscopy. *Sci. Rep.* **8**, 8262 (2018).

3. Kovalev, K. *et al.* Structure and mechanisms of sodium-pumping KR2 rhodopsin. *Sci. Adv.* **5**, eaav2671 (2019).

4. Kovalev, K. *et al.* High-resolution structural insights into the heliorhodopsin family. *Proc. Natl. Acad. Sci.* 201915888 (2020) doi:10.1073/pnas.1915888117.

5. Skopintsev, P. *et al.* Femtosecond-to-millisecond structural changes in a light-driven sodium pump. *Nature* **583**, 314–318 (2020).

6. Bratanov, D. *et al.* Unique structure and function of viral rhodopsins. *Nat. Commun.* **10**, 4939 (2019).

7. Zabelskii, D. *et al.* Viral rhodopsins 1 are an unique family of light-gated cation channels. *Nat. Commun.* **11**, 5707 (2020).

8. Baek, M., Park, T., Heo, L., Park, C. & Seok, C. GalaxyHomomer: a web server for protein homo-oligomer structure prediction from a monomer sequence or structure. *Nucleic Acids Res.* **45**, W320–W324 (2017).

9. Aoyama, M., Katayama, K. & Kandori, H. FTIR study of the wild-type and mutant proteins of a viral rhodopsin, OLPVR1. *Biophys. Physicobiology* **22**, n/a (2025).

10. Aoyama, M., Katayama, K. & Kandori, H. FTIR Spectroscopy of a Viral Rhodopsin, OLPVR2. *J. Phys. Chem. B* **130**, 223–232 (2026).

REVIEWER COMMENTS

Reviewer #1 (Remarks to the Author):

The authors conducted a thorough revision, including additional MD simulations, as requested by two of the reviewers. They have satisfactorily addressed all of my points.

We thank the Reviewer for their comments that helped to improve the manuscript

Reviewer #2 (Remarks to the Author):

The authors addressed my previous critiques and I have no further comments.

We thank the Reviewer for their comments that helped to improve the manuscript

Reviewer #3 (Remarks to the Author):

The authors have addressed most of my concerns. There is only one remaining point that needs to be fixed.

Specifically, the added statement in the main manuscript:

"Retinal remained stable in the 13-cis conformation for the Ca-blocked state simulations,"

is somewhat vague, as the term "stable" is ambiguous.

The authors should specify what they mean by stability and indicate the structural parameters on which this conclusion is based. In this case, those parameters are bond length alternation (BLA) and planarity, for which different definitions can be found in the literature for the retinal Schiff base molecule. Since the authors have adopted a specific definition of BLA and planarity, I recommend supporting this claim with a statement such as:

"We analyzed planarity and bond length alternation as defined in Ref. [10.1021/acs.jctc.5c01719], and observed that retinal remained in the 13-cis conformation in the Ca-blocked state simulations."

We thank the Reviewer for their comments that helped to improve the manuscript. We agree with the comment and we corrected the statement as suggested by the Reviewer.
